# Porous metal–organic alloys based on soluble coordination cages†

**DOI:** 10.1039/d0sc04941g

**Published:** 2020-10-19

**Authors:** Alexandra M. Antonio, Kyle J. Korman, Glenn P. A. Yap, Eric D. Bloch

**Affiliations:** Department of Chemistry & Biochemistry, University of Delaware Newark DE 19716 USA edb@udel.edu

## Abstract

Diverse strategies for the preparation of mixed-metal three-dimensional porous solids abound, although many of them lend themselves only moderate levels of tunability. Herein, we report the design and synthesis of surface functionalized permanently microporous coordination cages and their use in the isolation of mixed metal solids. Judicious alkoxide-based ligand functionalization was utilized to tune the solubility of starting copper(ii)-based cages and their resulting compatibility with the mixed-cage approach described here. We further prepared a family of isostructural molybdenum(ii) cages for a subset of the ligands. The preparation of mixed-metal cage solids proceeds under facile conditions where solutions of parent cages are mixed and product phases isolated. A suite of spectroscopic and characterization tools confirm the starting cages are intact in the amorphous product. Finally, we show that utilization of precise ligand functional groups can be used to prepare mixed cage solids that can be easily and cleanly separated into their constituent components through simple solvent washing or solvent extraction techniques.

## Abstract 2

Surface-functionalized porous coordination cages can be used to create homogeneous mixed-cage alloys with high levels of tunability and processability.

## Graphical abstract

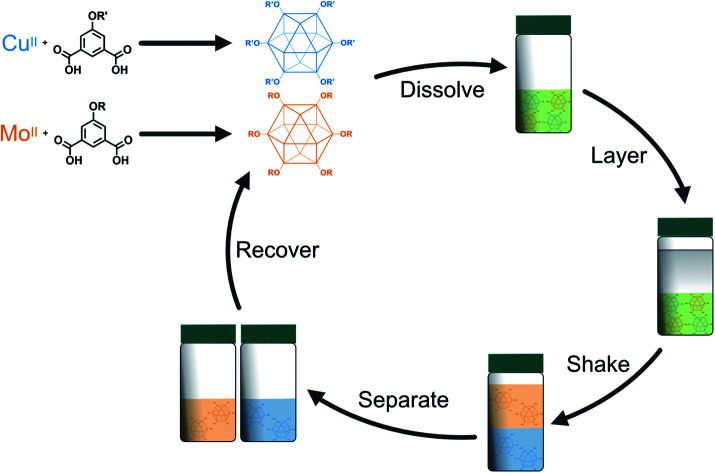

## Introduction

**Fig. 1:**
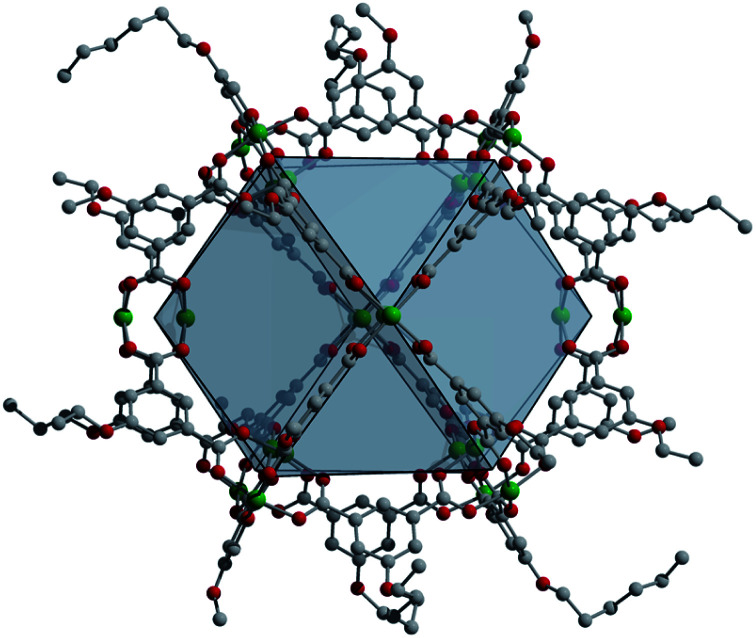
An example of an alkoxide-functionalized cuboctahedral copper cage used for the synthesis of porous coordination cage alloys. The structure, Cu_24_(5-hexoxy-bdc)_24_ features ligand functional groups on its surface that can be tuned to optimize solubility.

Permanently microporous coordination cages have seen a marked increase in their development over the past five years.^1–3^ These molecules, which are also commonly referred to as metal–organic polyhedra (MOPs) or porous coordination cages (PCCs),^4,5^ are distinct from the broader class of supramolecular containers^6–11^ in that they are stable to solvent evacuation and display permanent porosity to gases in the solid state. In this regard, they are similar to metal–organic frameworks (MOFs), the well-known and widely studied class of porous three-dimensional materials.^12,13^ For many porous coordination cages, they can be thought of as molecular analogs of metal–organic framework pores.^14^ Given their often-limited solubility,^15^ many of them are actually more MOF-like than molecular in nature.^16^ Their surface areas often pale in comparison to the record values reported for MOFs.^17^ However, even for insoluble porous cages, their molecular nature does endow them with significant advantages as compared to MOFs.^18^ Chiefly among them is their modularity and compatibility with molecular level design principles.^19–23^ These can be leveraged, for example, in synthetic routes where precise ligand functionalization can be used to tune the crystal packing and thus solid-state properties of a given cage structure.^24,25^ Soluble cages have considerable added benefits as compared to both MOFs and insoluble PCCs. Solution-based synthesis and characterization methods can be used,^26^ host-guest chemistry can be studied,^27^ and homogeneous post-synthetic functionalization can be employed.^28^ A number of researchers have used this latter strategy in the post-synthetic modification of cuboctahedral coordination cages (Fig. 1).^29^

Isophthalic acid, paddlewheel-based cuboctahedral structures have been under investigation for nearly two decades although they were initially limited to copper- and molybdenum-based structures with simple ligand functional groups.^30,31^ Relatively recently, however, there has been an influx of publications regarding these types of materials, whose related structures have been expanded to Cr, Ni, Ru, and Rh.^32–41^ In addition to this, an impressive body of work has been established regarding the incorporation of a variety of functional groups on their surface.^42^ These are typically installed prior to cage assembly, where precisely placed ligand functional groups have been used to tune charge, solubility, phase, surface area, and stability.^43–45^ More recently, the post-synthetic modification of a subset of these structures has been demonstrated where solution-stable cages can be modified with ester, ether, or amide groups.^25,41,46–48^ Cuboctahedral cages have also shown utility as building blocks for three-dimensional porous solids. It has been shown that the addition of an appropriate pillaring ligand can be used to isolate cuboctahedra-based MOFs.^49–51^ Given this, it is not surprising that these M_24_L_24_ paddlewheel structures are also present as prevalent pore types in a wide variety of MOFs.

As the organic and coordination chemistry of cuboctahedral paddlewheel-based cages has been developed, so too have new applications that have leveraged the inherent solubility of these MOF-like structures. As reported by Li *et al.*, an organic-soluble, water-insoluble charged cuboctahedron was solution processed to form a uniform honeycomb interface for potential biological applications.^52^ Other unique properties can be realized through the solvation and dispersion of these cages, exemplified by the marked increase in gas adsorption and thermal stability when dispersed inside mesoporous silica.^53^ We have recently shown that solutions of oppositely charged, permanently porous cages based on differing metal cations can be combined to afford insoluble, extended salt structures made solely of coordination cages.^54^ The syntheses of these porous salts illustrates a benefit of the use of coordination cages for the synthesis of mixed functionality materials. In targeting, for example, a mixed-metal framework, one could simply combine oppositely-charged porous cages that contain the metal cations of interest. This is in stark contrast to the synthetic methods that are required to afford mixed-metal and/or mixed-ligand MOFs.^55,56^ Often, serendipity plays a role and a mixture of metal cations in the MOF reaction mixture affords a mixed metal product. More complex design strategies have also been employed.^57^ For these, mixed-functionality ligands bind one type of metal cation during framework synthesis, leaving the other metal binding site accessible for post-synthetic modification.^58^ Metal-chelating sites can also be added to framework ligands, post-synthetically. Some MOF structures are amenable to selective post-synthetic metal exchange.^59^ For example, one of the four metal cations that make up the secondary building unit of MOF-5 can selectively be exchanged for other first-row metal cations.^60^ However, the process is time consuming and lacks a level of synthetic control as precise metal : metal ratios cannot necessarily be easily tuned.

**Fig. 2:**
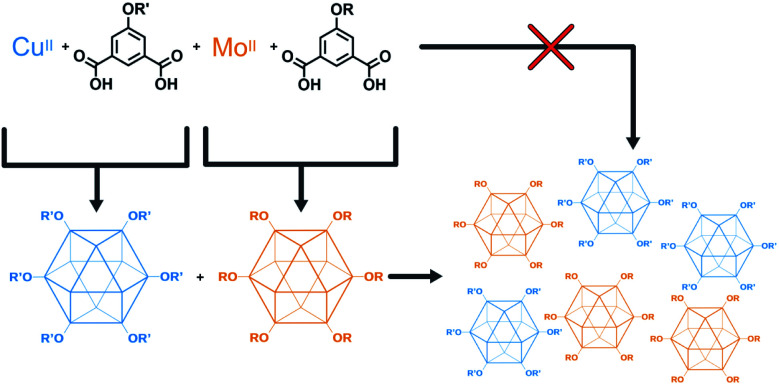
Modular approach for the synthesis of mixed-metal porous solids. Porous cages are synthesized, isolated, and combined to give a mixed-metal alloy.

The higher fidelity synthesis of mixed-functionality products has been reported for porous organic cages (POCs).^61–63^ In a subset of these, scrambled organic cages were synthesized through [4 + 6] cycloimination, with random incorporation of vicinal diamines for each molecular cage. This disrupted their ability to efficiently pack in the solid state, leading to increased gas storage capabilities. Cooper and coworkers also reported the synthesis of “porous organic alloys” where a ternary crystal was designed and prepared from a mixture of judiciously-chosen porous organic cages.^64^ This approach had the advantage in that any ratio of cages could be used to form a continuum of porous organic solid solutions. The work presented here lies at the intersection of mixed-metal MOFs and mixed-cage POCs. We report the design, synthesis, characterization, and utilization of porous coordination cage alloys (Fig. 2). These materials are comprised of alkoxide-functionalized copper and molybdenum coordination cages where the synthesis, modification, and separation of the materials is controllable based on the level of alkoxide-functionalization.

## Results and discussion

Although permanently porous coordination cages have been widely investigated as a result of their potential compatibility with molecular synthesis and characterization methods, the vast majority of cuboctahedral cages are in fact insoluble in most common organic solvents. We have recently shown that judicious ligand functionalization can be used to improve the solubility of these types of cage while still endowing them with N_2_ or CO_2_ accessible surface area in the solid state.^25^ In addition to this, ligand functional groups can be used to control the phase of isophthalic acid-based cages. An important starting point for the work outlined here is the development of a family of coordination cages that have high solubility, can be isolated for multiple metals, and maintain porosity in the solid state upon solvent removal. With the exception of Cu_24_(OPent-bdc)_24_, which is soluble in a limited number of organic solvents, the alkoxide-functionalized cages we previously reported contain insufficient alkyl chain length to impart solubility.^40^ Although the 5-dodecoxyisophthalic acid-based cage was reported some time ago by Yaghi, it has limited surface area to CO_2_ and is essentially nonporous to N_2_.^65^ To more systematically understand the relationship between alkoxide chain length, solubility, and porosity, we initiated a study wherein copper cages based on functionalized ligands from hexoxy through dodecoxy were prepared.

**Fig. 3:**
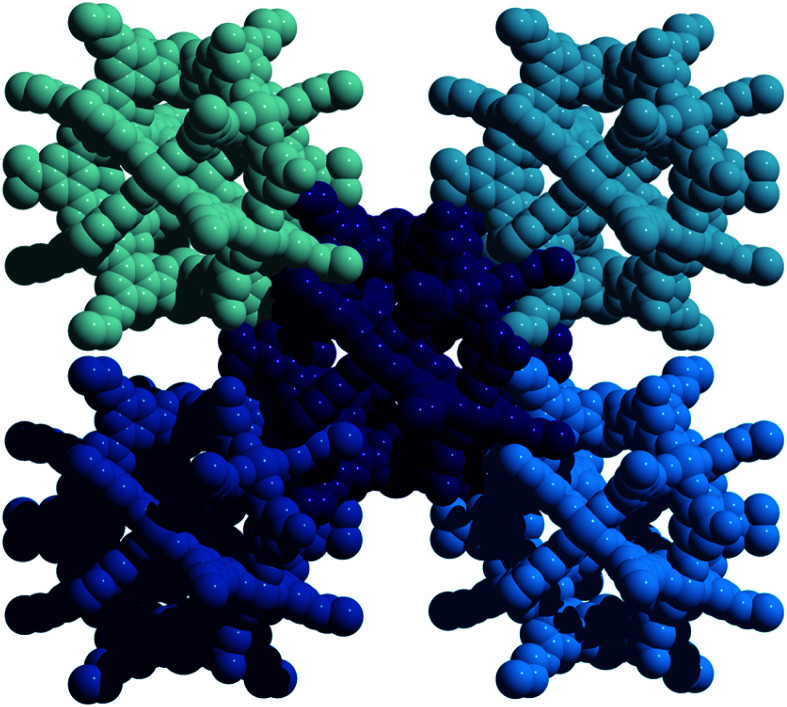
Solid-state structure of Cu_24_(5-nonoxy-bdc)_24_ as viewed down the c-axis. Subtle ligand functionalization changes tune the packing, and thus porosity, of these types of materials.

The synthesis of alkoxide-functionalized ligands is relatively straightforward where the reaction of the appropriate alkyl halide with the methyl-protected ester of 5-hydroxy isophthalate in acetone in the presence of base affords the targeted ligands in high yield. A straightforward base-catalyzed deprotection gives the free carboxylic acids. The synthesis of functionalized cuboctahedral cages is also straightforward where the reaction of ligand with copper salts in amide solvents typically gives cage in high yield. However, in order to isolate diffraction quality single crystals (Fig. 3), extensive temperature and solvent screening is necessary. Initially, equimolar metal–ligand solutions were placed in several dry baths to screen rate of cage formation as a function of temperature. Due to the disordered nature of the alkoxide chains decorating the periphery of the cages, several days of heating were necessary to afford diffraction quality single crystals. Typically, solutions with too high of an alcohol content produced crystalline powders. A simple solubility test helped indicate whether resultant products were cages or higher-dimensional solids, with the latter being insoluble. In instances where microcrystalline powder had formed, solvent ratios were then modified to give single crystals.

Analogous to the synthesis of copper cages where a wide variety of conditions can afford the target cage, general desolvation can be achieved by simply heating the samples to moderate temperatures under N_2_. However, to optimize the surface areas for these cages, a number of additional factors must be considered. We typically screen a wide variety of solvent exchange conditions where washes with volatile solvents are employed either directly or after multiple amide washes. For the seven copper cages reported, optimal surface areas were achieved after thorough methanol washes. We were able to monitor the completion of washes and activation through infrared spectroscopy. The IR spectra for all cages initially showed a strong stretch at ∼1660 cm^−1^ and a broad peak at ∼3300 cm^−1^, which were expected based on the solvent mixture used in the synthesis of the cage, *N*,*N*-dimethylformamide (carbonyl stretch) and an alcohol (–OH stretch), respectively. The copper-bound DMF molecules were replaced with methanol through subsequent washes, indicated by the loss of the carbonyl stretch and the persistence of –OH stretch in the IR spectra. Finally, the –OH stretch became absent following activation, indicating optimal activation conditions for each cage. We also screened desolvation temperatures, with N_2_ and CO_2_ isotherms recorded after successive evacuation steps. Optimal degas conditions were screened for the materials, with no discernible correlation to the length of the alkoxide tail. As could be expected the shortest chain length, Cu_24_(5-hexoxy-bdc)_24_ afforded the highest BET (Langmuir) surface area, 197 (523) m^2^ g^−1^, and was the only cage that had N_2_ accessible surface area. The CO_2_ accessible surface areas of the remaining materials were generally slightly lower with BET (Langmuir) surface areas ranging from 102 to 168 (354–771) m^2^ g^−1^.

Although as-synthesized cages showed considerable solubility in a wide range of organic solvents, activated material had decreased solubility profiles. We have generally found that solvation of the metal cation sites in porous coordination cages with amides (*e.g.* DMF, DMA, *etc.*) increases their solubility in less-polar solvents. As expected, we saw a marked increase in solubility with increasing chain length. Cu_24_(5-hexoxy-bdc)_24_ was only soluble in DMF and DMA while Cu_24_(5-dodecoxy-bdc)_24_ was highly soluble in DMF, DMA, THF, CHCl_3_, CH_2_Cl_2_, and benzene. The intermediate-length alkoxide cages had intermediate solubility, for example, Cu_24_(5-nonoxy-bdc)_24_ was highly soluble in DMF and DMA, partially soluble in THF, CH_3_Cl, CH_2_Cl_2_, and insoluble in benzene.

Given the differing solubilities of the hexoxy-, nonoxy-, and dodecoxy-bdc based cages, we targeted the synthesis of molybdenum-based cuboctahedra with these ligands. The synthesis of Mo_24_(R-bdc)_24_ cages proceeds *via* the reaction of Mo_2_(OAc)_4_ and the functionalized ligand in amide/alcohol solvent mixtures at various ratios. For instance, Mo_24_(5-hexoxy-bdc)_24_ was synthesized in an 80 : 20 DMA : MeOH ratio after heating for 2 days at 100 °C in an N_2_ glovebox. In contrast, Mo_24_(5-nonoxy-bdc)_24_ required a 90 : 10 DMF : EtOH solvent ratio in order to precipitate crystalline orange solid. Lastly, for the most soluble Mo(ii) cage of the three described, the synthesis of Mo_24_(5-dodecoxy-bdc)_24_ necessitated an 80 : 20 DMF : EtOH mixture to give crystalline solid.

Although we were unable to obtain diffraction-quality single crystals of the Mo-based materials, the diamagnetic nature of molybdenum(ii) paddlewheels confers the added advantage in that they are compatible with solution NMR studies. After synthesizing the desired Mo(ii) cages, the mother liquor was discarded and the crystalline powders were thrice washed with the respective alcohols used during synthesis. After Mo_24_(5-hexoxy-bdc)_24_ was fully exchanged with MeOH, it was dried under vacuum and dissolved in DMF-d_7_ for NMR studies (Fig. S15†). Similarly, after Mo_24_(5-nonoxy-bdc)_24_ was fully exchanged with EtOH, it was vacuum dried and dissolved in DMF-d_7_ (Fig. S18†). Finally, after Mo_24_(5-dodecoxy-bdc)_24_ was fully exchanged with EtOH, it was vacuum dried and dissolved in CDCl_3_ for ^1^H NMR (Fig. S21†).

Thermogravimetric analysis measurements suggest the Mo(ii) cages have high thermal stability with minimal mass losses up to 400 °C, while the Cu(ii) analogues decompose at much lower temperatures. As a result of this high thermal stability, fully activated Mo(ii) cages can be realized with no residual amide or alcohol solvent bound to the metal paddlewheels and no structure collapse after proper washing methods. Although the Mo(ii) cages retain porosity until ∼350 °C, with surface areas fluctuating minimally, their Cu(ii) analogues collapse and lose porosity at higher temperatures, with the Cu_24_(5-hexoxy-bdc)_24_ displaying significant surface area decrease at just 75 °C (Fig. S98†). Similar to its Cu(ii) counterpart, Mo_24_(5-hexoxy-bdc)_24_ afforded the highest BET surface area of the molybdenum-based cages at 290 m^2^ g^−1^ and was the only Mo(ii) cage that demonstrated pores accessible to N_2_. The Mo(ii) cages followed a trend where increasing the length of alkoxy chain decreased the BET surface area, with Mo_24_(5-nonoxy-bdc)_24_ and Mo_24_(5-dodecoxy-bdc)_24_ exhibiting surface areas of 172 m^2^ g^−1^ and 104 m^2^ g^−1^, respectively.

**Fig. 4:**
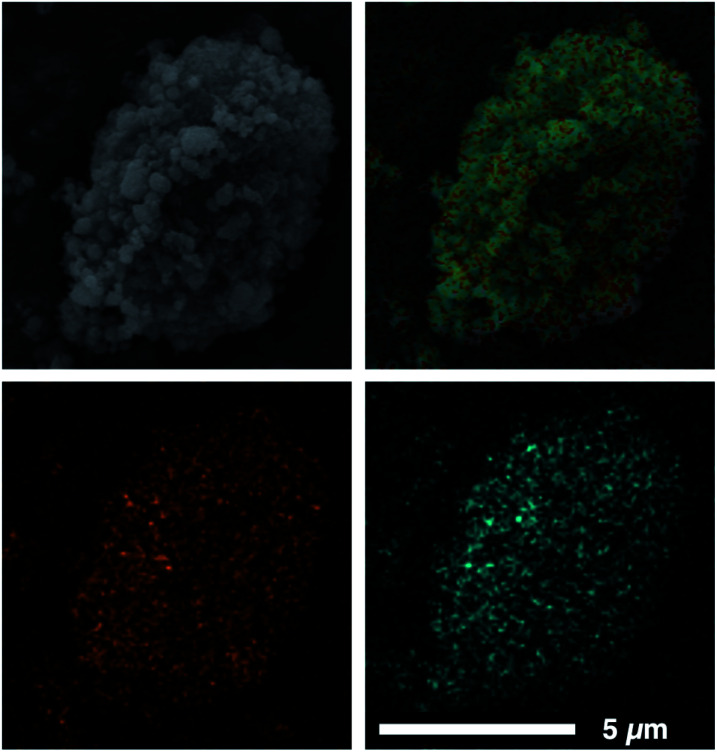
SEM image (top left) and corresponding EDX mapping (top right) of a [Cu_24_(5-dodecoxy-bdc)_24_/Mo_24_(5-dodecoxy-bdc)_24_] particle. In the EDX maps, orange and cyan represent Mo (bottom left) and Cu (bottom right), respectively, clearly showing the homogeneous distribution of metals in the particle.

**Fig. 5:**
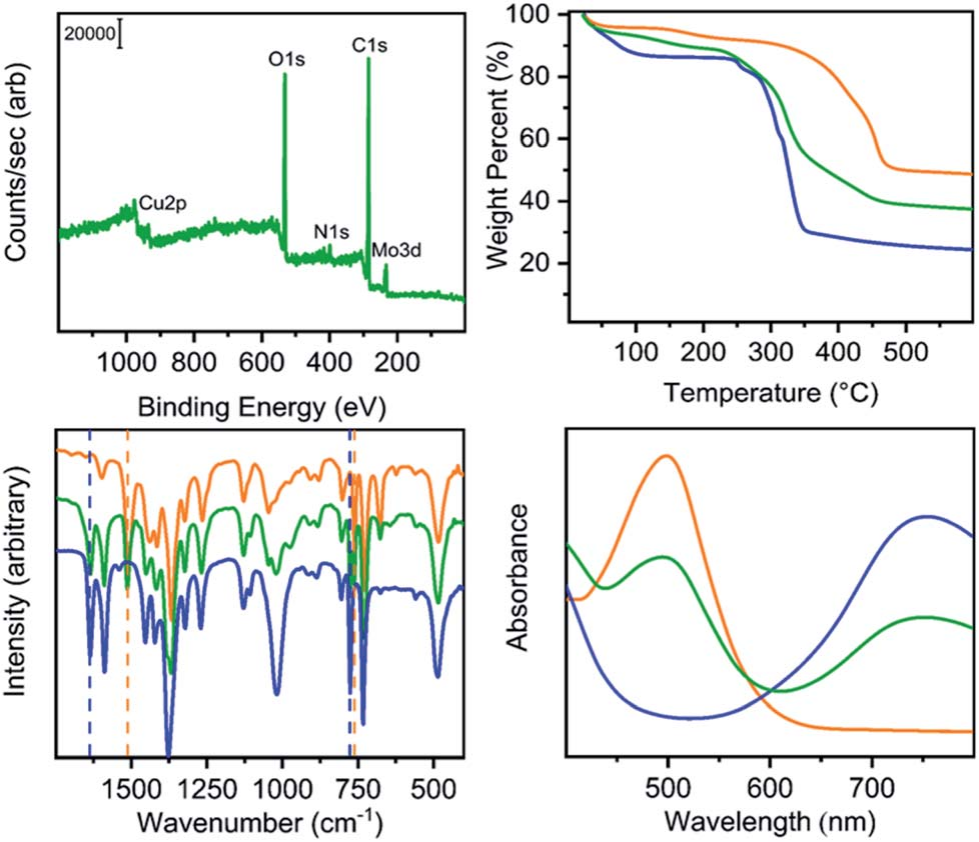
Characterization of Cu_24_(5-hexoxy-bdc)_24_ (blue), Mo_24_(5-hexoxy-bdc)_24_ (orange) and [Cu_24_(5-hexoxy-bdc)_24_/Mo_24_(5-hexoxy-bdc)_24_] green. The top left, top right, bottom left, and bottom right plots show XPS, TGA, IR, and UV-vis data, respectively. Importantly, the Cu/Mo alloy displays spectroscopic signatures of both parent cage yet bulk thermal properties (TGA) that are dissimilar to either cage.

Our previously reported syntheses of porous salts based on coordination cages afford mixed-metal cages wherein product phase is MOF-like in that it is completely insoluble in all organic solvents as a result of the high lattice energy of the product phase. Utilization of surface-functionalized cages offers an additional level of tunability as the solid phase based on alkoxide functionalized cages is still expected to be soluble in certain solvents. To prepare mixed-metal materials, we focused on M_24_(5-hexoxy-bdc)_24_, M_24_(5-nonoxy-bdc)_24_, and M_24_(5-dodecoxy-bdc)_24_ (M = Cu, Mo) as they all display appreciable solubility in solvents with varying polarity. The syntheses of [Cu_24_(5-hexoxy-bdc)_24_/Mo_24_(5-hexoxy-bdc)_24_], [Cu_24_(5-nonoxy-bdc)_24_/Mo_24_(5-nonoxy-bdc)_24_], and [Cu_24_(5-dodecoxy-bdc)_24_/Mo_24_(5-dodecoxy-bdc)_24_] proceeded similarly where equimolar quantities of each cage were dissolved in an appropriate solvent (hexoxy = DMF, nonoxy = THF, dodecoxy = benzene) and in the case of [Cu_24_(5-hexoxy-bdc)_24_/Mo_24_(5-hexoxy-bdc)_24_] and [Cu_24_(5-nonoxy-bdc)_24_/Mo_24_(5-nonoxy-bdc)_24_] precipitated with anhydrous methanol. [Cu_24_(5-dodecoxy-bdc)_24_/Mo_24_(5-dodecoxy-bdc)_24_] was isolated by simply subliming frozen benzene from a homogeneous solution to afford a porous solid. For all three materials, the spectroscopic features displayed by the resultant solids are a weighted average of those of the parent cages (Fig. 5 and S53–S66†) while the thermal gravimetric analysis and differential scanning calorimetry results are significantly different than either parent cage (Fig. 5 and S67–S79†). As expected, the survey XPS scan indicates the presence of both copper and molybdenum in the isolated samples. In a similar manner, the vibrational and electronic spectra for the products indicate the parent cages are intact when isolated as mixed materials. IR spectra clearly indicate vibrational features that are specific to either Cu_24_(5-R-bdc)_24_ or Mo_24_(5-R-bdc)_24_ are present in the products. Finally, elemental mapping experiments (Fig. 4 and S86–S97†) confirm a truly homogenous dispersion of copper and molybdenum in product particles.

Although the product phases are poorly crystalline, they display CO_2_ accessible surface areas that are on par with the values of the pure-metal parent cages and range from 293–549 m^2^ g^−1^ (Langmuir). In order to probe the accessibility of the Mo^2+^ cations in these materials, we turned to 273 K oxygen isotherms as the reactivity of molybdenum(ii)-based paddlewheels toward O_2_ is well known. As expected, Cu_24_(R-bdc)_24_ (R = hexoxy, nonoxy, dodecoxy) adsorbs minimal O_2_ at 273 K and 1.0 bar. In contrast, isotherms measured under the same conditions for Mo_24_(R-bdc)_24_ (R = hexoxy, nonoxy, dodecoxy) indicate a strong interaction with O_2_ as the adsorption isotherms are incredibly steep before turning over at approximately 0.5 mmol g^−1^ (Fig. S111†). The porous alloy materials based on mixtures of these cages display saturation O_2_ capacities that fall between those of the metal-pure cages (Fig. S112†). The structural stability of the Mo(ii) cages allows for recovery of the material even after chemisorption of O_2_, where the cage can be reactivated, releasing bound O_2_ and retaining CO_2_ accessible surface area (Table S3†).

Previous work has shown that these types of cages are isolable with mixed-metal paddlewheel units.^37^ We turned to a combination of experiments to rule out metal cation exchange in these materials and to confirm the products are best described as [Cu_24_(R-bdc)_24_]/[Mo_24_(R-bdc)_24_] as compared to [Cu_12_Mo_12_(R-bdc)_24_] or [(CuMo)_12_(R-bdc)_24_]. Importantly, absorption features in solution-based UV-vis spectra of the product phase correspond to those in the pure-cage solutions (Fig. S53†), which is consistent with a lack of metal cation exchange in a given paddlewheel. This does not, however, rule out the presence of mixed-paddlewheel cages. To investigate this more thoroughly, we undertook a variety of ligand and metal/ligand exchange experiments.

It has been previously shown that copper-based cuboctahedral cages are amenable to ligand exchange chemistry.^12^ To determine the propensity for Mo_24_(R-bdc)_24_ to engage in ligand exchange reactions a number of control experiments were performed. Mo_24_(5-hexoxy-bdc)_24_ was dissolved in DMF, followed by addition of 5-nonoxy isophthalic acid, heating to 100 °C, and allowing the sample to cool to room temperature before precipitating out a product with methanol. The isolated product was only soluble in DMF, suggesting the absence of more solubilizing functional groups. ^1^H NMR of the product phase confirms this interpretation as pure Mo_24_(5-hexoxy-bdc)_24_ cage was isolated (Fig. S22†). Similarly, we found that Mo_24_(5-nonoxy-bdc)_24_/H_2_dodecoxy-bdc and Mo_24_(5-dodecoxy-bdc)_24_/H_2_hexoxy-bdc mixtures did not participate in ligand exchange reactions (Fig. S23 and S24†).

To rule out ligand exchange in the presence of cages based on both metals, we combined Cu_24_(5-hexoxy-bdc)_24_ and Mo_24_(5-nonoxy-bdc)_24_ in DMF, heated to 100 °C until both cages dissolved then added excess methanol upon cooling to precipitate product. The resulting brown solid was thoroughly washed with methanol and dried under dynamic vacuum. Addition of THF to the dried powder selectively dissolved Mo_24_(5-nonoxy-bdc)_24_, leaving Cu_24_(5-hexoxy-bdc)_24_ powder. The purity of Mo_24_(5-nonoxy-bdc)_24_ was confirmed *via*^1^H NMR spectroscopy (Fig. S25†). However, the Mo(ii) cage could not be fully separated from the Cu_24_(5-hexoxy-bdc)_24_ cage as seen *via* UV-vis spectroscopy (Fig. S26†). Since the porous alloy was precipitated from DMF and washed with methanol, there was residual DMF coordinated to the metal paddlewheels that were not fully removed. Consequently, the usually THF-insoluble Cu_24_(5-hexoxy-bdc)_24_ began to dissolve in THF upon subsequent washing intended to remove Mo_24_(5-nonoxy-bdc)_24_, rendering this separation technique unsatisfactory. Therefore, in order to fully separate the porous cage alloy, we utilized an extraction technique to promote phase transfer and subsequent recovery of parent cages.

**Fig. 6:**
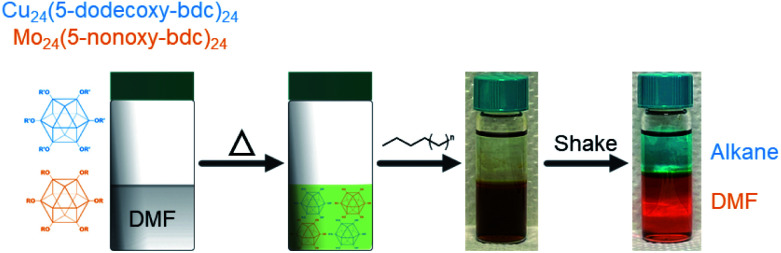
The separation of coordination cage-based porous alloys where solubility of functionalized cages can be used for straightforward solvent extractions. Here a DMF mixture of Cu_24_(5-dodecoxy-bdc)_24_ and Mo_24_(5-nonoxy-bdc)_24_ is layered with hexane and shaken to afford cleanly separated cage solutions.

An important and practically useful embodiment of this example involves the preparation of porous cage alloys based on both different metals and different ligands. Here one could, for example, leverage the differing solubility of each cage in the preparation of porous cage alloys to recover phase-pure cage after synthesis of the product phase. In an analogous manner to the synthesis of ligand-pure mixed metal cages, we prepared DMF solutions of [Cu_24_(R-bdc)_24_]/[Mo_24_(R-bdc)_24_] where R = 12/6; 12/9; 6/12; or 9/12 carbon chain alkoxides. In all cases, and analogous to the pure ligand mixtures, the resulting homogeneous solutions are dark brown. Substituent cages can be separated, however, by simply layering a linear alkane solvent on the DMF solution, shaking the vial, and pipette separate product-containing layers where the longer chain alkoxide cages are soluble in the alkane phase while the 5-nonoxy-bdc and 5-hexoxy-bdc cages remain in DMF layer (Fig. 6). NMR characterization of the dissolved or digested (Mo and Cu, respectively) cages rule out metal or ligand exchange reactions and confirm cage purity (Fig. S27–S34†). Given the broad tunability of the solubility of these types of cages, and by extension those of cages based on different divalent metal cations, we expect this approach to be highly tunable for novel syntheses of mixed-metal porous solids.

## Conclusions

Mixed-metal, permanently porous metal–organic materials are of high interest for a wide variety of applications. However, there remain challenges for the straightforward and tunable syntheses of these materials. We have shown that soluble permanently microporous coordination cages can be used for the preparation of mixed-metal products. Precise surface functionalization of alkoxide-based cages allows for tuning of cage solubility which, in turn, can be used for the construction or deconstruction of porous materials. A variety of spectroscopic tools were used to confirm that the parent cages persist after incorporation into amorphous alloys. Importantly, this approach gives the added benefit that product phase formation is reversible and starting cages are isolable by simple solvent washing or extraction procedures. We expect that the mixed-cage design principles outlined here can be used to prepare a nearly unlimited number of mixed-metal porous solids with complete control over metal ratios in product phase. Importantly, this approach can be utilized for families of cages that display increased thermal, hydrolytic, and chemical stabilities as compared to paddlewheel-based cages.

## Conflicts of interest

There are no conflicts to declare.

## Supplementary Material

SC-011-D0SC04941G-s001

SC-011-D0SC04941G-s002
