# Supplementary material for: Porous metal–organic alloys based on soluble coordination cages
Source: Chem Sci. 2020 Oct 19;11(46):12540–6. doi: 10.1039/d0sc04941g (PMC8163318; doi:10.1039/d0sc04941g)
Supplement: SC-011-D0SC04941G-s001 [file SC-011-D0SC04941G-s001.pdf]

Electronic Supplementary Material (ESI) for Chemical Science.
This journal is © The Royal Society of Chemistry 2020

Electronic Supplementary Information for:

Porous metal-organic alloys based on soluble coordination cages

Alexandra M. Antonio,†[a] Kyle J. Korman,†[a] Glenn P. A. Yap[a] and Eric D. Bloch*[a]

[a] Department of Chemistry & Biochemistry, University of Delaware, Newark, DE 19716, USA
† These authors are joint first authors and contributed equally.

List of Contents

Ligand Synthesis and Characterization	S3
Solvothermal Cage Syntheses	S9
Syntheses of Mixed-Metal Cage Alloys	S12
Cage ^{1}H-NMR Spectra	S13
Mo (II) Cage Ligand Exchange Controls	S21
Phase Transfer/Separation of Alloys	S24
X-Ray Crystallography	S34
Powder X-Ray Diffraction	S37
UV-Vis Spectra	S43
Infrared Spectra	S44
Thermogravimetric Analysis	S50
Differential Scanning Calorimetry	S55
X-Ray Photoelectron Spectroscopy (XPS) Data	S57
SEM/EDX Data	S60
BET Surface Area Isotherms	S72
O_2 Isotherms	S84
Table of Surface Areas	S86
Table of Cage Solubility	S86

Experimental Section

General Considerations. All reagents were obtained from commercial vendors and used without purification, excluding solvents used for air sensitive procedures. Air sensitive products required solvent obtained from a solvent purification system. These were dispensed and then stored in a glove box over 3 Å or 4 Å sieves. Low-pressure adsorption measurements were obtained on a Micromeritics Tristar 3000. Powder X-ray Diffraction Patterns were taken using a Bruker D8 XRD with a Cu K_α radiation (1.54 nm) housed in the Advanced Materials Characterization Lab at the University of Delaware. ^{1}H-NMR spectra were taken on an AV 400 spectrometer. Infrared (IR) spectra were carried out with a Bruker ALPHA II instrument. UV-Vis experiments were carried out with an AvaSpec-ULS2048CL. Elemental Analyses were performed on a Thermo Fisher K-Alpha XPS at the University of Delaware's Surface Analysis Facility. Scanning Electron Microscope images and Energy-dispersive X-ray Spectra were taken using an SEM/FIB Auriga 60 housed in the Advanced Materials Characterization Lab at the University of Delaware.

Ligand Syntheses:

Synthesis of 5-hexoxy-1,3-benzenedicarboxylic acid (5-hexoxy-H_2bdc): Dimethyl 5-hydroxyisophthalate (4 g, 19 mmol), potassium carbonate (2.9 g, 21 mmol), and 1-iodohexane (3.1 mL, 21 mmol) were added to a 250 mL RBF with 80 mL of acetone and stirred at 40 ˚C for 12 hours. The colorless solid was removed via vacuum filtration. The filtrate was added back to the RBF along with potassium hydroxide (2.46 g, 43.8 mmol) in 80 mL of DI H_2O. The solution was stirred for 12 hours at 50 ˚C. The solution was concentrated to remove the acetone, then acidified to a pH of 1. The white precipitate was isolated via vacuum filtration then dried overnight at 60 ˚C to yield 69 % final product. ^{1}H-NMR (400 MHz, DMSO): δ = 13.29 (s, 2H, OH), 8.05 (t, 1H, CH arom), 7.62 (d, 2H, CH arom), 4.06 (t, 2H, OCH_2), 1.73 (pentet, 2H, CH2), 1.35 (m, 6H, C_3H_6), 0.87 (t, 3H, CH_3).

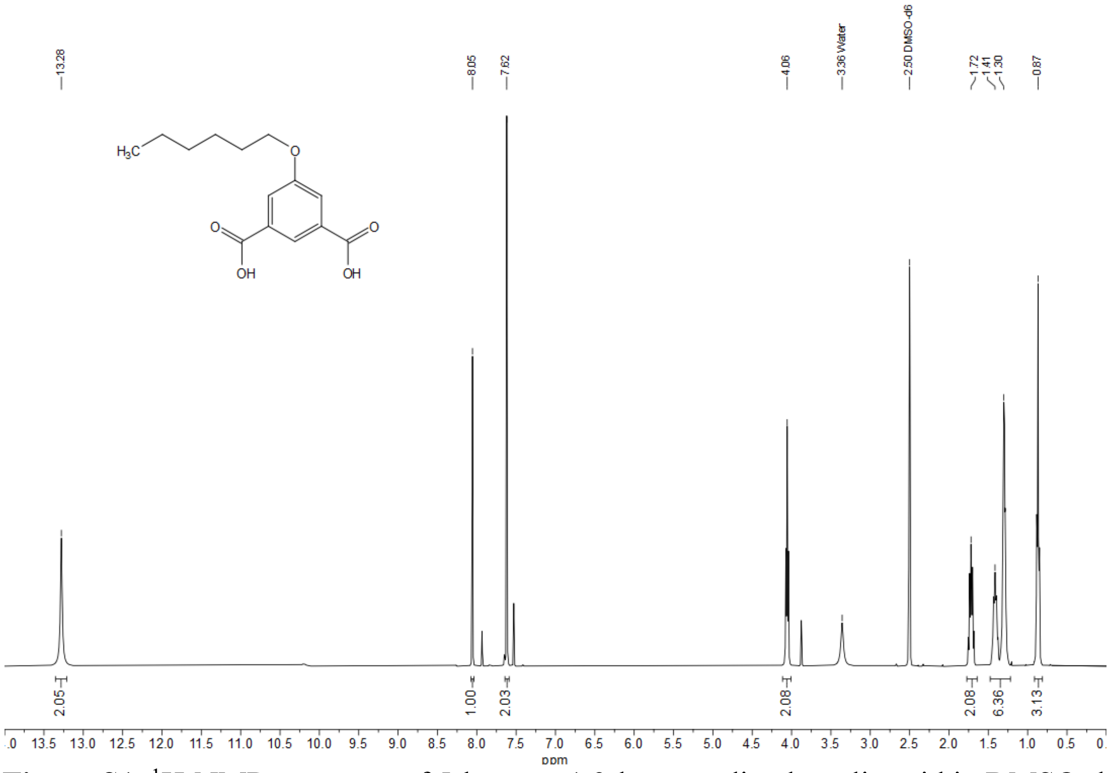

Figure S1. ^{1}H-NMR spectrum of 5-hexoxy-1,3-benzenedicarboxylic acid in DMSO-d_6.

Synthesis of 5-heptoxy-1,3-benzenedicarboxylic acid (5-heptoxy-H_2bdc):
Dimethyl 5-hydroxyisophthalate (4 g, 19 mmol), potassium carbonate (2.9 g, 21 mmol), and 1-iodoheptane (3.44 mL, 21 mmol) were added to a 250 mL RBF with 80 mL of acetone and stirred at 40 ˚C for 12 hours. The colorless solid was removed via vacuum filtration. The filtrate was added back to the RBF along with potassium hydroxide (2.46 g, 43.8 mmol) in 80 mL of DI H_2O. The solution was stirred for 12 hours at 50 ˚C. The solution was concentrated to remove the acetone, then acidified to a pH of 1. The white precipitate was isolated via vacuum filtration then dried overnight at 60 ˚C to yield 65 % final product. ^{1}H-NMR (400 MHz, DMSO): δ = 13.27 (s, 2H, OH), 8.05 (t, 1H, CH arom), 7.62 (d, 2H, CH arom), 4.06 (t, 2H, OCH_2), 1.72 (pentet, 2H, CH_2), 1.34 (m, 8H, C_4H_8), 0.86 (t, 3H, CH_3).

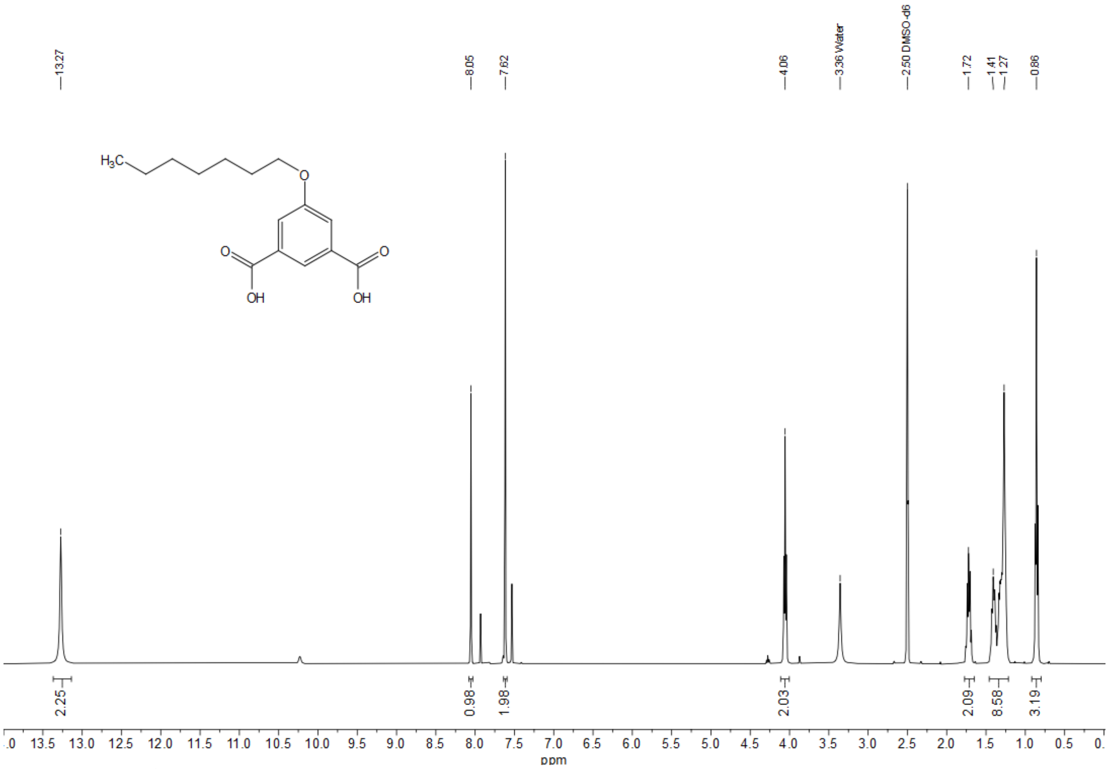

Figure S2. ^{1}H-NMR spectrum of 5-heptoxy-1,3-benzenedicarboxylic acid in DMSO-d_6.

Synthesis of 5-octoxy-1,3-benzenedicarboxylic acid (5-octoxy-H_2bdc):
Dimethyl 5-hydroxyisophthalate (4 g, 19 mmol), potassium carbonate (2.9 g, 21 mmol), and 1-iodooctane (3.8 mL, 21 mmol) were added to a 250 mL RBF with 80 mL of acetone and stirred at 40 ˚C for 12 hours. The colorless solid was removed via vacuum filtration. The filtrate was added back to the RBF along with potassium hydroxide (2.46 g, 43.8 mmol) in 80 mL of DI H_2O. The solution was stirred for 12 hours at 50 ˚C. The solution was concentrated to remove the acetone, then acidified to a pH of 1. The white precipitate was isolated via vacuum filtration then dried overnight at 60 ˚C to yield 78 % final product. ^{1}H-NMR (400 MHz, DMSO): δ = 13.27 (s, 2H, OH), 8.05 (t, 1H, CH arom), 7.61 (d, 2H, CH arom), 4.05 (t, 2H, OCH_2), 1.71 (pentet, 2H, CH_2), 1.32 (m, 10H, C_5H_{10}), 0.84 (t, 3H, CH_3).

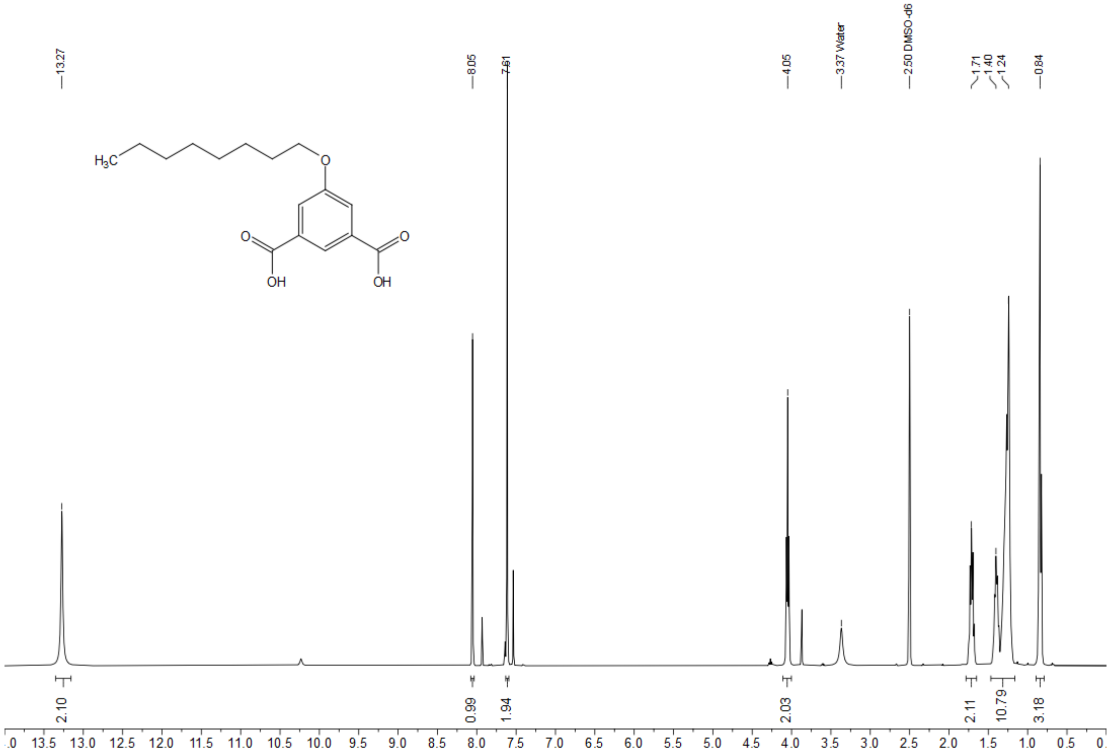

Figure S3. ^{1}H-NMR spectrum of 5-octoxy-1,3-benzenedicarboxylic acid in DMSO-d_6.

Synthesis of 5-nonoxy-1,3-benzenedicarboxylic acid (5-nonoxy-H_2bdc):
Dimethyl 5-hydroxyisophthalate (4 g, 19 mmol), potassium carbonate (2.9 g, 21 mmol), and 1-iodononane (4.14 mL, 21 mmol) were added to a 250 mL RBF with 80 mL of acetone and stirred at 40 ˚C for 12 hours. The colorless solid was removed via vacuum filtration. The filtrate was added back to the RBF along with potassium hydroxide (2.46 g, 43.8 mmol) in 80 mL of DI H_2O. The solution was stirred for 12 hours at 50 ˚C. The solution was concentrated to remove the acetone, then acidified to a pH of 1. The white precipitate was isolated via vacuum filtration then dried overnight at 60 ˚C to yield 64 % final product. ^{1}H-NMR (400 MHz, DMSO): δ = 13.27 (s, 2H, OH), 8.05 (t, 1H, CH arom), 7.62 (d, 2H, CH arom), 4.05 (t, 2H, OCH_2), 1.71 (pentet, 2H, CH_2), 1.31 (m, 12H, C_6H_{12}), 0.84 (t, 3H, CH_3).

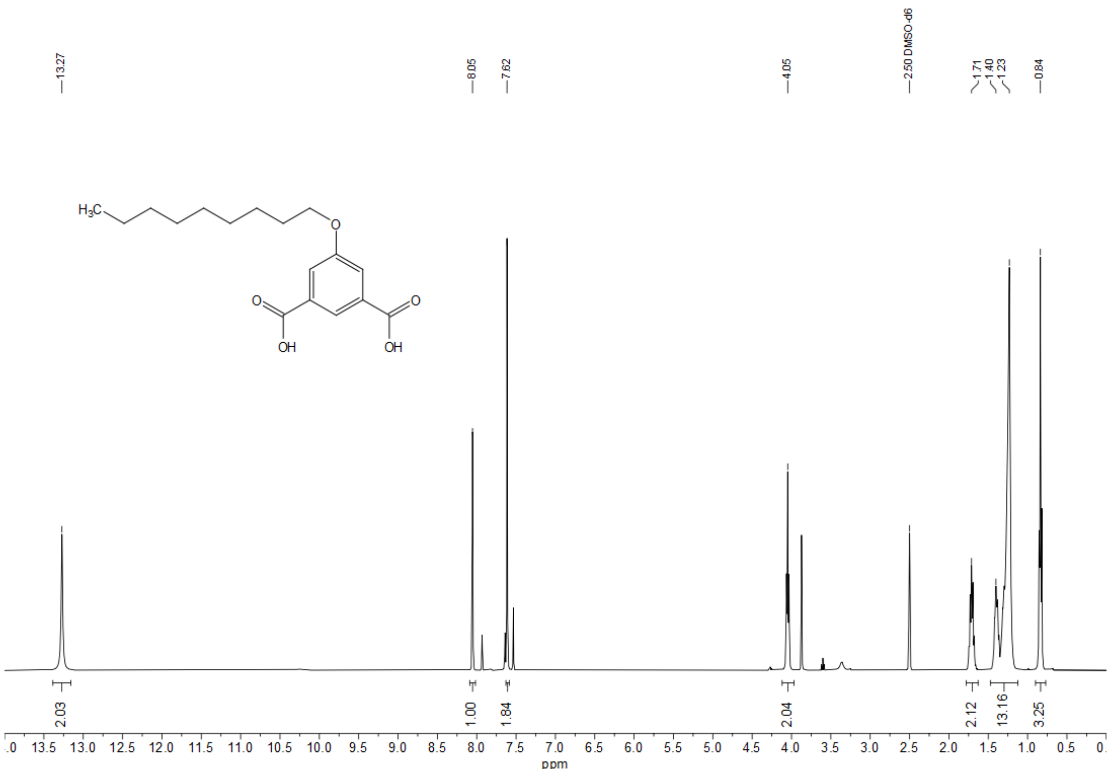

Figure S4. ^{1}H-NMR spectrum of 5-nonoxy-1,3-benzenedicarboxylic c in DMSO-d_6.

Synthesis of 5-decoxy-1,3-benzenedicarboxylic acid (5-decoxy-H_2bdc):
Dimethyl 5-hydroxyisophthalate (4 g, 19 mmol), potassium carbonate (2.9 g, 21 mmol), and 1-iododecane (4.48 mL, 21 mmol) were added to a 250 mL RBF with 80 mL of acetone and stirred at 50 ˚C for 12 hours. The colorless solid was removed via vacuum filtration. The filtrate was added back to the RBF along with potassium hydroxide (2.46 g, 43.8 mmol) in 80 mL of DI H_2O. The solution was stirred for 12 hours at 50 ˚C. The solution was concentrated to remove the acetone, then acidified to a pH of 1. The white precipitate was isolated via vacuum filtration then dried overnight at 60 ˚C to yield 76 % final product. ^{1}H-NMR (400 MHz, DMSO): δ = 13.27 (s, 2H, OH), 8.06 (s, 1H, CH arom), 7.61 (s, 2H, CH arom), 4.04 (t, 2H, OCH_2), 1.71 (pentet, 2H, CH_2), 1.31 (m, 14H, C_7H_{14}), 0.83 (t, 3H, CH_3).

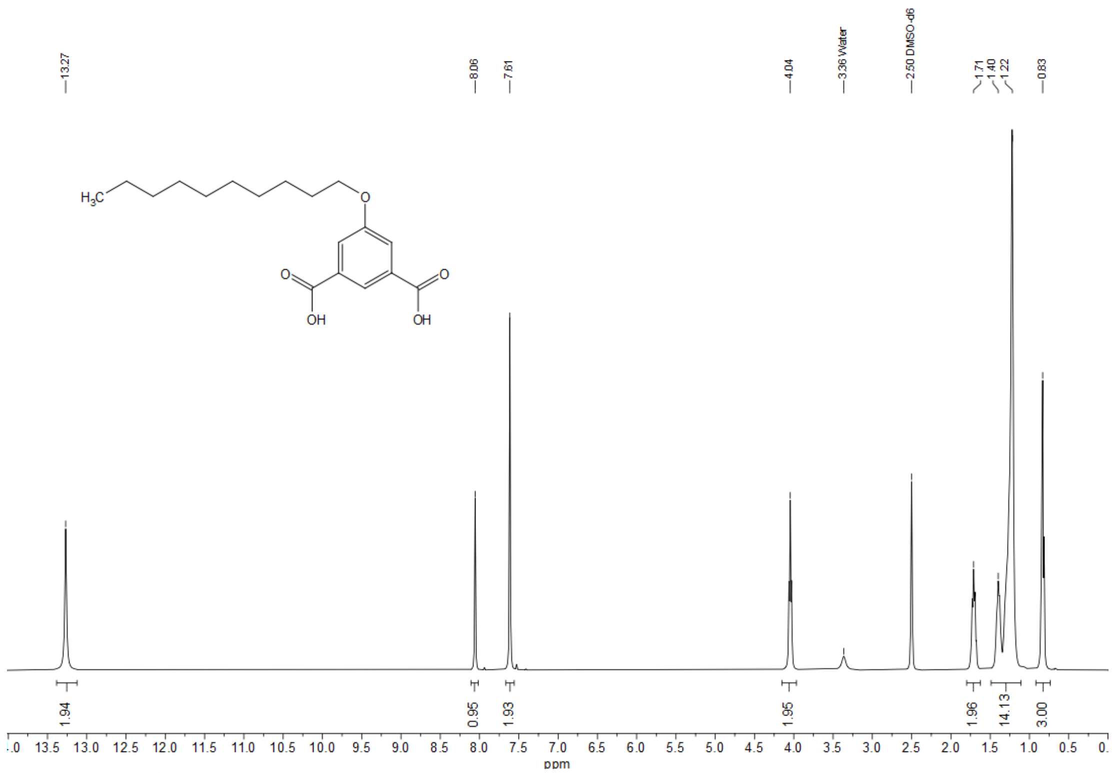

Figure S5. ^{1}H-NMR spectrum of 5-decoxy-1,3-benzenedicarboxylic acid in DMSO-d_6.

Synthesis of 5-undecoxy-1,3-benzenedicarboxylic acid (5-undecoxy-H_2bdc):
Dimethyl 5-hydroxyisophthalate (4 g, 19 mmol), potassium carbonate (2.9 g, 21 mmol), and 1-iodoundecane (4.86 mL, 21 mmol) were added to a 250 mL RBF with 80 mL of acetone and stirred at 50 ˚C for 12 hours. The colorless solid was removed via vacuum filtration. The filtrate was added back to the RBF along with potassium hydroxide (2.46 g, 43.8 mmol) in 80 mL of DI H_2O. The solution was stirred for 12 hours at 50 ˚C. The solution was concentrated to remove the acetone, then acidified to a pH of 1. The white precipitate was isolated via vacuum filtration then dried overnight at 60 ˚C to yield 91% final product. ^{1}H-NMR (400 MHz, DMSO): δ = 13.28 (s, 2H, OH), 8.05 (t, 1H, CH arom), 7.61 (d, 2H, CH arom), 4.05 (t, 2H, OCH_2), 1.71 (pentet, 2H, CH_2), 1.31 (m, 16H, C_8H_{16}), 0.83 (t, 3H, CH_3).

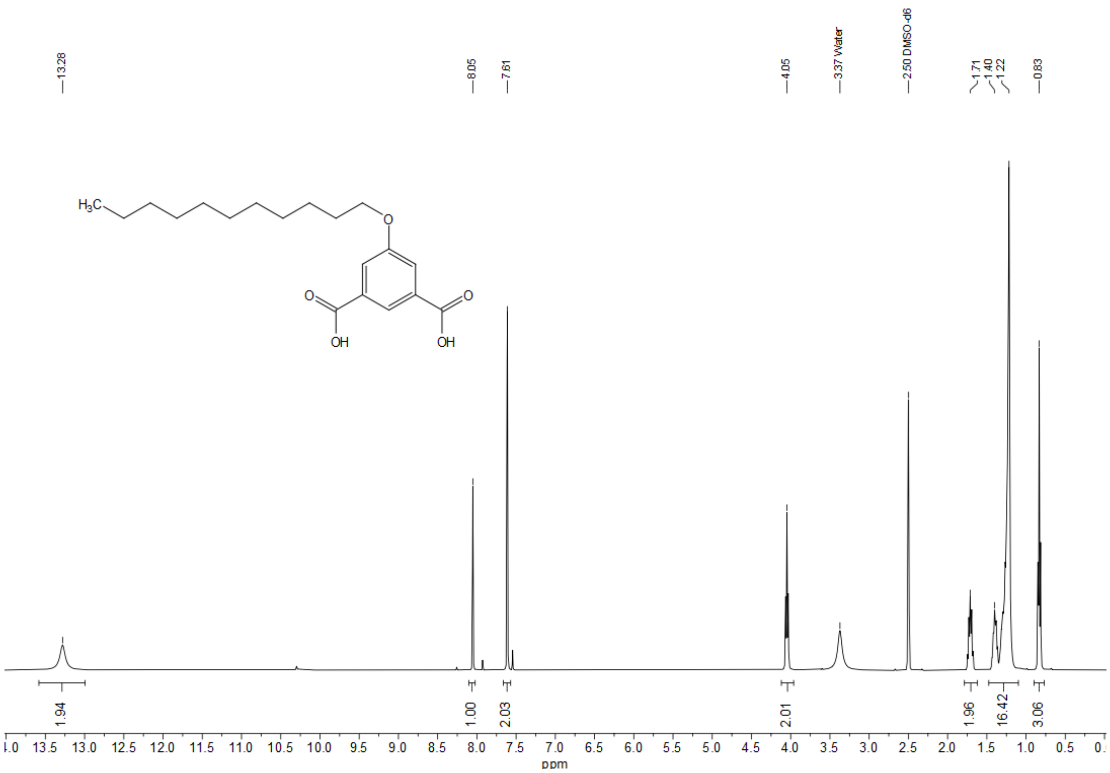

Figure S6. ^{1}H-NMR spectrum of 5-undecoxy-1,3-benzenedicarboxylic acid in DMSO-d_6.

Synthesis of 5-dodecoxy-1,3-benzenedicarboxylic acid (5-dodecoxy-H_2bdc):

Dimethyl 5-hydroxyisophthalate (4 g, 19 mmol), potassium carbonate (2.9 g, 21 mmol), and 1-iodododecane (5.18 mL, 21 mmol) were added to a 250 mL RBF with 80 mL of acetone and stirred at 50 ˚C for 12 hours. The colorless solid was removed via vacuum filtration. The filtrate was added back to the RBF along with potassium hydroxide (2.46 g, 43.8 mmol) in 80 mL of DI H_2O. The solution was stirred for 12 hours at 50 ˚C. The solution was concentrated to remove the acetone, then acidified to a pH of 1. The white precipitate was isolated via vacuum filtration then dried overnight at 60 ˚C to yield 79% final product. ^{1}H-NMR (400 MHz, DMSO): δ = 8.06 (t, 1H, CH arom), 7.59 (d, 2H, CH arom), 4.04 (t, 2H, OCH_2), 1.71 (pentet, 2H, CH_2), 1.31 (m, 18H, C_9H_{18}), 0.83 (t, 3H, CH_3).

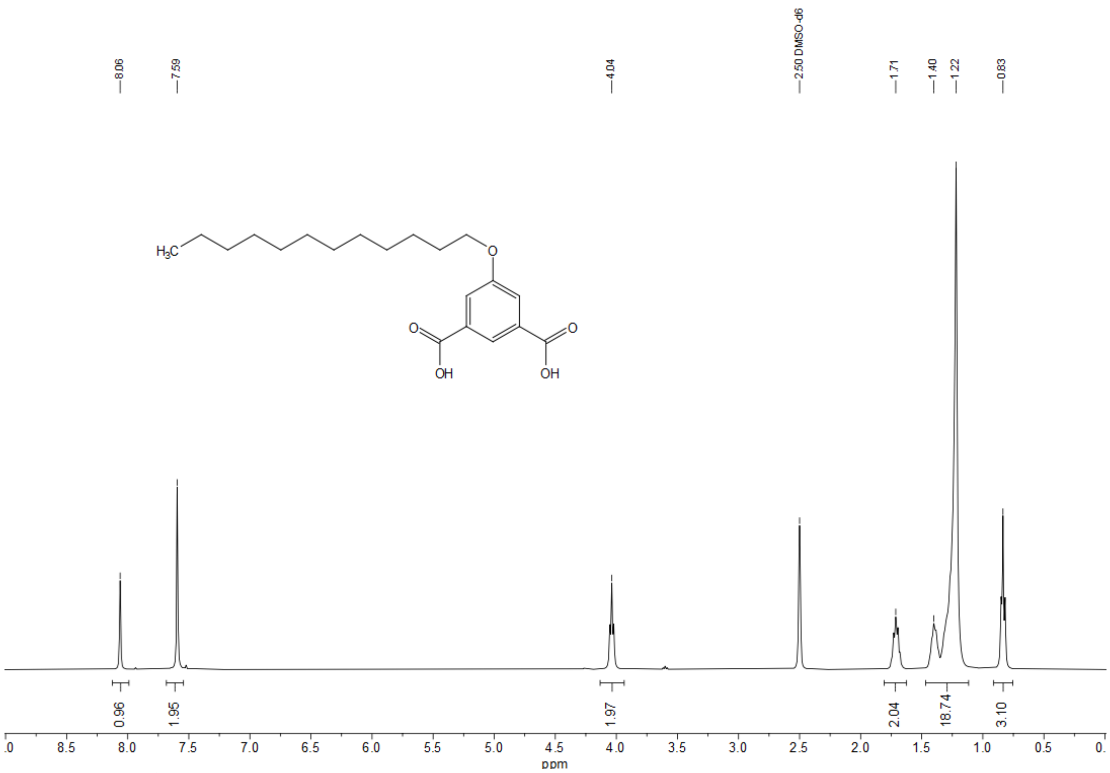

Figure S7. ^{1}H-NMR spectrum of 5-dodecoxy-1,3-benzenedicarboxylic in DMSO-d_6.

__Cage Synthesis__:

Synthesis of Cu_{24}(5-hexoxy-bdc)$_{24}$. $Cu(NO_3)_2$•2.5H_2O (0.754 g, 3.24 mmol) and 5-hexoxy-1,3-benzenedicarboxylic acid (0.862 g, 3.24 mmol) were dissolved in DMF (72 mL) and ethanol (228 mL) in a 500 mL jar and sealed with a Teflon-lined cap. The resulting solution was then heated at 80 ˚C for 3 days resulting in blue, rhombohedral shaped crystals and blue powder. The residual DMF/ethanol solution was decanted, and the crystals were washed with methanol. The methanol was decanted and fresh methanol was added and decanted 3 additional times over the course of 3 days to fully exchange and remove any DMF. The sample was then dried under vacuum to remove solvent and activated for gas adsorption measurements.

Synthesis of Cu_{24}(5-heptoxy-bdc)$_{24}$. $Cu(NO_3)_2$•2.5H_2O (0.754 g, 3.24 mmol) and 5-heptoxy-1,3-benzenedicarboxylic acid (0.908 g, 3.24 mmol) were dissolved in DMF (288 mL) and methanol (72 mL). The solution (15 mL) was dispersed in 20 mL scintillation vials (x20) and sealed with a Teflon-lined cap. The vials were then heated at 60 ˚C for 5 days resulting in blue, plate shaped crystals. The residual DMF/methanol solution was decanted, and the crystals were combined and then washed with methanol. The methanol was decanted and fresh methanol was added and decanted 3 additional times over the course of 3 days to fully exchange and remove any DMF. The sample was then dried under vacuum to remove solvent and activated for gas adsorption measurements.

Synthesis of Cu_{24}(5-octoxy-bdc)$_{24}$. $Cu(NO_3)_2$•2.5H_2O (0.116 g, 0.5 mmol) and 5-octoxy-1,3-benzenedicarboxylic acid (0.147 g, 0.5 mmol) were dissolved in DMF (2 mL) and ethanol (13 mL) in a 20 mL scintillation vial sealed with a Teflon-lined cap. The vial was then heated at 60 ˚C for

3 days resulting in a blue powder. The residual DMF/ethanol solution was decanted, and the material was washed with methanol. The methanol was decanted and fresh methanol was added and decanted 3 additional times over the course of 3 days to fully exchange and remove any DMF. The sample was then dried under vacuum to remove solvent and activated for gas adsorption measurements.

Synthesis of Cu_{24}(5-nonoxy-bdc)$_{24}$ bulk material. $Cu(NO_3)_2$•$2.5H_2O$ (0.344 g, 1.48 mmol) and 5-nonoxy-1,3-benzenedicarboxylic acid (0.456 g, 1.48 mmol) were dissolved in DMF (87.6 mL) and methanol (49.3 mL) in a 250 mL jar and sealed with a Teflon-lined cap. The resulting solution was then heated at 60 ˚C for 3 days resulting in a mixture of blue, rhombohedral shaped crystals and a blue powder. The residual DMF/methanol solution was decanted, and the material was washed with methanol. The methanol was decanted and fresh methanol was added and decanted 3 additional times over the course of 3 days to fully exchange and remove any DMF. The sample was then dried under vacuum to remove solvent and activated for gas adsorption measurements.

Synthesis of Cu_{24}(5-decoxy-bdc)$_{24}$ single crystals. $Cu(NO_3)_2$•$2.5H_2O$ (0.0063 g, 0.027 mmol) and 5-decoxy-1,3-benzenedicarboxylic acid (0.0087 g, 0.027 mmol) were dissolved in DMF (2.4 mL) and ethanol (0.6 mL) in a 4 mL vial and sealed with a Teflon-lined cap. The resulting solution was then heated at 80 ˚C for 3 days resulting in blue, rhombohedral shaped crystals. The residual DMF/ethanol solution was decanted, and the crystals were washed with methanol. The methanol was decanted and fresh methanol was added and decanted 3 additional times over the course of 3 days to fully exchange and remove any DMF. The sample was then dried under vacuum to remove solvent and activated for gas adsorption measurements.

Synthesis of Cu_{24}(5-decoxy-bdc)$_{24}$ bulk material. $Cu(NO_3)_2$•$2.5H_2O$ (0.126 g, 0.54 mmol) and 5-decoxy-1,3-benzenedicarboxylic acid (0.174 g, 0.54 mmol) were dissolved in DMF (12 mL) and ethanol (3 mL) in a 20 mL scintillation vial and sealed with a Teflon-lined cap. The resulting solution was then heated at 80 ˚C for 3 days resulting in a blue powder. The residual DMF/ethanol solution was decanted, and the material was washed with methanol. The methanol was decanted and fresh methanol was added and decanted 3 additional times over the course of 3 days to fully exchange and remove any DMF. The sample was then dried under vacuum to remove solvent and activated for gas adsorption measurements.

Synthesis of Cu_{24}(5-undecoxy-bdc)$_{24}$. $Cu(NO_3)_2$•$2.5H_2O$ (0.058 g, 0.25 mmol) and 5-undecoxy-1,3-benzenedicarboxylic acid (0.084 g, 0.25 mmol) were dissolved in DMF (12 mL) and ethanol (3 mL) in a 20 mL scintillation vial and sealed with a Teflon-lined cap. The resulting solution was then heated at 80 ˚C for 3 days resulting in a blue powder. The residual DMF/ethanol solution was decanted, and the material was washed with methanol. The methanol was decanted and fresh methanol was added and decanted 3 additional times over the course of 3 days to fully exchange and remove any DMF. The sample was then dried under vacuum to remove solvent and activated for gas adsorption measurements.

Synthesis of Cu_{24}(5-dodecoxy-bdc)$_{24}$. Procedure was modified from a previously published synthesis.[1] Accordingly, $Cu(OAc)_2$•H_2O (0.082 g, 0.41 mmol) and 5-dodecoxy-3,5-benzenedicarboxylic acid (0.147 g, 0.42 mmol) were dissolved separately in DMF (10 mL). The two solutions were subsequently combined. Methanol (15 mL) was added to the solution to

precipitate out the cage as a blue powder. The supernatant was decanted and fresh methanol was added and decanted 3 additional times over the course of 3 days to fully exchange and remove any DMF. The sample was then dried under vacuum to remove solvent and activated for gas adsorption measurements.

Synthesis of Crystalline Mo_{24}(5-hexoxy-bdc)$_{24}$. $Mo_2(OAc)_4$ (0.1849 g, 0.432 mmol) and 5-hexoxy-1,3-benzenedicarboxylic acid (0.1150 g, 0.432 mmol) were added to a 20 mL scintillation vial and dissolved in 9.6 mL DMA and 2.4 mL MeOH. Upon heating at 100 °C in an N_2 glovebox for 2 days, crystalline powder was formed. The powder was decanted of excess mother liquior and washed with anhydrous methanol three times, exchanging the solvent every 8 hours, and dried under vacuum to yield 0.1199g of an orange powder.

Synthesis of Mo_{24}(5-heptoxy-bdc)$_{24}$. $Mo_2(OAc)_4$ (0.0462 g, 0.108 mmol) and 5-heptoxy-1,3-benzenedicarboxylic acid (0.0303 g, 0.108 mmol) were added to a 20 mL scintillation vial and dissolved in 3 mL of DMF upon heating at 100 °C in a dry bath in an N_2 glovebox. About 15 mL of anhydrous methanol was used to precipitate out orange powder. The vial was centrifuged down and the powder was washed with anhydrous methanol three times, exchanging the solvent every 8 hours. It was dried under vacuum to yield an orange powder.

Synthesis of Mo_{24}(5-octoxy-bdc)$_{24}$. $Mo_2(OAc)_4$ (0.0462 g, 0.108 mmol) and 5-octoxy-1,3-benzenedicarboxylic acid (0.0318 g, 0.108 mmol) were added to a 20 mL scintillation vial and dissolved in 3 mL of DMF upon heating at 100 °C in a dry bath in an N_2 glovebox. About 15 mL of anhydrous methanol was used to precipitate out orange powder. The vial was centrifuged down and the powder was washed with anhydrous methanol three times, exchanging the solvent every 8 hours, and dried under vacuum to yield an orange powder.

Synthesis of Crystalline Mo_{24}(5-nonoxy-bdc)$_{24}$. $Mo_2(OAc)_4$ (0.1849 g, 0.432 mmol) and 5-nonoxy-1,3-benzenedicarboxylic acid (0.1332 g, 0.432 mmol) were added to a 20 mL scintillation vial and dissolved in 10.8 mL DMF and 1.2 mL EtOH. Upon heating at 100 °C in an N_2 glovebox for 2 days, crystalline powder was formed. The powder was decanted of excess mother liquior and washed with anhydrous methanol three times, exchanging the solvent every 8 hours. It was dried under vacuum to yield 0.0914g of an orange powder.

Synthesis of Mo_{24}(5-decoxy-bdc)$_{24}$. $Mo_2(OAc)_4$ (0.0462 g, 0.108 mmol) and 5-decoxy-1,3-benzenedicarboxylic acid (0.0348 g, 0.108 mmol) were added to a 20 mL scintillation vial and dissolved in 3 mL of DMF upon heating at 100 °C in a dry bath in an N_2 glovebox. About 15 mL of anhydrous methanol was used to precipitate out orange powder. The vial was centrifuged down and the powder was washed with anhydrous methanol three times, exchanging the solvent every 8 hours, and dried under vacuum to yield an orange powder.

Synthesis of Mo_{24}(5-undecoxy-bdc)$_{24}$. $Mo_2(OAc)_4$ (0.0462 g, 0.108 mmol) and 5-undecoxy-1,3-benzenedicarboxylic acid (0.0363 g, 0.108 mmol) were added to a 20 mL scintillation vial and dissolved in 3 mL of DMF upon heating at 100 °C in a dry bath in an N_2 glovebox. About 15 mL of anhydrous methanol was used to precipitate out orange powder. The vial was centrifuged down and the powder was washed with anhydrous methanol three times, exchanging the solvent every 8 hours, and dried under vacuum to yield an orange powder.

Synthesis of Crystalline Mo_{24}(5-dodecoxy-bdc)$_{24}$. $Mo_2(OAc)_4$ (0.1849 g, 0.432 mmol) and 5-dodecoxy-1,3-benzenedicarboxylic acid (0.1514 g, 0.432 mmol) were added to a 20 mL scintillation vial and dissolved in 9.6 mL DMF and 2.4 mL EtOH. Upon heating at 100 °C in an N_2 glovebox for 2 days, crystalline powder was formed. The powder was decanted of excess mother liquor and washed with anhydrous methanol three times, exchanging the solvent every 8 hours. It was dried under vacuum to yield 0.1182g of an orange powder.

Synthesis of Cu/Mo Cage Alloys:

Synthesis of Cu/Mo(5-hexoxy-bdc). Mo_{24}(5-hexoxy-bdc)$_{24}$ (0.0360 g, 0.1 mmol) and Cu_{24}(5-hexoxy-bdc)$_{24}$ (0.0327 g, 0.1 mmol) were added to a 20 mL scintillation vial and dissolved in ~5mL of DMF. About 15 mL of anhydrous methanol was used to precipitate out a brown powder. The vial was centrifuged down and the powder was washed with anhydrous methanol three times, exchanging the solvent every 8 hours, and dried under vacuum to yield 0.0614g of a brown powder.

Synthesis of Cu/Mo(5-nonoxy-bdc). Mo_{24}(5-nonoxy-bdc)$_{24}$ (0.0402 g, 0.1 mmol) and Cu_{24}(5-nonoxy-bdc)$_{24}$ (0.0369 g, 0.1 mmol) were added to a 20 mL scintillation vial and dissolved in ~5mL of THF. About 15 mL of anhydrous methanol was used to precipitate out green powder. The vial was centrifuged down and the powder was washed with anhydrous methanol three times, exchanging the solvent every 8 hours, and dried under vacuum to yield 0.065g of a green powder.

Synthesis of Cu/Mo(5-dodecoxy-bdc). Mo_{24}(5-dodecoxy-bdc)$_{24}$ (0.0444 g, 0.1 mmol) and Cu_{24}(5-dodecoxy-bdc)$_{24}$ (0.0411 g, 0.1 mmol) were added to a 20 mL scintillation vial and dissolved in ~5mL of benzene. The green solution was pipetted into a gas adsorption tube and freeze dried using liquid N_2. A fluffy green powder (0.0639 g) was formed after the frozen cage was thawed slowly by immersing the tube in an ice bath under dynamic vacuum.

<u>Copper(II) Cage NMR Spectra</u>:
In order to obtain NMR, all copper(II) cages were digested via DCl and dissolved in DMSO-d_6 after activation.

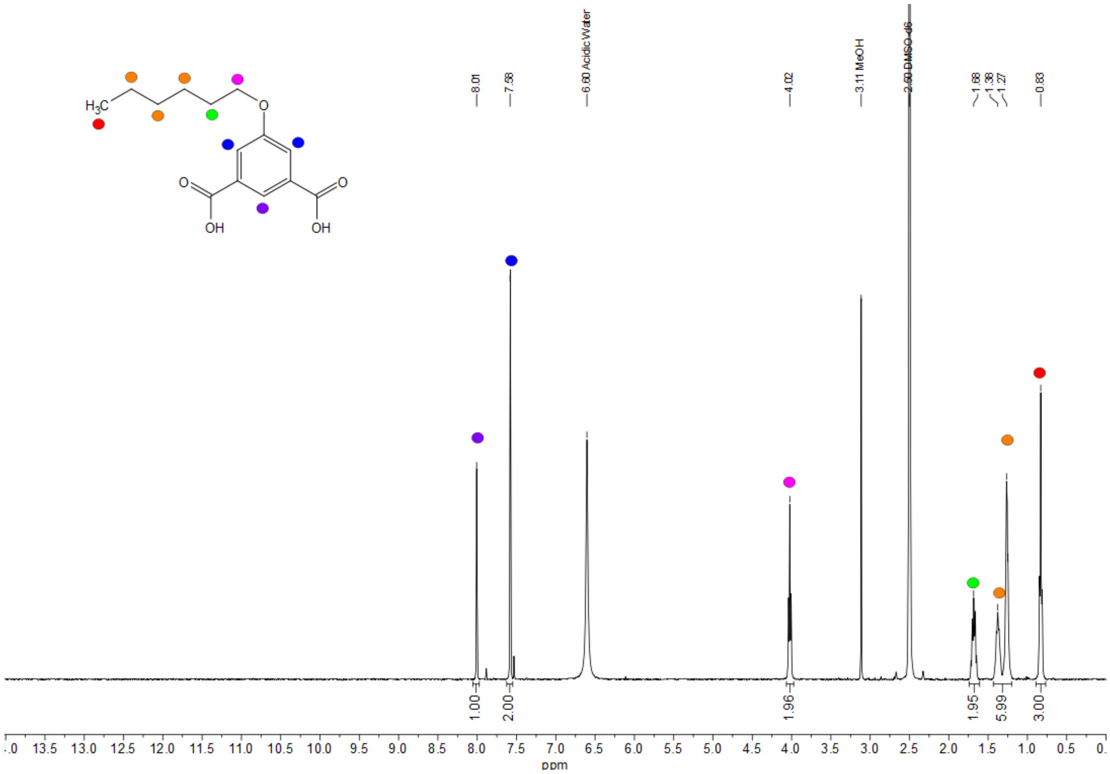

Figure S8. ^{1}H-NMR spectrum of digested Cu_{24}(5-hexoxy-bdc)$_{24}$ in DMSO-d_6.

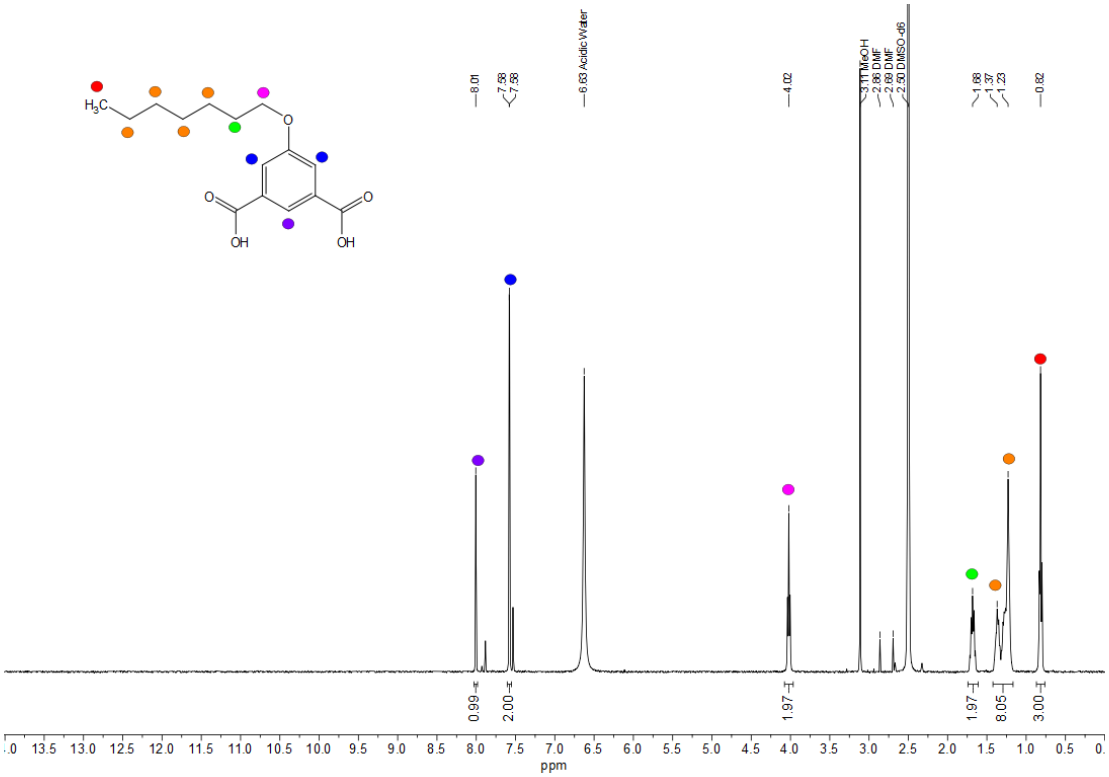

Figure S9. ^{1}H-NMR spectrum of digested Cu_{24}(5-heptoxy-bdc)$_{24}$ in DMSO-d_6.

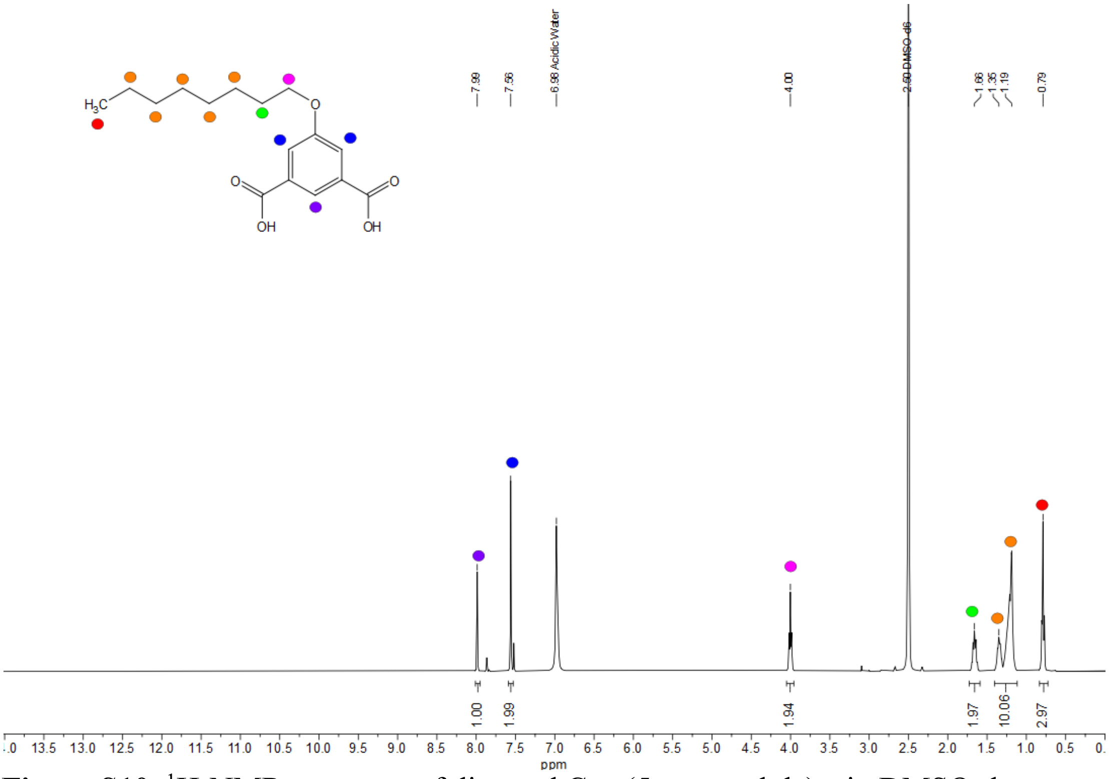

Figure S10. ^{1}H-NMR spectrum of digested Cu_{24}(5-octoxy-bdc)$_{24}$ in DMSO-d_6.

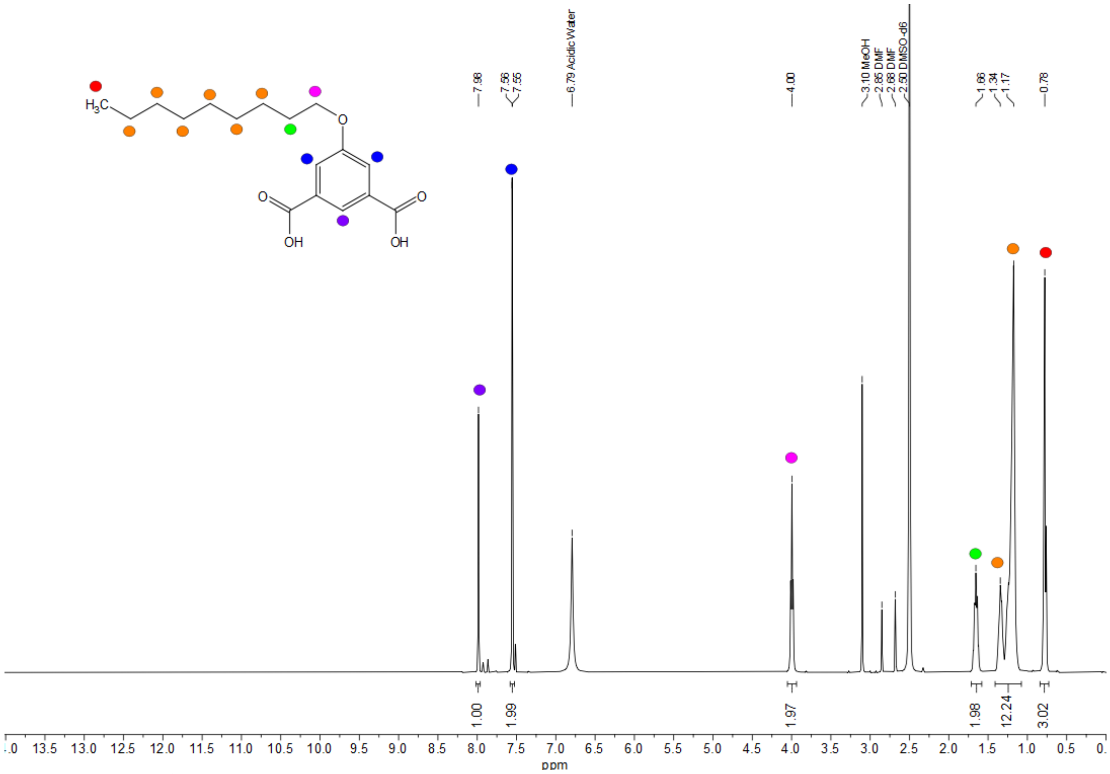

Figure S11. ^{1}H-NMR spectrum of digested Cu_{24}(5-nonoxy-bdc)$_{24}$ in DMSO-d_6.

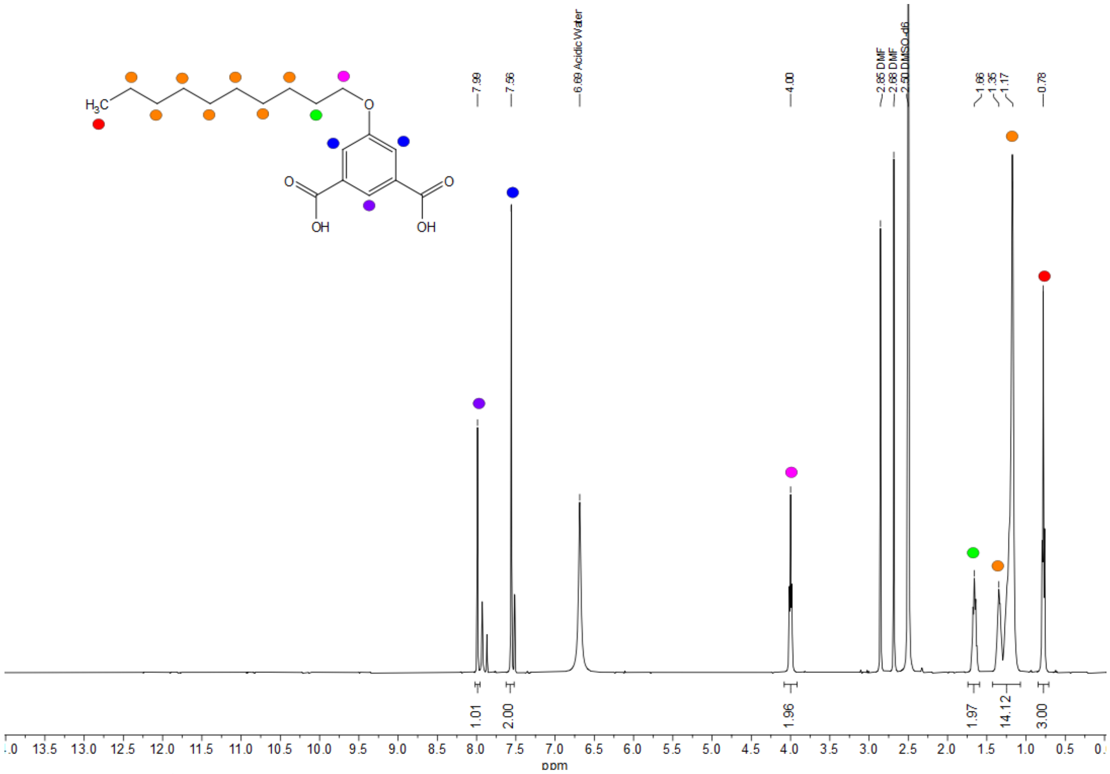

Figure S12. ^{1}H-NMR spectrum of digested Cu_{24}(5-decoxy-bdc)$_{24}$ in DMSO-d_6.

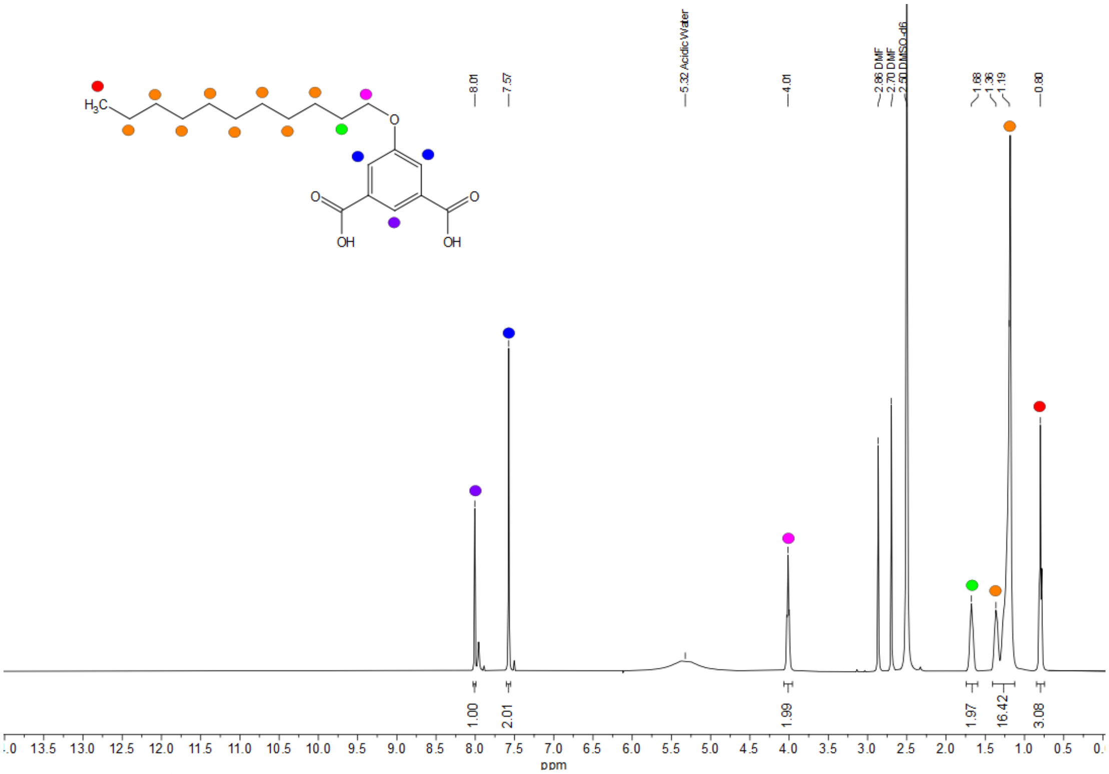

Figure S13. ^{1}H-NMR spectrum of digested Cu_{24}(5-undecoxy-bdc)$_{24}$ in DMSO-d$_6$.

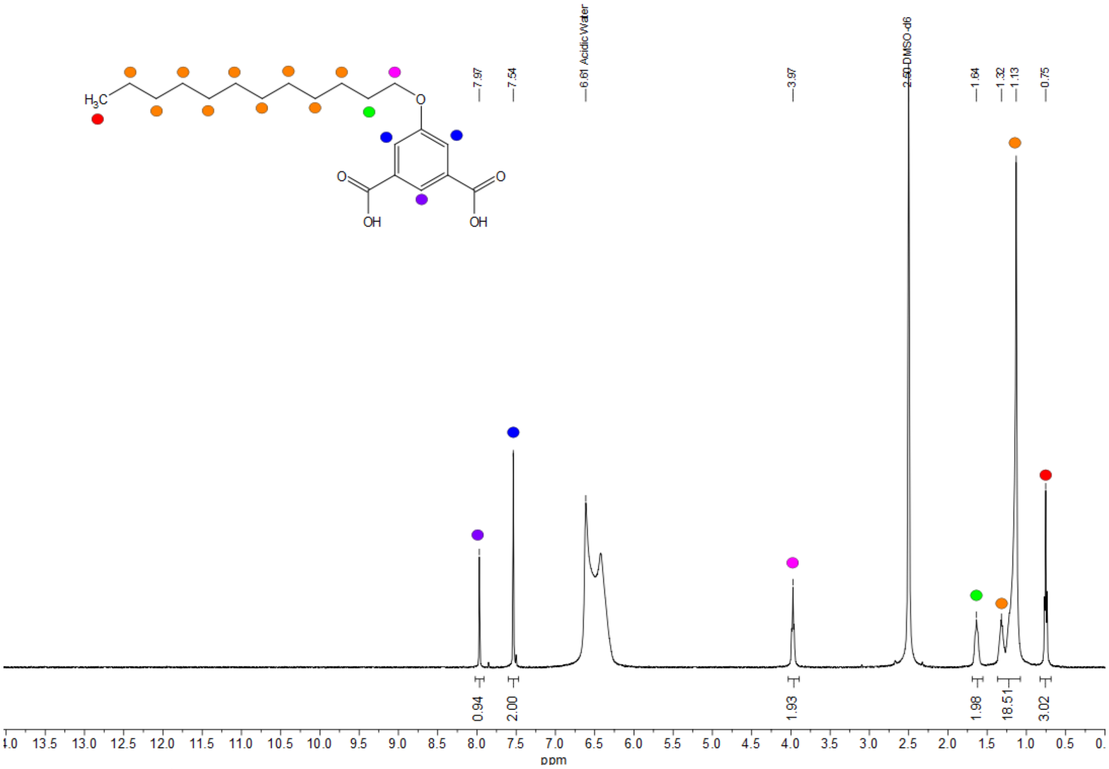

Figure S14. ^{1}H-NMR spectrum of digested Cu_{24}(5-dodecoxy-bdc)$_{24}$ in DMSO-d$_6$.

Molybdenum(II) Cage NMR Spectra:

In order to obtain the most soluble cages for NMR, all molybdenum(II) cages were crashed out from DMF and washed three times with MeOH or EtOH at room temperature.

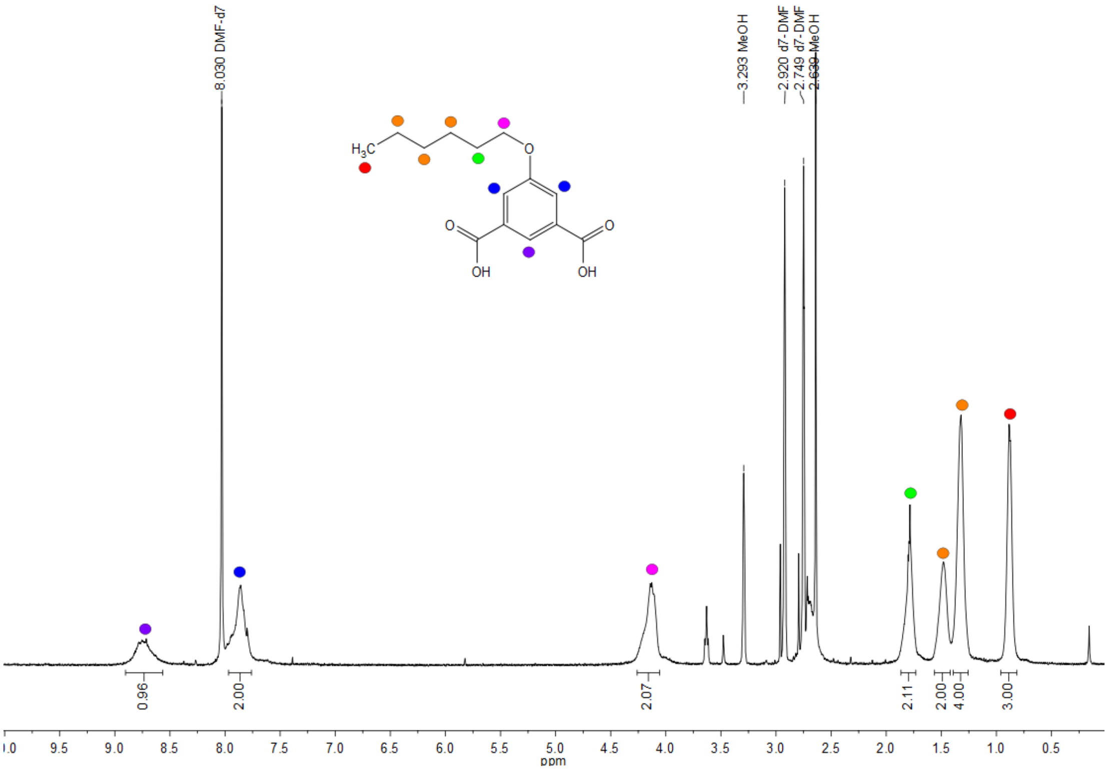

Figure S15. ^{1}H-NMR spectrum of Mo_{24}(5-hexoxy-bdc)$_{24}$ in DMF-d_7.

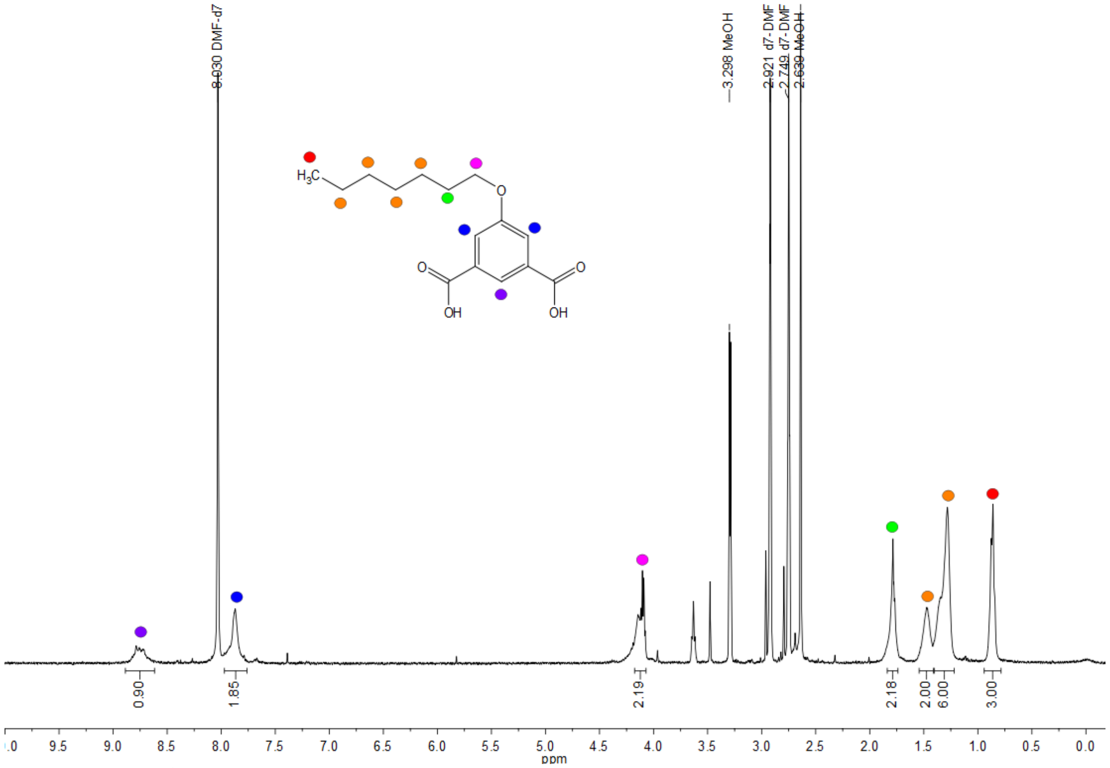

Figure S16. ^{1}H-NMR spectrum of Mo_{24}(5-heptoxy-bdc)$_{24}$ in DMF-d_7.

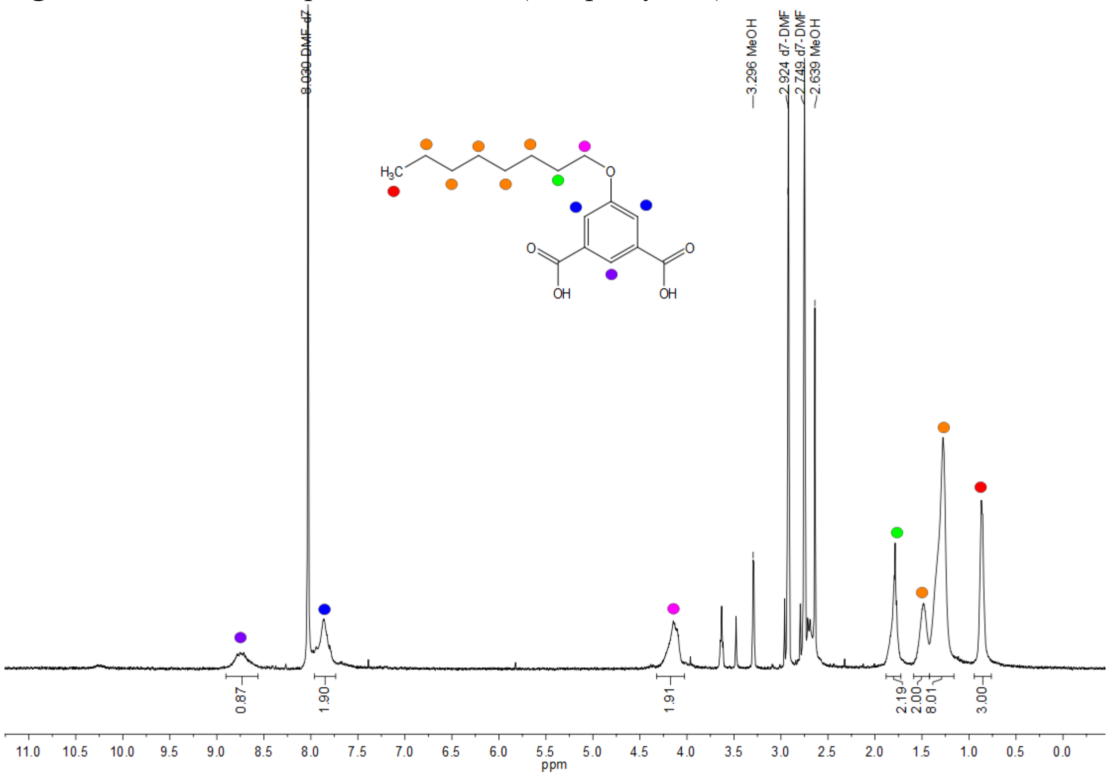

Figure S17. ^{1}H-NMR spectrum of Mo_{24}(5-octoxy-bdc)$_{24}$ in DMF-d_7.

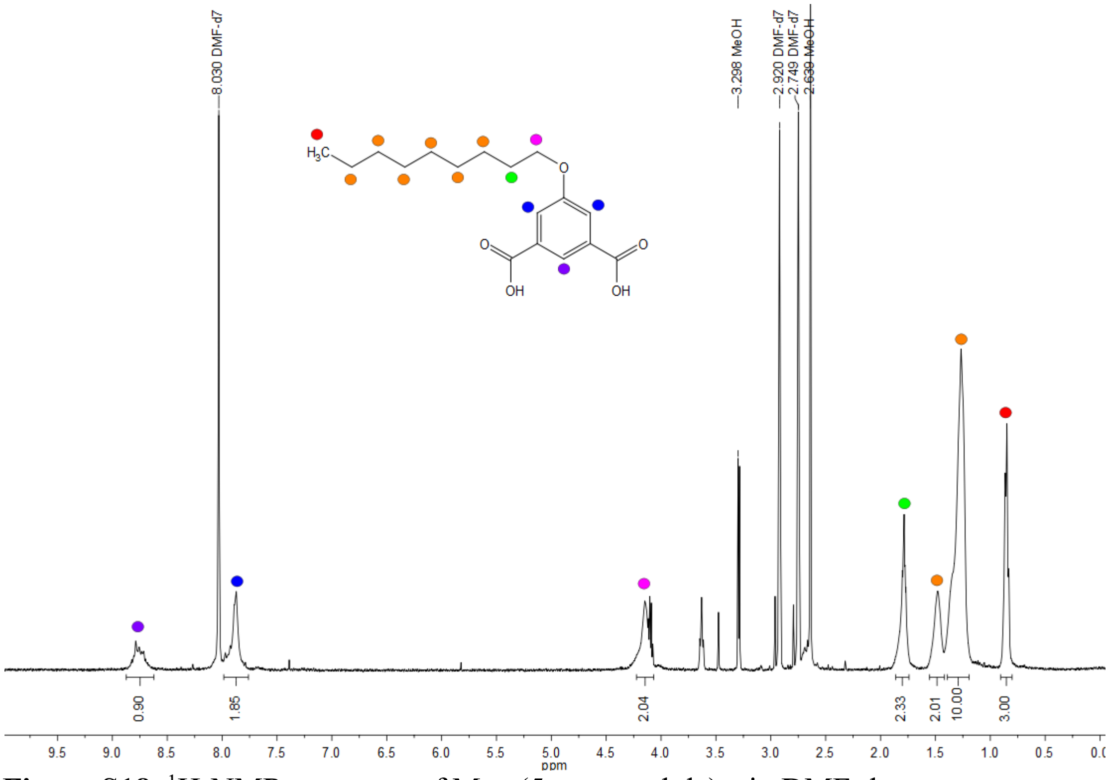

Figure S18. ^{1}H-NMR spectrum of Mo_{24}(5-nonoxy-bdc)$_{24}$ in DMF-d$_7$.

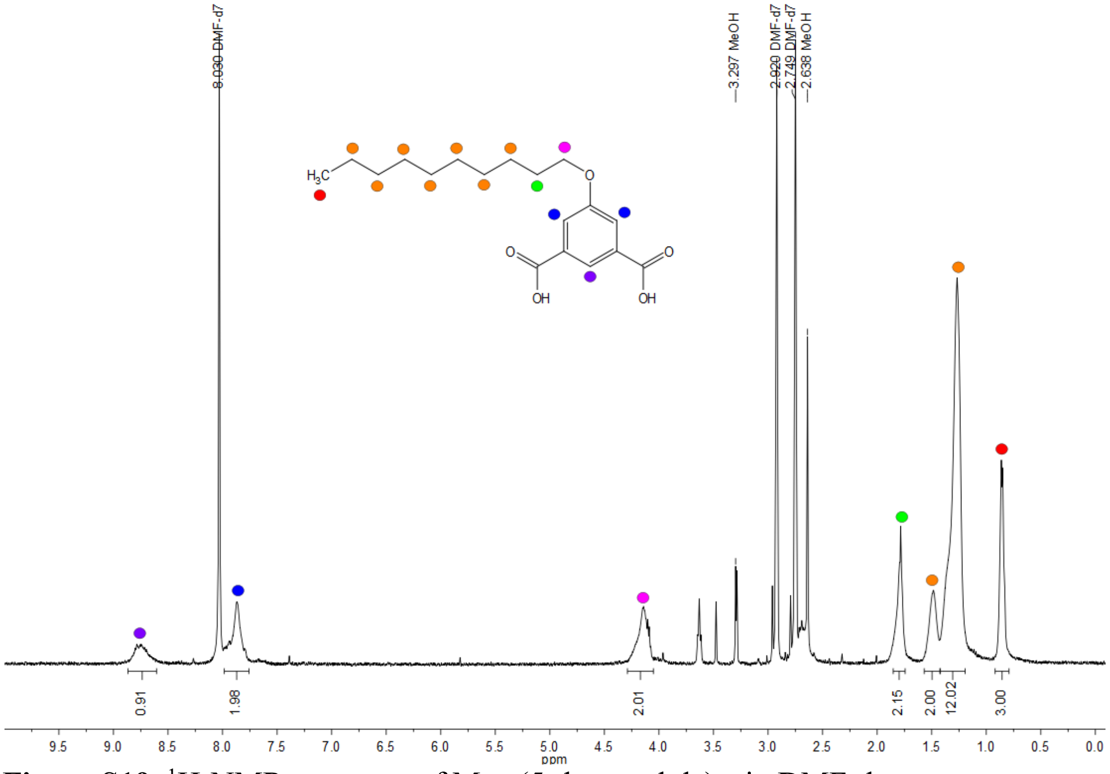

Figure S19. ^{1}H-NMR spectrum of Mo_{24}(5-decoxy-bdc)$_{24}$ in DMF-d$_7$.

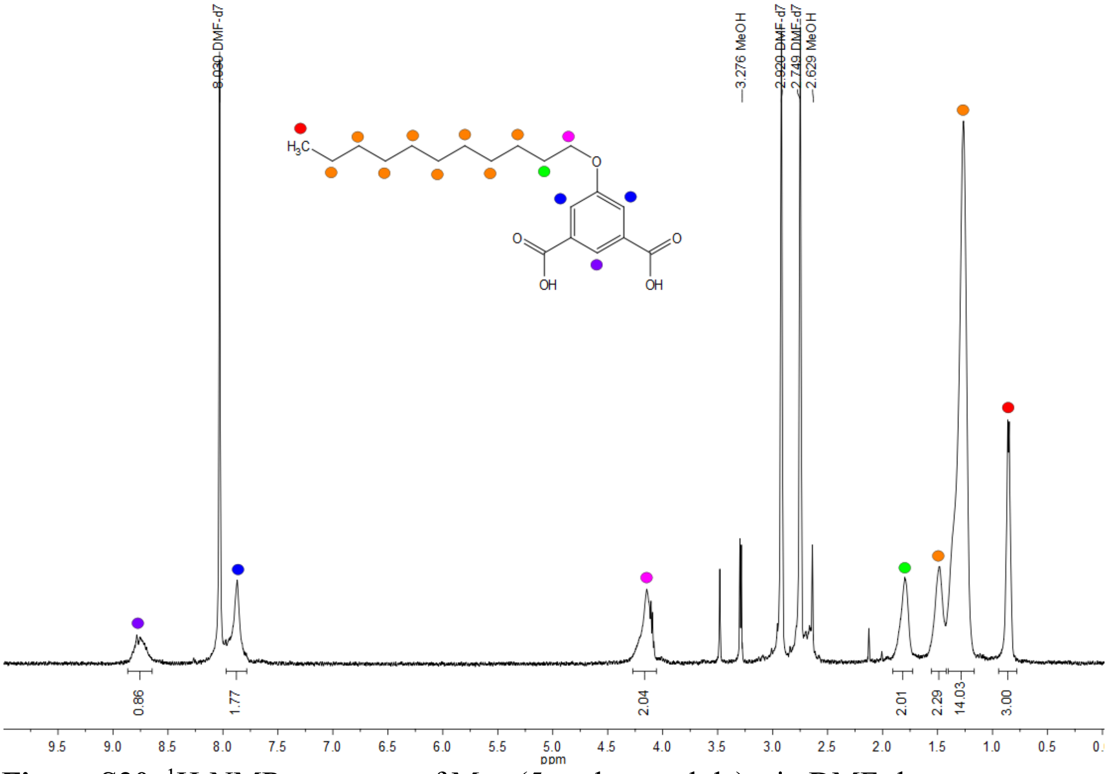

Figure S20. ^{1}H-NMR spectrum of Mo_{24}(5-undecoxy-bdc)$_{24}$ in DMF-d_7.

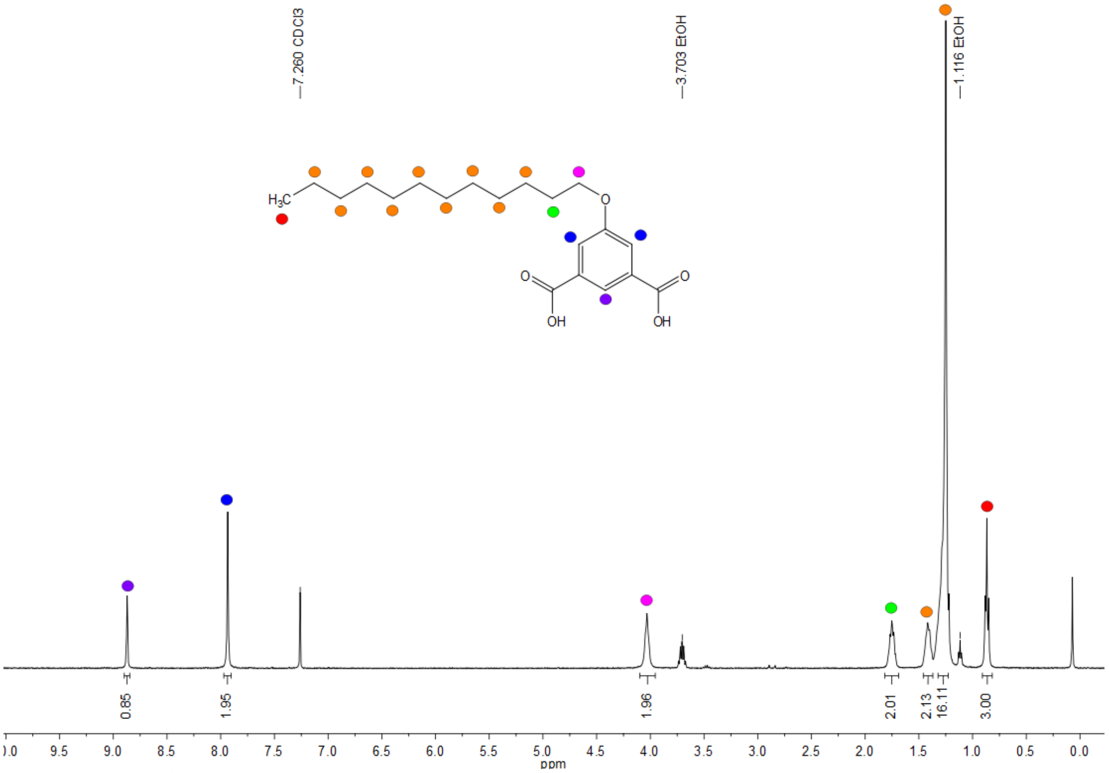

Figure S21. ^{1}H-NMR spectrum of Mo_{24}(5-dodecoxy-bdc)$_{24}$ in $CDCl_3$.

NMR Spectra of Mo(II) Ligand Exchange Controls

In these experiments, a Mo(II) alkoxy cage was combined with a different length alkoxy ligand in a 1:1 ratio and heated at 100 °C in DMF to produce a homogenous solution. For instance, 0.1 mmol of Mo_{24}(5-hexoxy-bdc)$_{24}$ was combined with 0.1 mmol of 5-nonoxy-1,3-benzenedicarboxylic acid in DMF. Upon addition of MeOH, a powder crashed out and was washed with MeOH three times. The samples were then dried under dynamic vacuum and dissolved in NMR solvent in order to determine if ligand exchange occurred.

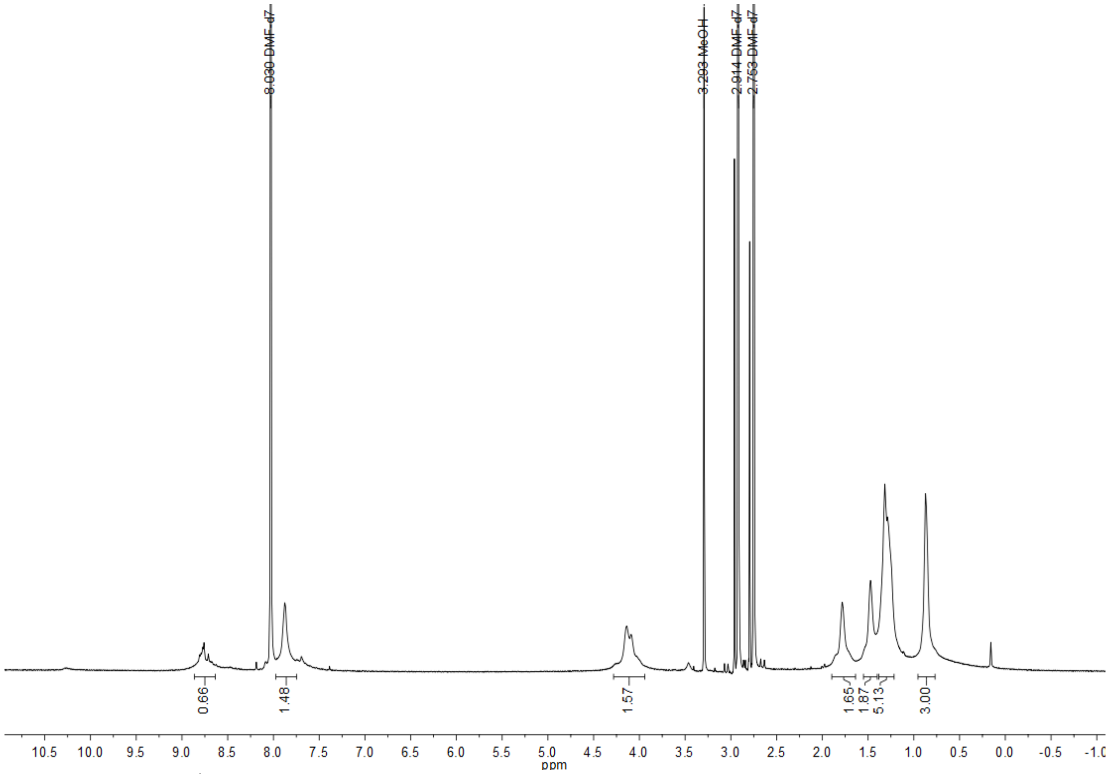

Figure S22. ^{1}H-NMR spectrum of Mo_{24}(5-hexoxy-bdc)$_{24}$ exchanged with 5-nonoxy-1,3-benzenedicarboxylic acid in DMF. The NMR was taken in DMF-d_7.

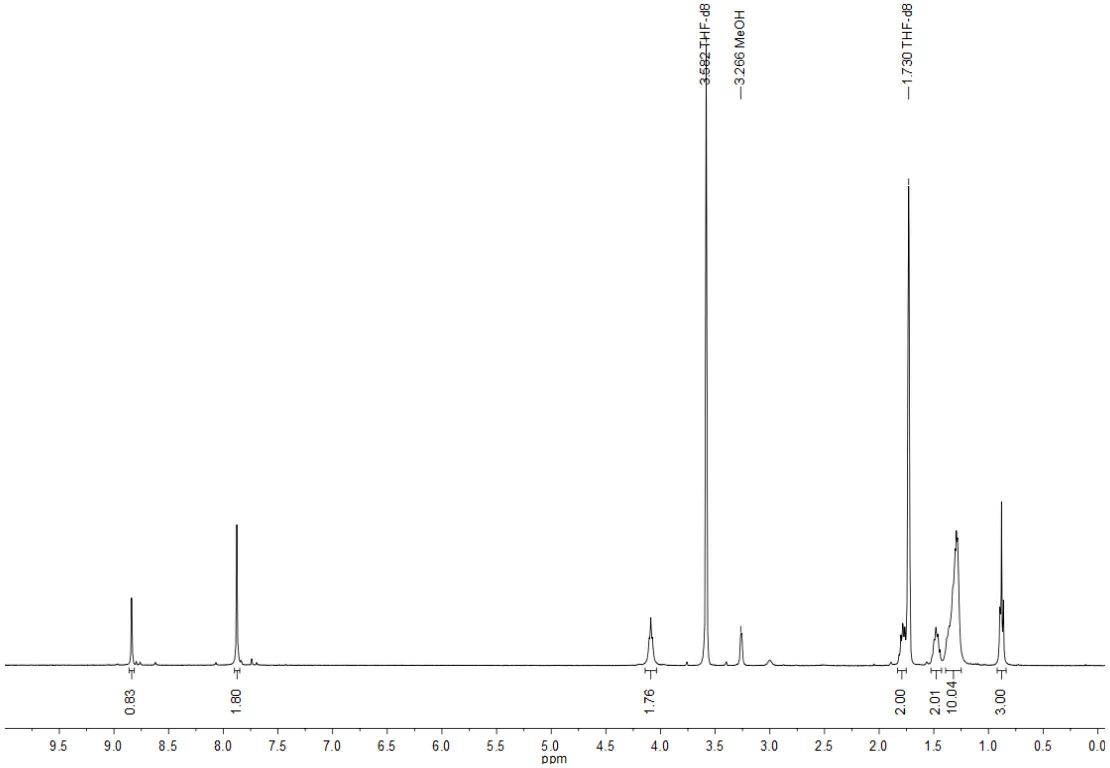

Figure S23. ^{1}H-NMR spectrum of Mo_{24}(5-nonoxy-bdc)$_{24}$ exchanged with 5-dodecoxy-1,3-benzenedicarboxylic acid in DMF. The NMR was taken in THF-d_8.

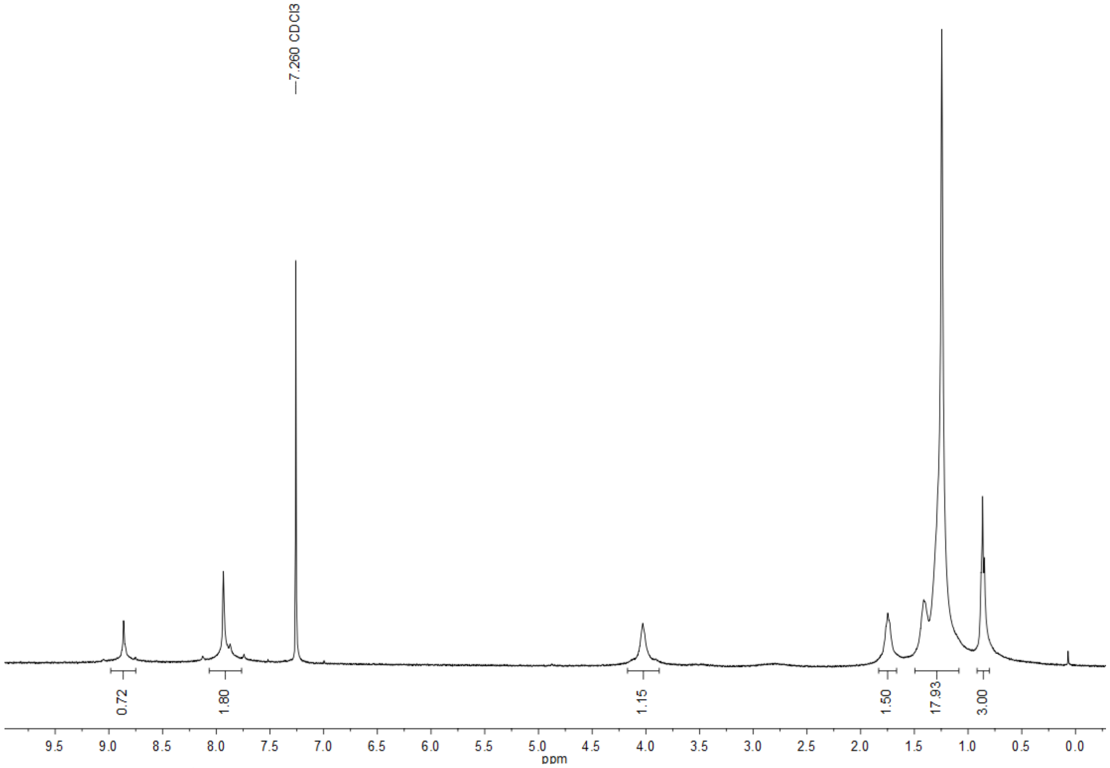

Figure S24. ^{1}H-NMR spectrum of Mo_{24}(5-dodecoxy-bdc)$_{24}$ exchanged with 5-hexoxy-1,3-benzenedicarboxylic acid in DMF. The NMR was taken in $CDCl_3$.

Characterization of Metal and Ligand Exchange Controls

In this experiment, 0.1 mmol of Cu(5-hexoxy-bdc)$_{24}$ was combined with 0.1 mmol Mo(5-nonoxy-bdc)$_{24}$ in DMF and heated to 100 °C to produce a homogenous solution. The Cu/Mo alloy powder was crashed out of solution using MeOH and washed three times with MeOH to remove residual DMF. After the powder was dried under dynamic vacuum, the Mo(5-nonoxy-bdc)$_{24}$ was extracted via THF and separated into another vial. The alloy powder was washed thrice with THF to remove residual Mo(5-nonoxy-bdc)$_{24}$ cage and the washed were combined. Methanol was used to crash out the Mo(5-nonoxy-bdc)$_{24}$ cage from the THF solution. The powder was washed three times with MeOH, dried under vacuum, and dissolved in NMR solvent in order to determine if ligand or metal exchange occurred. The isolated Cu(5-hexoxy-bdc)$_{24}$ was dried under dynamic vacuum for use in UV-Vis experiments to determine if ligand or metal exchange occurred.

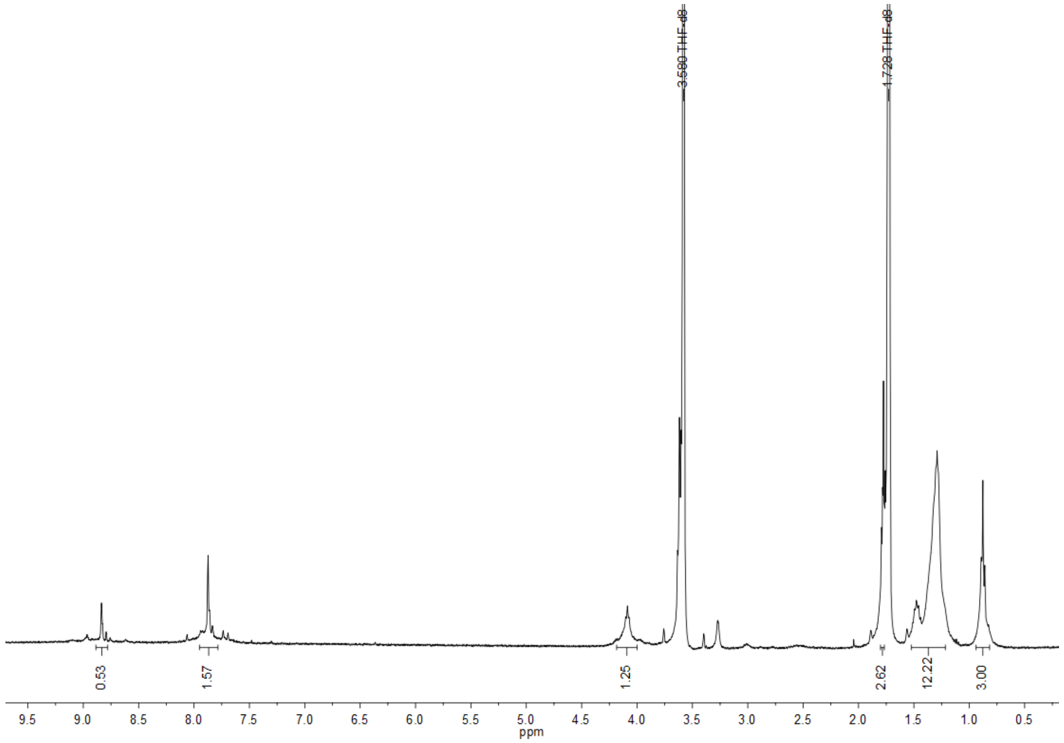

Figure S25. ^{1}H-NMR spectrum of Mo$_{24}$(5-nonoxy-bdc)$_{24}$ separated from Cu(5-hexoxy-bdc)$_{24}$ after isolation from DMF solution and THF extraction. The NMR was taken in THF-d_8.

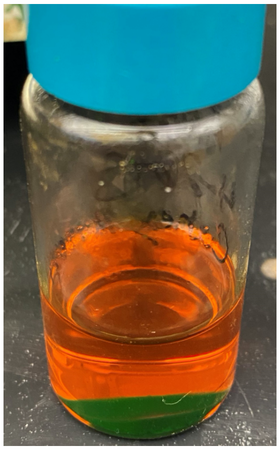

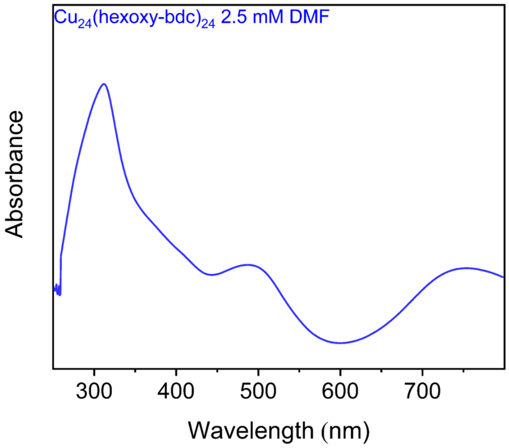

Figure S26. Image of the metal/ligand exchange control containing Cu_{24}(5-hexoxy-bdc)$_{24}$ and Mo_{24}(5-nonoxy-bdc)$_{24}$ after the addition of THF to the powder crashed out from DMF and washed with MeOH (left). UV-vis of a 2.5 mM DMF solution of the Cu_{24}(5-hexoxy-bdc)$_{24}$ cage (green precipitate) separated and dried from the Mo_{24}(5-nonoxy-bdc)$_{24}$ cage and vacuum dried.

Phase Transfer of CuMo Alloy for Cage Separation

A 1:1 ratio of Mo_{24}(5-hexoxy-bdc)$_{24}$ or Mo_{24}(5-nonoxy-bdc)$_{24}$ to Cu_{24}(5-dodecoxy-bdc)$_{24}$ and Cu_{24}(5-hexoxy-bdc)$_{24}$ or Cu_{24}(5-nonoxy-bdc)$_{24}$ to Mo_{24}(5-dodecoxy-bdc)$_{24}$ was added to a 20 mL scintillation vial and dissolved in DMF. Then hexane, heptane or octane was layered on top of the DMF. Upon shaking, the Mo_{24}(5-dodecoxy-bdc)$_{24}$ or Cu_{24}(5-dodecoxy-bdc)$_{24}$ was extracted from the DMF to the alkane layer as seen by the color change of the alkane solvent. For the hexane phase transfer, this layer was collected via pipette after extracting three times to remove residual cage. The hexane layer was then pumped down to dryness and the DMF layer was crashed out with MeOH, washed three times and dried via vacuum. For the octane phase transfer, as MeOH is not miscible with octane, the DMF layer was simply crashed out with MeOH, washed three times and dried via vacuum.

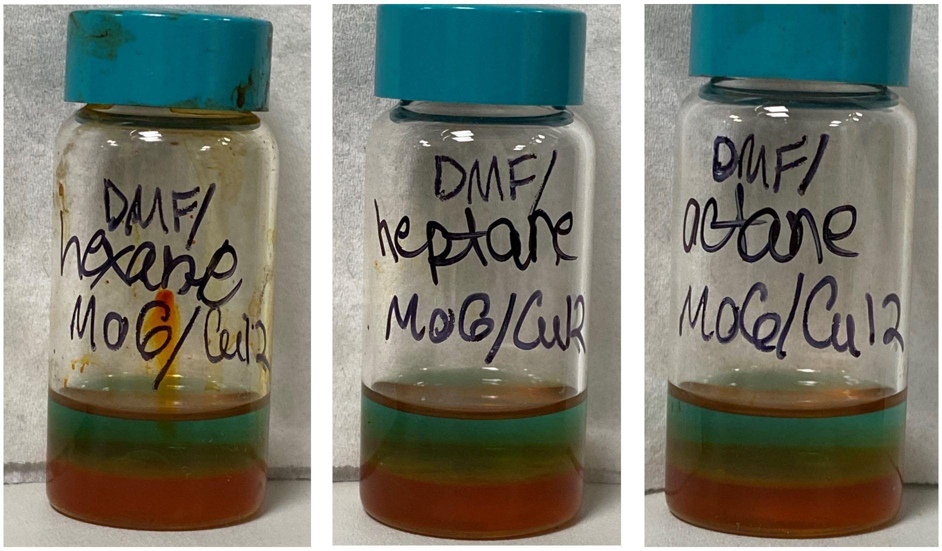

Figure S27. Images of Mo_{24}(5-hexoxy-bdc)$_{24}$/Cu_{24}(5-dodecoxy-bdc)$_{24}$ extractions using hexane (left), heptane (middle) and octane (right) after being shaken for a few seconds and allowed to settle.

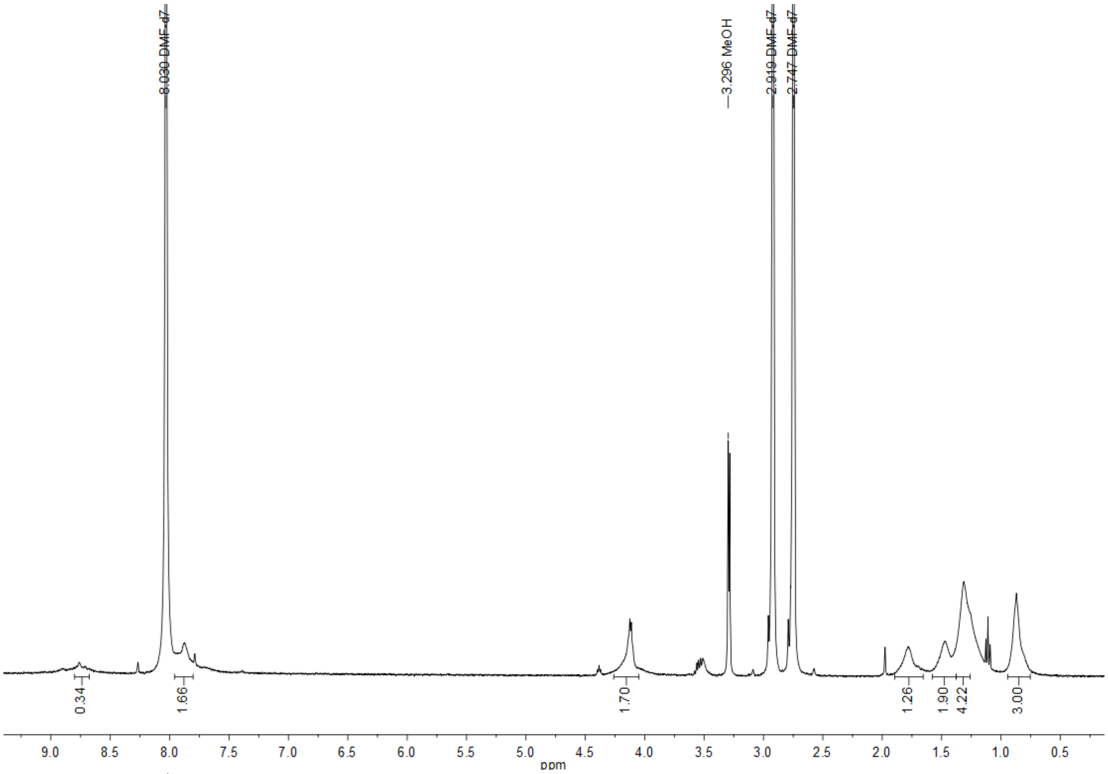

Figure S28. ^{1}H-NMR spectrum of Mo_{24}(5-hexoxy-bdc)$_{24}$ post phase transfer with Cu(5-dodecoxy-bdc)$_{24}$ in DMF/hexane. The DMF solution was extracted with hexane and then the Mo_{24}(5-hexoxy-bdc)$_{24}$ cage was crashed out of DMF, washed with MeOH three times and dried under vacuum. The NMR was taken in DMF-d_7.

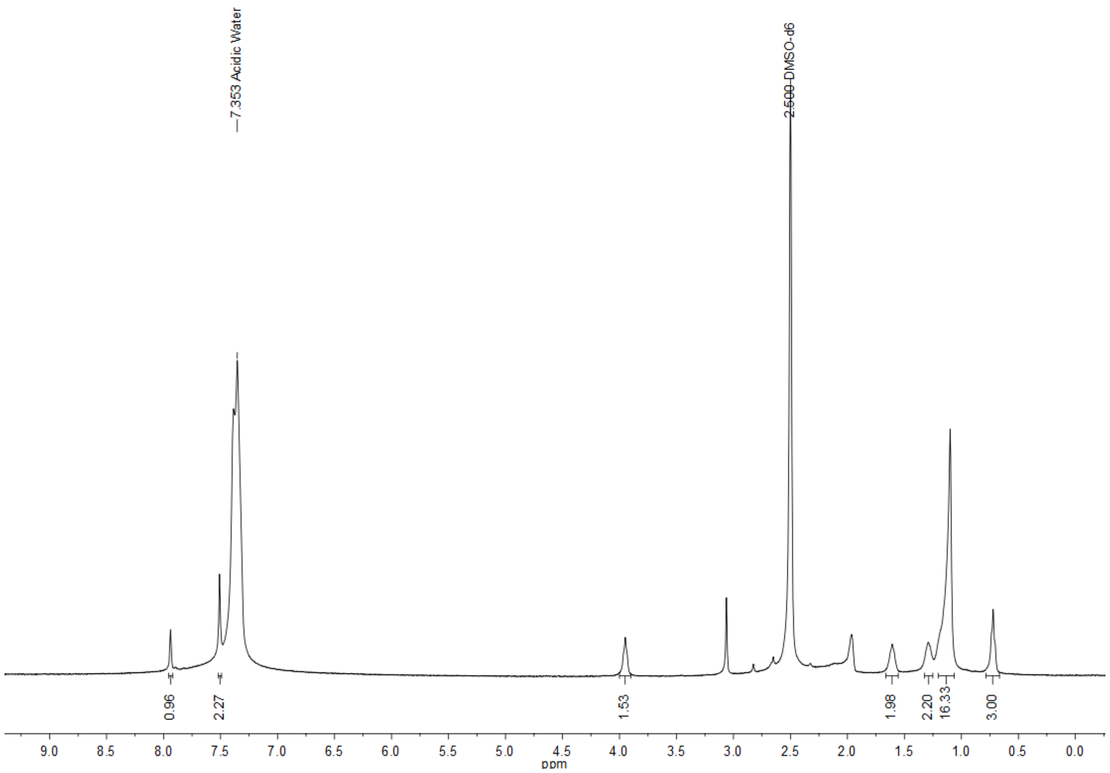

Figure S29. ^{1}H-NMR spectrum of digested Cu(5-dodecoxy-bdc)$_{24}$ post phase transfer with Mo$_{24}$(5-hexoxy-bdc)$_{24}$. The DMF solution was extracted with hexane. The Cu(5-dodecoxy-bdc)$_{24}$ hexane solution was separated into a new vial, pumped down until ~2 mL hexane remained and crashed out with MeOH. The powder was washed with MeOH three times and dried under vacuum. The NMR was taken in DMSO after digestion of the cage with 2 drops of DCl.

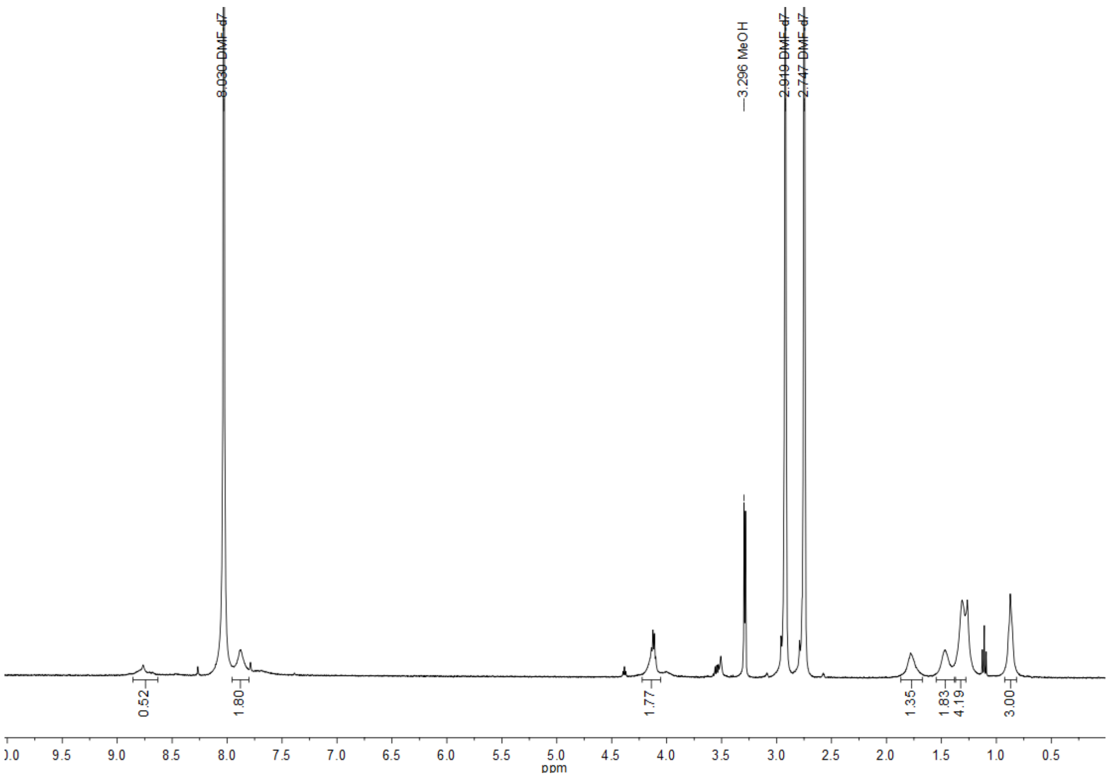

Figure S30. ^{1}H-NMR spectrum of Mo_{24}(5-hexoxy-bdc)$_{24}$ post phase transfer with Cu(5-dodecoxy-bdc)$_{24}$ in DMF/octane. The DMF solution was extracted with octane and then the Mo_{24}(5-hexoxy-bdc)$_{24}$ cage was crashed out of DMF, washed with MeOH three times and dried under vacuum. The NMR was taken in DMF-d_7.

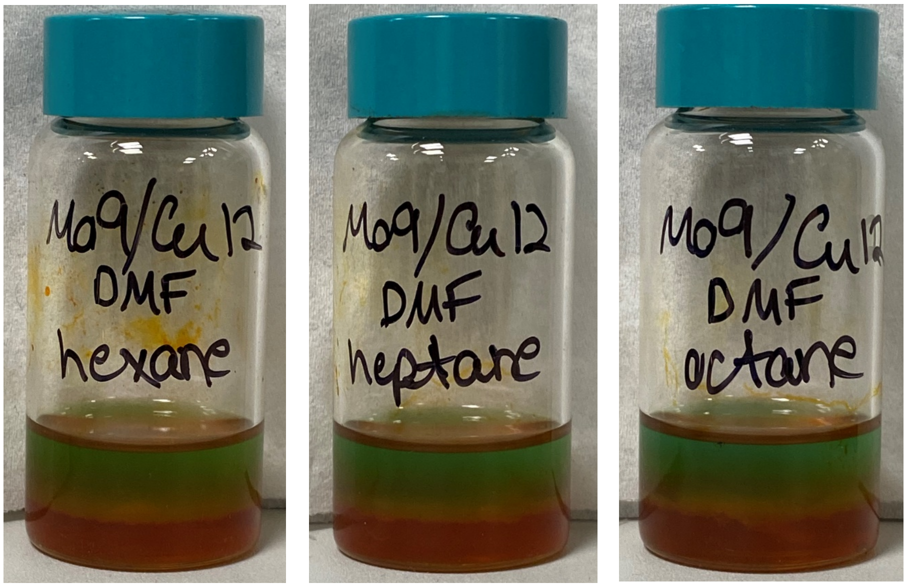

Figure S31. Images of Mo_{24}(5-nonoxy-bdc)$_{24}$/Cu_{24}(5-dodecoxy-bdc)$_{24}$ extractions using hexane (left), heptane (middle) and octane (right) after being shaken for a few seconds and allowed to settle.

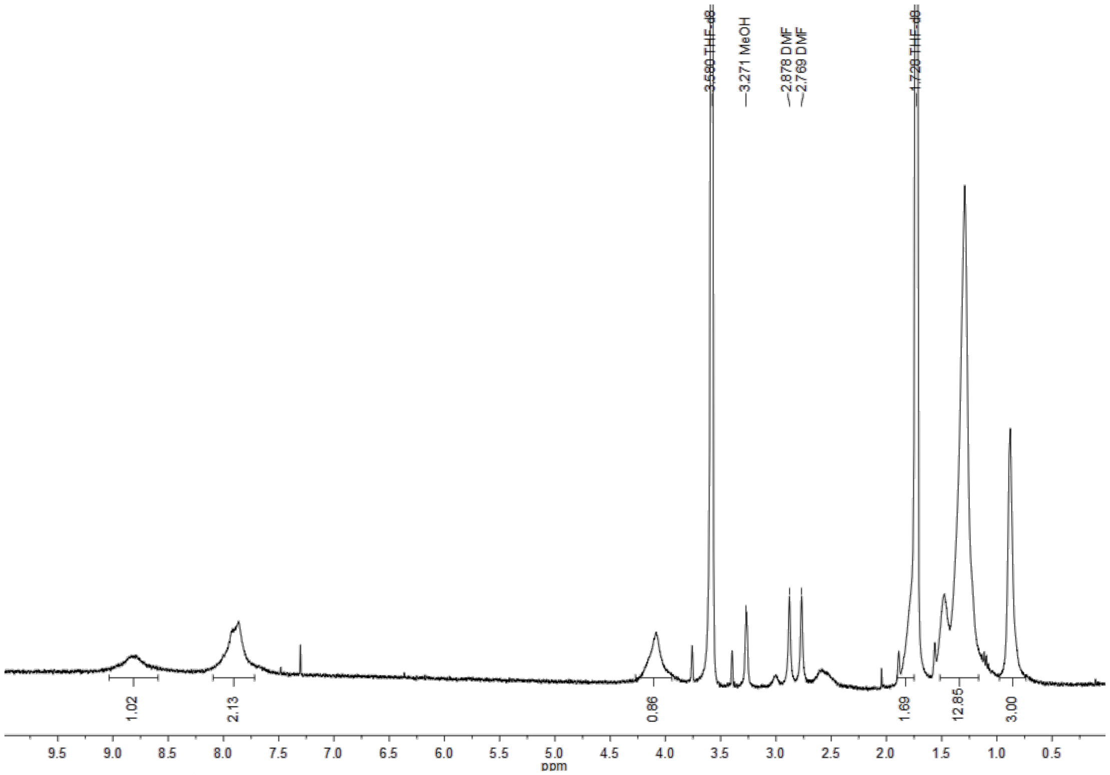

Figure S32. ^{1}H-NMR spectrum of Mo_{24}(5-nonoxy-bdc)$_{24}$ post phase transfer with Cu(5-dodecoxy-bdc)$_{24}$ in DMF/hexane. The DMF solution was extracted with hexane and then the Mo_{24}(5-nonoxy-bdc)$_{24}$ cage was crashed out of DMF, washed with MeOH three times and dried under vacuum. The NMR was taken in THF-d_8.

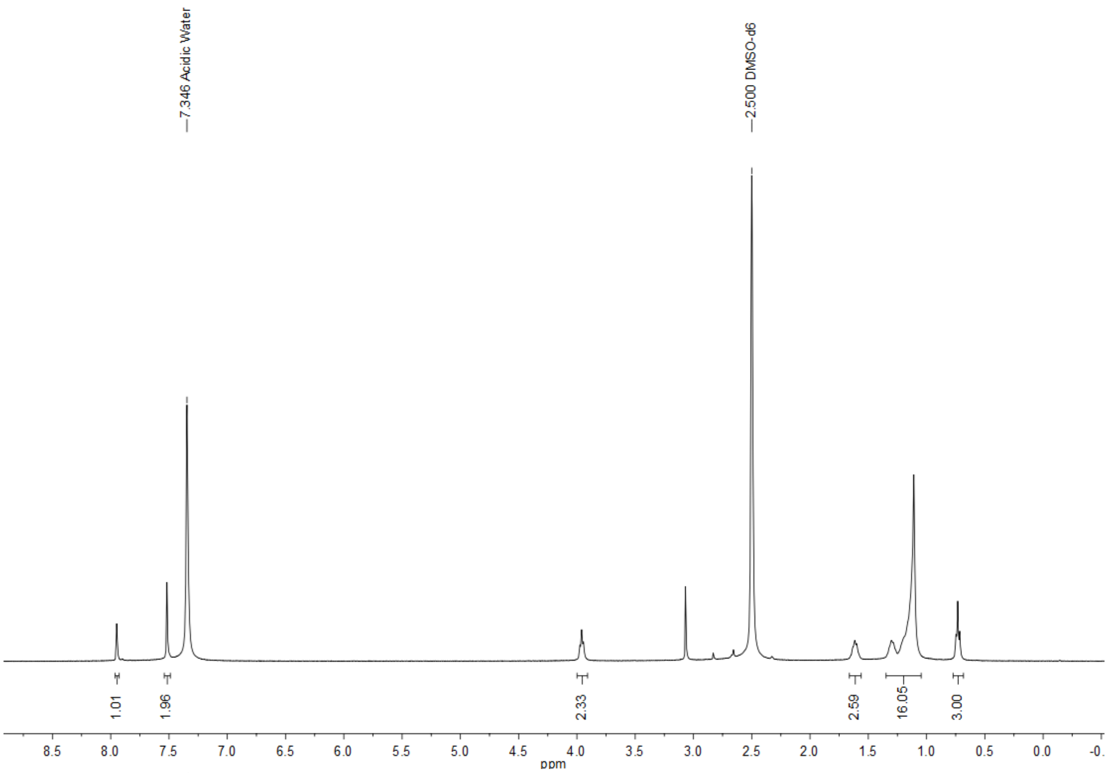

Figure S33. ^{1}H-NMR spectrum of digested Cu(5-dodecoxy-bdc)$_{24}$ post phase transfer with Mo$_{24}$(5-nonoxy-bdc)$_{24}$. The DMF solution was extracted with hexane. The Cu(5-dodecoxy-bdc)$_{24}$ hexane solution was separated into a new vial, pumped down until ~2 mL hexane remained and crashed out with MeOH. The powder was washed with MeOH three times and dried under vacuum. The NMR was taken in DMSO after digestion of the cage with 2 drops of DCl.

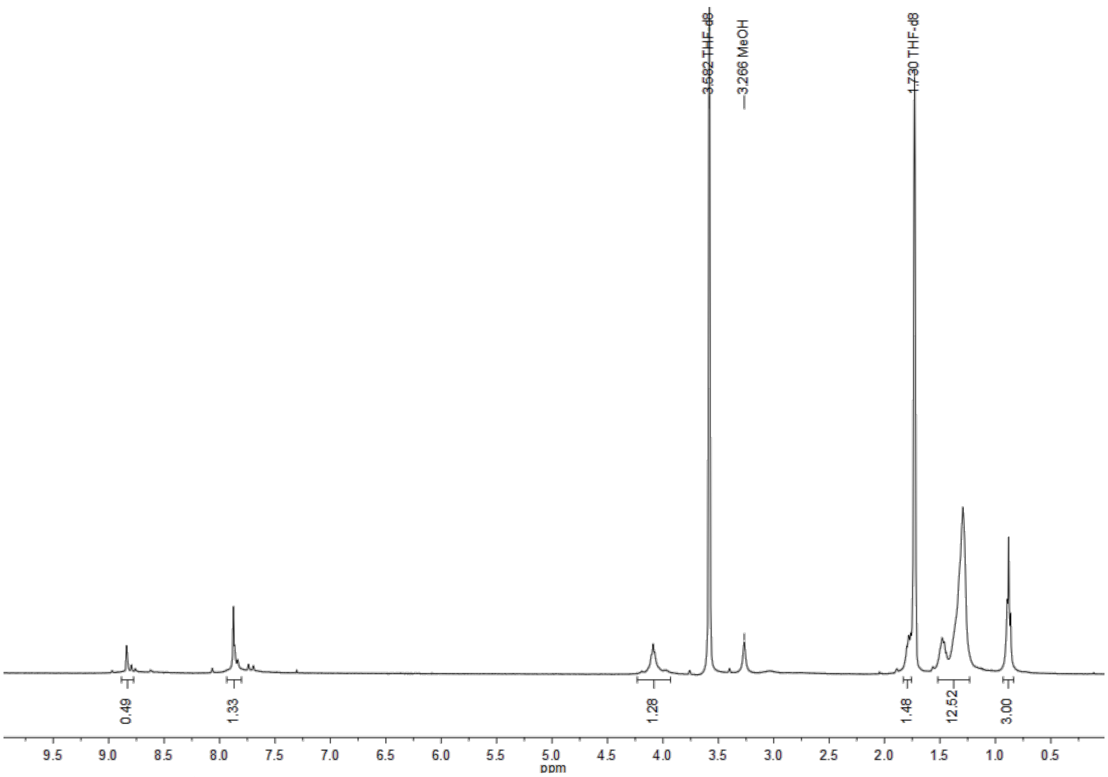

Figure S34. ^{1}H-NMR spectrum of Mo_{24}(5-nonoxy-bdc)$_{24}$ post phase transfer with Cu(5-dodecoxy-bdc)$_{24}$ in DMF/octane. The DMF solution was extracted with octane and then the Mo_{24}(5-nonoxy-bdc)$_{24}$ cage was crashed out of DMF, washed with MeOH three times and dried under vacuum. The NMR was taken in THF-d_8.

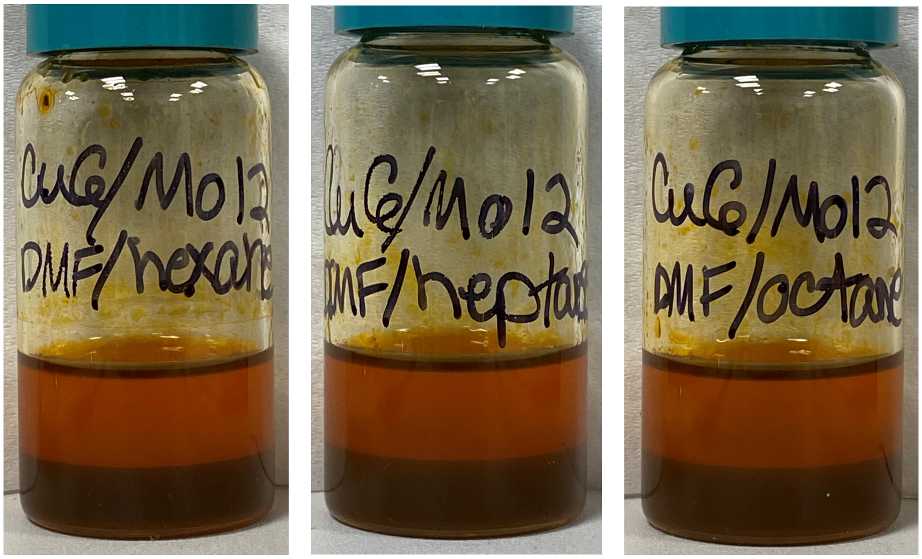

Figure S35. Images of Cu_{24}(5-hexoxy-bdc)$_{24}$/Mo_{24}(5-dodecoxy-bdc)$_{24}$ extractions using hexane (left), heptane (middle) and octane (right) after being shaken for a few seconds and allowed to settle.

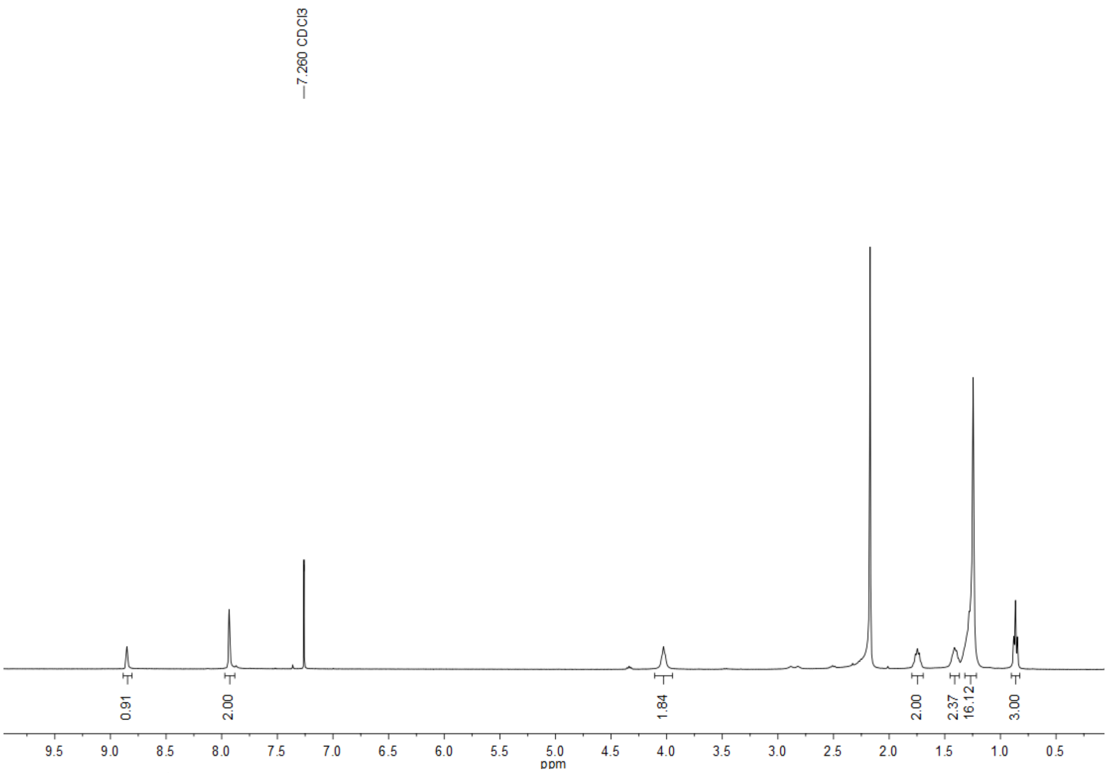

Figure S36. ^{1}H-NMR spectrum of Mo_{24}(5-dodecoxy-bdc)$_{24}$ post phase transfer with Cu(5-hexoxy-bdc)$_{24}$ in DMF/hexane. The DMF solution was extracted with hexane three times or until the hexane layer was clear. This hexane layer containing Mo_{24}(5-dodecoxy-bdc)$_{24}$ was then vacuum dried, dissolved in benzene and then crashed out with MeOH. The powder was washed three times with MeOH and dried under vacuum. The NMR was taken in $CDCl_3$.

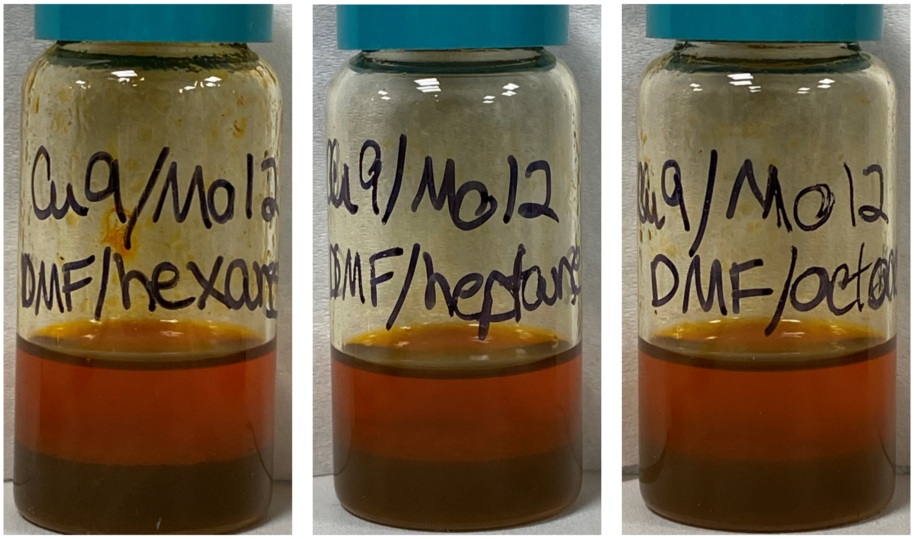

Figure S37. Images of Cu_{24}(5-nonoxy-bdc)$_{24}$/Mo_{24}(5-dodecoxy-bdc)$_{24}$ extractions using hexane (left), heptane (middle) and octane (right) after being shaken for a few seconds and allowed to settle.

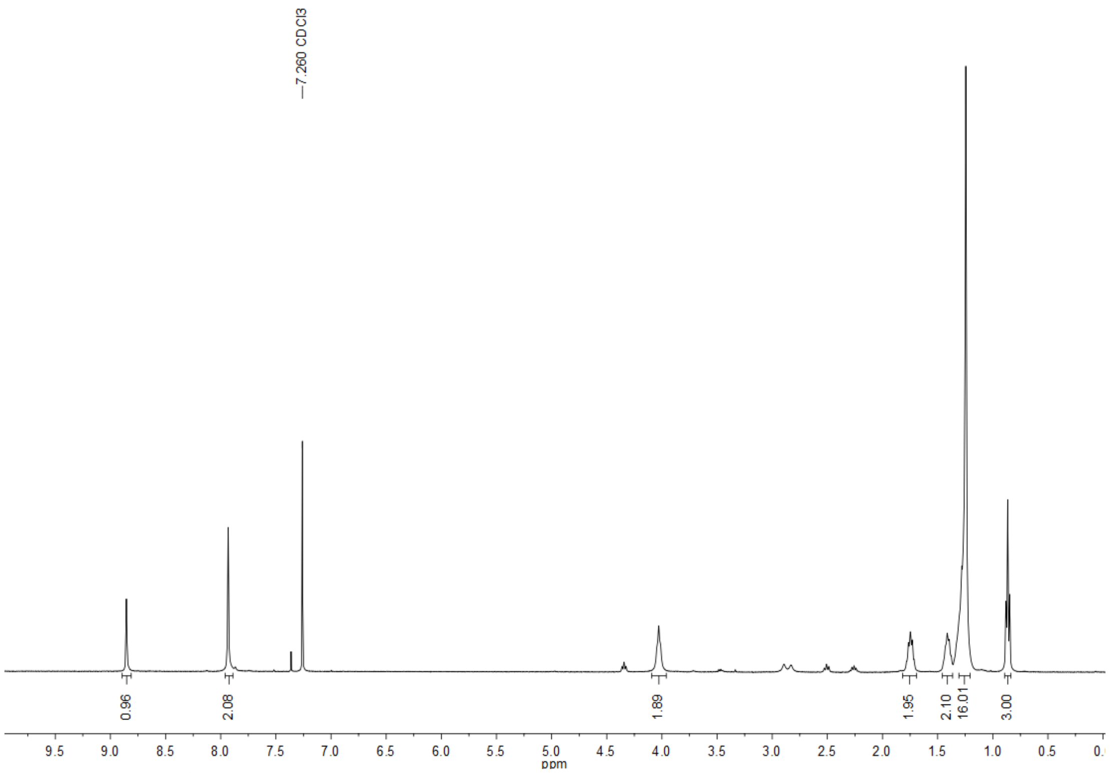

Figure S38. ^{1}H-NMR spectrum of Mo_{24}(5-dodecoxy-bdc)$_{24}$ post phase transfer with Cu(5-nonoxy-bdc)$_{24}$ in DMF/hexane. The DMF solution was extracted with hexane three times or until the hexane layer was clear. This hexane layer containing Mo_{24}(5-dodecoxy-bdc)$_{24}$ was then vacuum dried, dissolved in benzene and then crashed out with MeOH. The powder was washed three times with MeOH and dried under vacuum. The NMR was taken in $CDCl_3$.

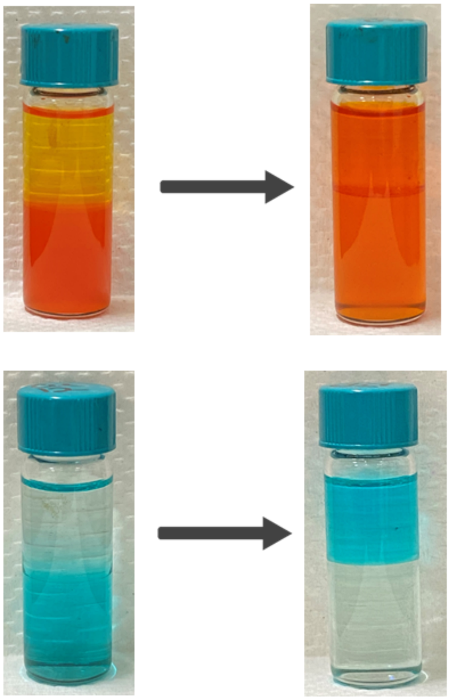

Figure S39. Images of Mo_{24}(5-dodecoxy-bdc)$_{24}$ dissolved in DMF upon hexane layering (top left) versus after being shaken for a few seconds and allowed to settle (top right). Images of Cu_{24}(5-dodecoxy-bdc)$_{24}$ dissolved in DMF upon hexane layering (bottom left) versus after being shaken for a few seconds and allowed to settle (bottom right).

X-Ray Crystallography:

X-ray structural analysis for Cu_{24}(5-hexoxy-bdc)$_{24}$, Cu_{24}(5-nonoxy-bdc)$_{24}$, and Cu_{24}(5-decoxy-bdc)$_{24}$. Crystal data and refinement details are shown in Table S1. Crystals were mounted using viscous oil onto a plastic mesh and cooled to the data collection temperature. Data were collected on a Bruker-AXS APEX II DUO CCD diffractometer with Cu-Kα radiation (λ = 1.54178 Å) focused with Goebel mirrors. Unit cell parameters were obtained from 48 data frames, 0.5º ω, from different sections of the Ewald sphere. The unit-cell dimensions, equivalent reflections and systematic absences in the diffraction data are uniquely consistent with C2/m, C2 and Cm. Refinement in the centrosymmetric space group options, C2/m, yielded chemically reasonable and computationally stable results of refinement. The data were treated with multi-scan absorption corrections.[2] Structures were solved using intrinsic phasing methods[3] and refined with full-matrix, least-squares procedures on F2.[4]

Residual electron density, solvent molecules and atoms that cannot be assigned a reasonable model, were treated as diffused electron density using Squeeze.[5] The ligand alkyl chains were confirmed by digestion followed by NMR spectroscopy and thus the electron density that was deducted from the diffused alkyl chain ends could be assigned. Although the assignments of void contents were based on the best spatial and electron count fit of the reaction solvent DMF and water, we cannot discount the possibility of alternative void contents that might consist of thermal decomposition products of amide solvents. The compound molecules were located at an intersection of a mirror plane and a two-fold rotation axis. A coordinated DMF molecule was found disordered in two positions with a 53/47 refined site occupancy ratio.

These compounds consistently deposit as weakly diffracting crystals and the results herein represent the best of several trials. In order to compensate for the low-resolution data, various constraints and restraints were applied. All non-hydrogen atoms were refined with anisotropic displacement parameters. All H-atoms were treated as idealized contributions with geometrically calculated positions and with U_{iso} equal to 1.2-1.5 U_{eq} of the attached carbon atom. Atomic scattering factors are contained in the SHELXTL program library.[4] The structures have been deposited at the Cambridge Structural Database under the following CCDC deposition numbers: 2018425-2018427.

Table S1. Crystal data summary and structure refinement details.

	Cu_{24}(5-hexoxy-bdc)$_{24}$	Cu_{24}(5-nonoxy-bdc)$_{24}$	Cu_{24}(5-decoxy-bdc)$_{24}$
Sum Formula*	$C_{428}H_{552}Cu_{24}N_{24}O_{144}$	$C_{434}H_{616}Cu_{24}N_{8}O_{144}$	$C_{504}H_{744}Cu_{24}N_{24}O_{144}$*
Moiety Formula*	$C_{264}H_{184}Cu_{24}N_{4}O_{144}$, 8[$C_6H_{13}$], 8[$C_5H_{11}$], 4[$C_4H_9$], 20[$C_3H_7N$]	$C_{254}H_{168}Cu_{24}N_{8}O_{144}$, 6[$C_9H_{19}$], 6[$C_8H_{19}$], 8[$C_6H_{15}$], 4[$C_7H_{17}$], 16[$H_2O$]	$C_{224}H_{116}Cu_{24}N_{4}O_{144}$, 12[$C_{10}H_{21}$], 4[$C_9H_{21}$], 8[$C_8H_{19}$], 20[$C_3H_7N$]
Temperature (K)	150	150	150
Crystal system	monoclinic	monoclinic	monoclinic
Space group	*C*2/*m*	*C*2/*m*	*C*2/*m*
Formula weight *M* (g/mol)*	9861.86	9750.25	10968.15
Unit cell dimensions			
a (Å)	34.0993(15)	38.1865(15)	38.162(2)
b (Å)	34.2455(15)	30.1168(12)	30.3222(17)
c (Å)	23.9647(11)	26.1698(11)	28.8145(17)
β (°)	113.073(2)	106.633(3)	110.311(3)
V (Å^3)	25746(2)	28837(2)	31269(3)
Occupancy (*Z*)	2	2	2
Reflections collected / unique / R_{int}	84550 / 13747 / 0.0753	128531/ 21271 / 0.0996	100219 / 12594 / 0.0618
Final *R* indices [I>2σ] R_1 / wR_2	0.0870 / 0.2348	0.0978 / 0.2819	0.0816 / 0.2454
R indices (all data) R_1 / wR_2	0.1244 / 0.2950	0.1380 / 0.3340	0.1074 / 0.2855
CCDC	2018425	2018426	2018427

*These data-sets were processed with Squeeze to account for diffused electron density in areas of the difference map that cannot be modelled as chemically reasonable, computationally convergent, moieties. The moieties in square brackets are based on the best fit of the known ligand alkyl chain, majority solvent (DMF) and water.

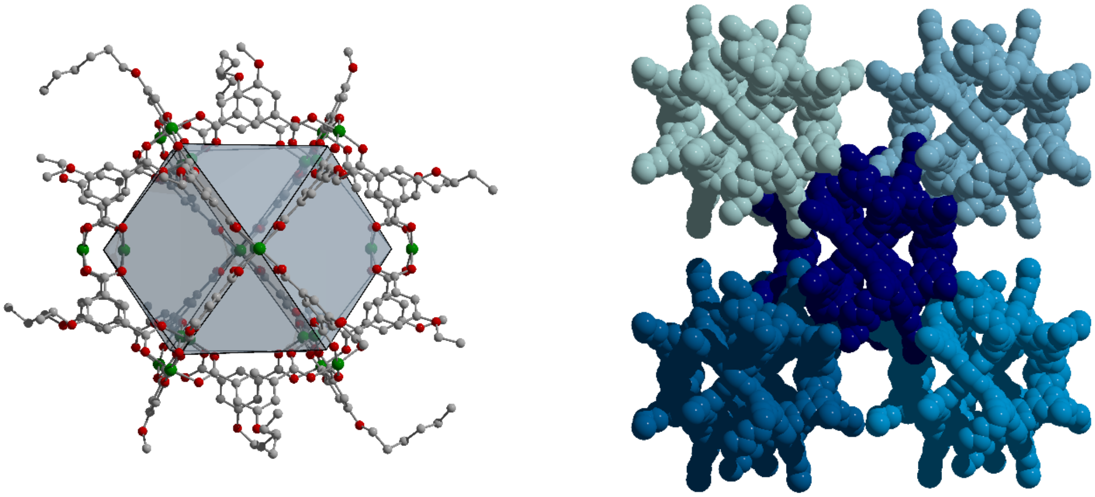

Figure S40. X-ray crystal structure of Cu_{24}(5-hexoxy-bdc)$_{24}$ (Left). Due to weak diffraction at wider angles, some of the ligand functional groups could not be fully modeled. Crystallographic packing of Cu_{24}(5-hexoxy-bdc)$_{24}$ viewing down the C axis (Right).

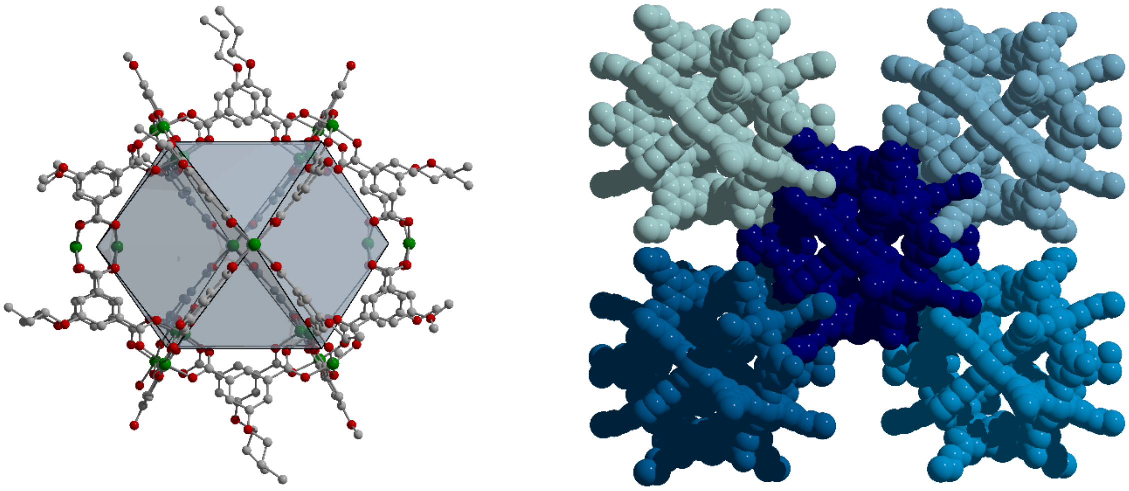

Figure S41. X-ray crystal structure of Cu_{24}(5-nonoxy-bdc)$_{24}$ (Left). Due to weak diffraction at wider angles, ligand functional groups could not be fully modeled. Crystallographic packing of Cu_{24}(5-nonoxy-bdc)$_{24}$ viewing down the C axis (Right).

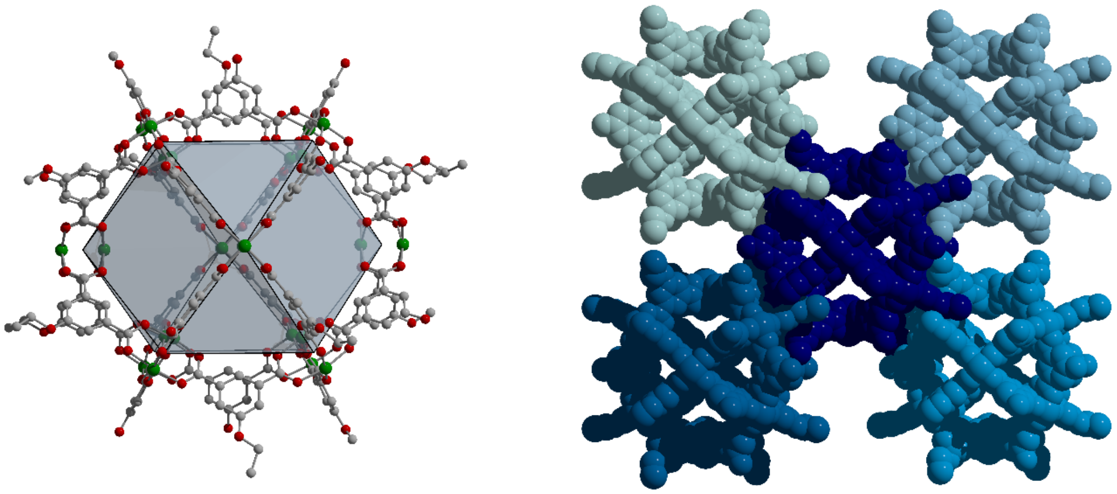

Figure S42. X-ray crystal structure of Cu_{24}(5-decoxy-bdc)$_{24}$ (Left). Due to weak diffraction at wider angles, ligand functional groups could not be fully modeled. Crystallographic packing of Cu_{24}(5-decoxy-bdc)$_{24}$ viewing down the C axis (Right).

Powder X-Ray Diffraction:

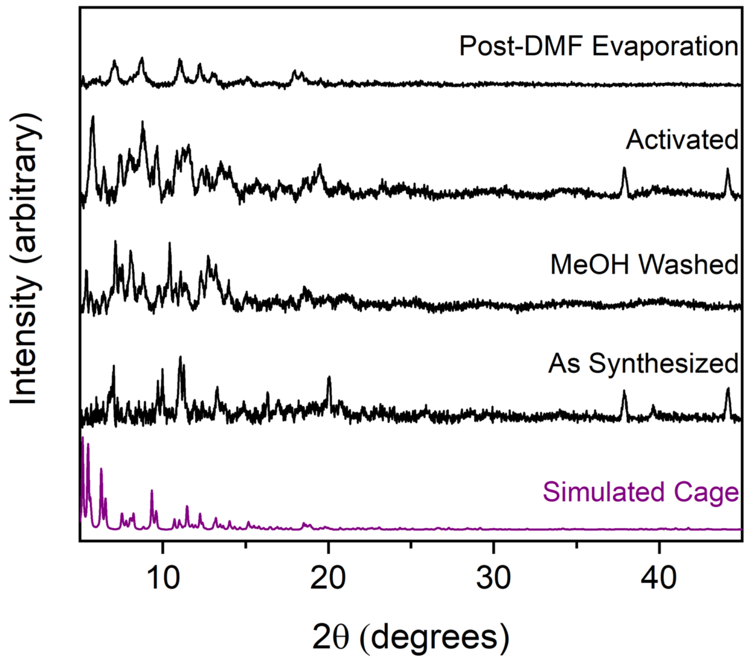

Figure S43. PXRD of Cu_{24}(5-hexoxy-bdc)$_{24}$ cage. To obtain Post-DMF Evaporation PXRDs, the methanol washed cage was dissolved in DMF and subsequently reduced under vacuum then activated at its optimal temperature.

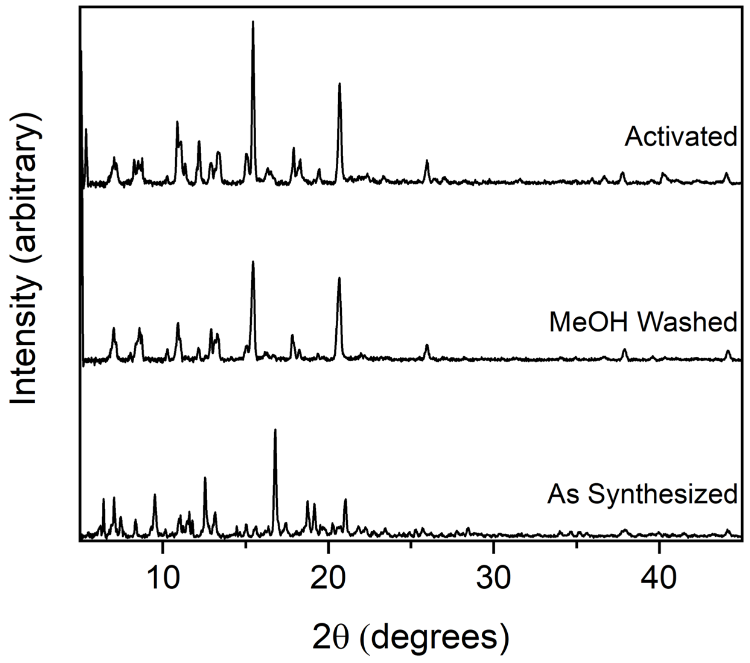

Figure S44. PXRD of Cu_{24}(5-heptoxy-bdc)$_{24}$ cage.

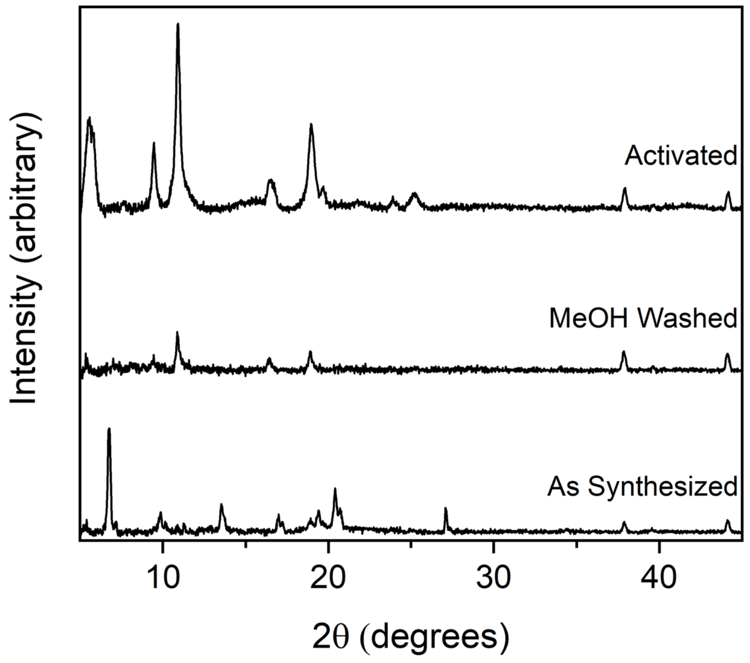

Figure S45. PXRD of Cu_{24}(5-octoxy-bdc)$_{24}$ cage.

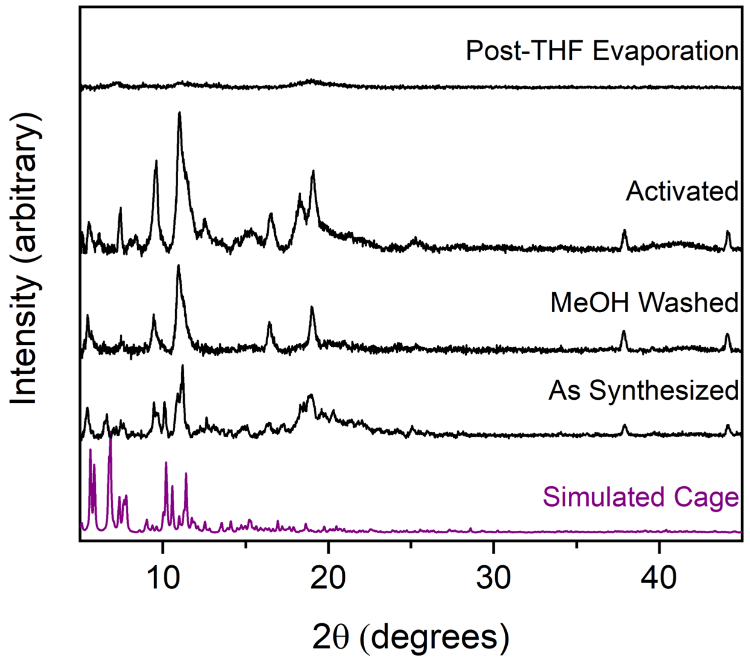

Figure S46. PXRD of Cu_{24}(5-nonoxy-bdc)$_{24}$ cage. To obtain Post-THF Evaporation PXRDs, the methanol washed cage was dissolved in THF and subsequently reduced under vacuum then activated at its optimal temperature.

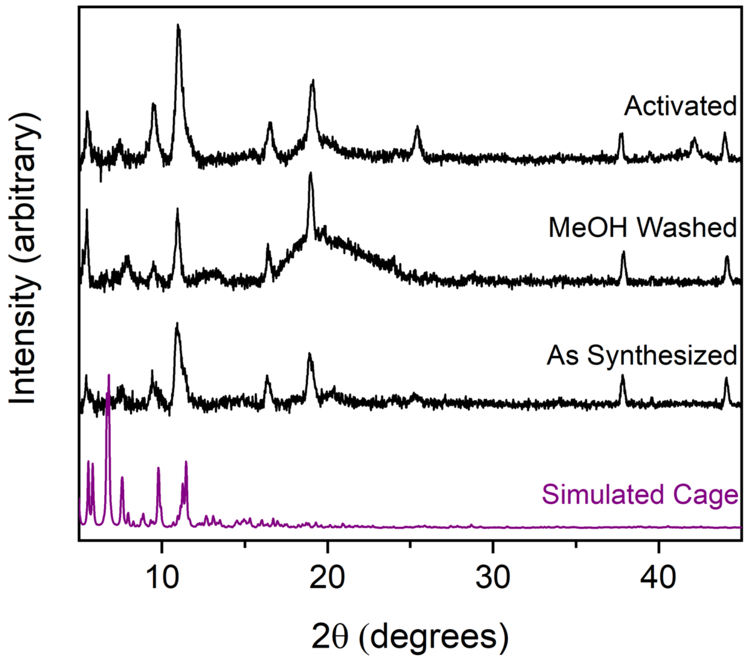

Figure S47. PXRD of Cu_{24}(5-decoxy-bdc)$_{24}$ cage.

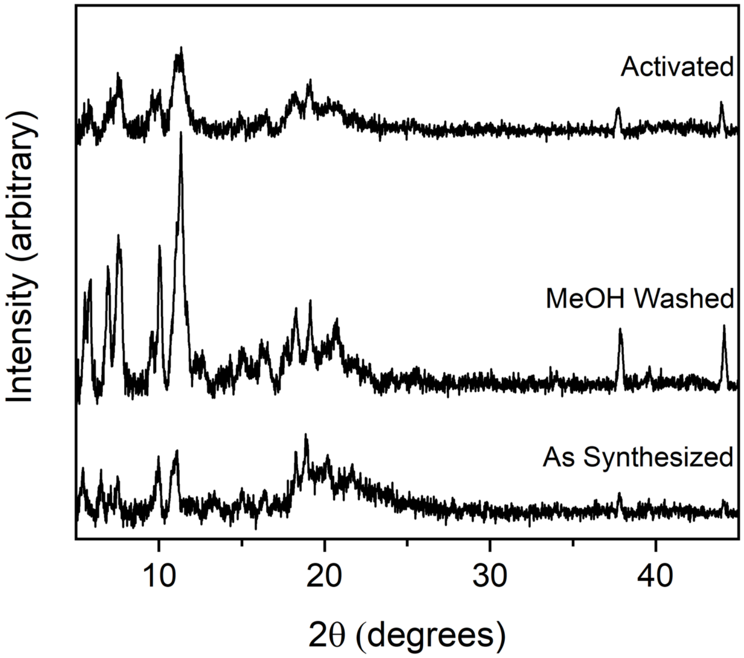

Figure S48. PXRD of Cu_{24}(5-undecoxy-bdc)$_{24}$ cage.

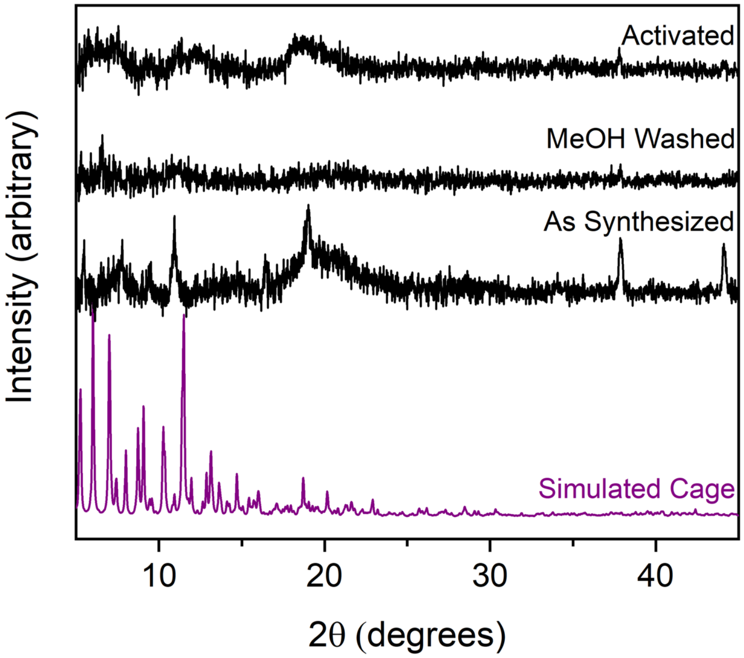

Figure S49. PXRD of Cu_{24}(5-dodecoxy-bdc)$_{24}$ cage.

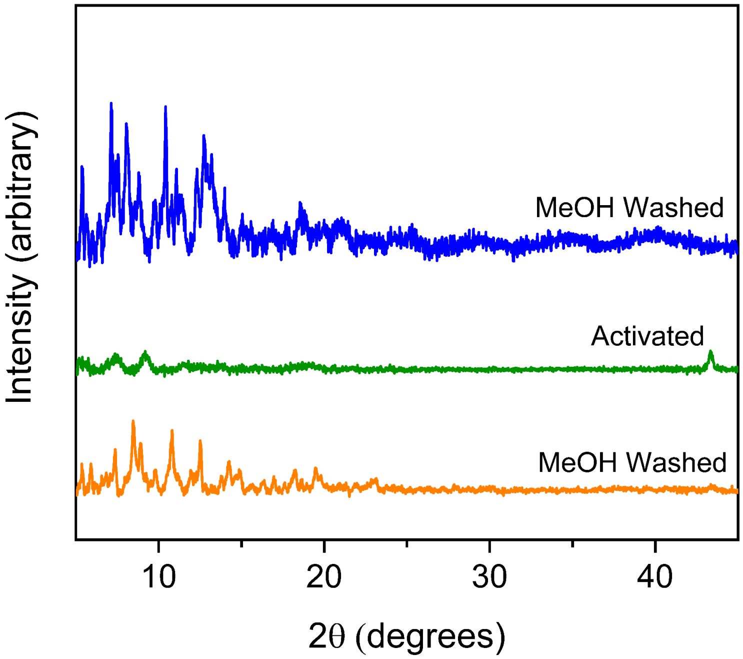

Figure S50. PXRDs of Mo_{24}(5-hexoxy-bdc)$_{24}$ cage (orange), CuMo(5-hexoxy-bdc) alloy (green) and Cu_{24}(5-hexoxy-bdc)$_{24}$ cage (blue).

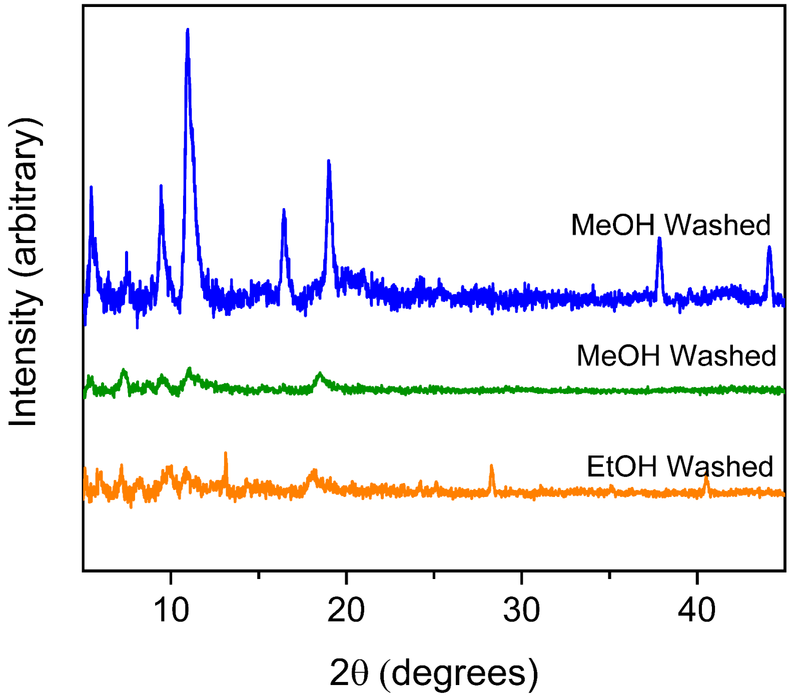

Figure S51. PXRDs of Mo_{24}(5-nonoxy-bdc)$_{24}$ cage (orange), CuMo(5-nonoxy-bdc) alloy (green) and Cu_{24}(5-nonoxy-bdc)$_{24}$ cage (blue).

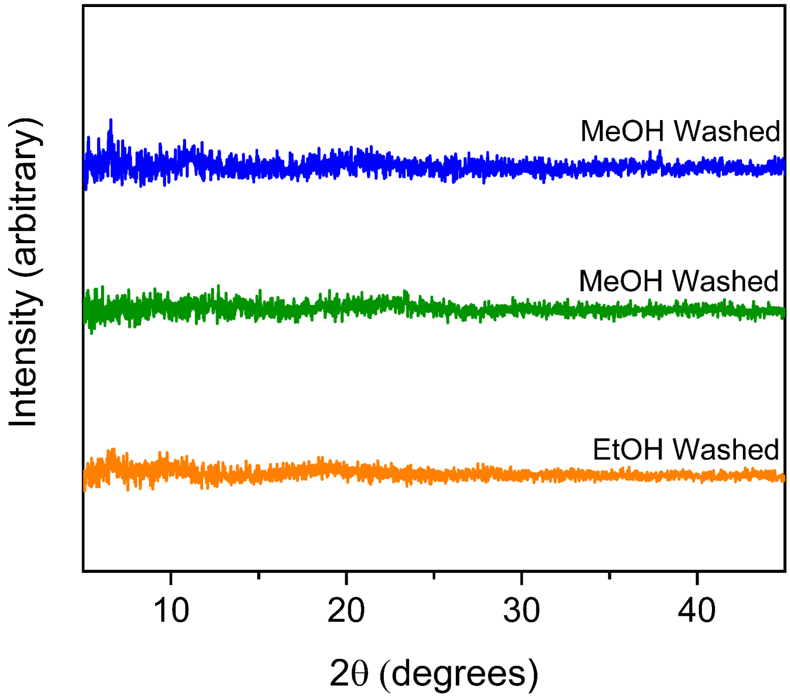

Figure S52. PXRDs of Mo_{24}(5-dodecoxy-bdc)$_{24}$ cage (orange), CuMo(5-dodecoxy-bdc) alloy (green) and Cu_{24}(5-dodecoxy-bdc)$_{24}$ cage (blue).

UV-Vis Spectra:

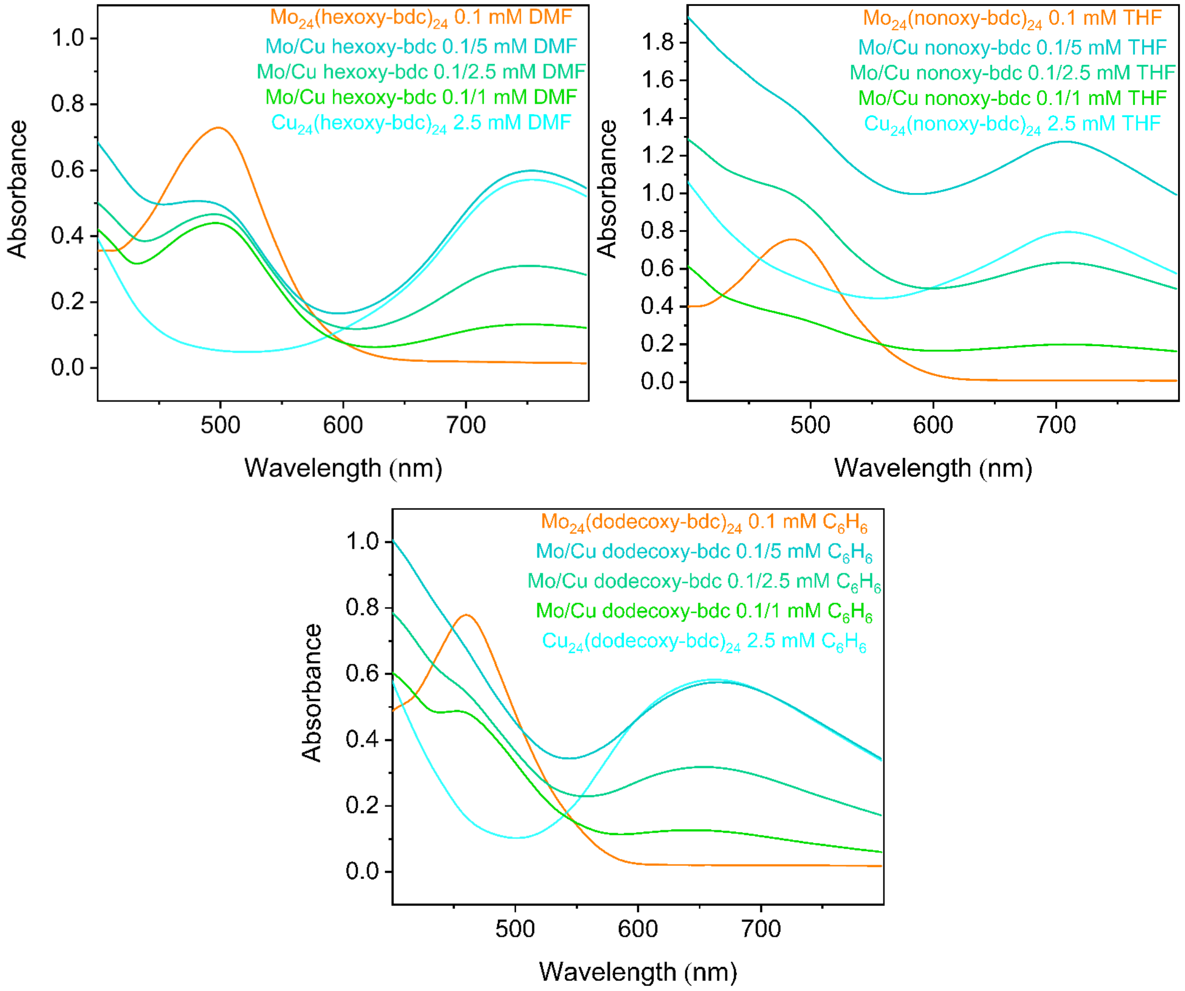

Figure S53. UV-Vis spectra for Mo(II) and Cu(II) cages as compared to the combined Mo/Cu cages at appropriate concentrations to compare the materials.

Infrared Spectra:

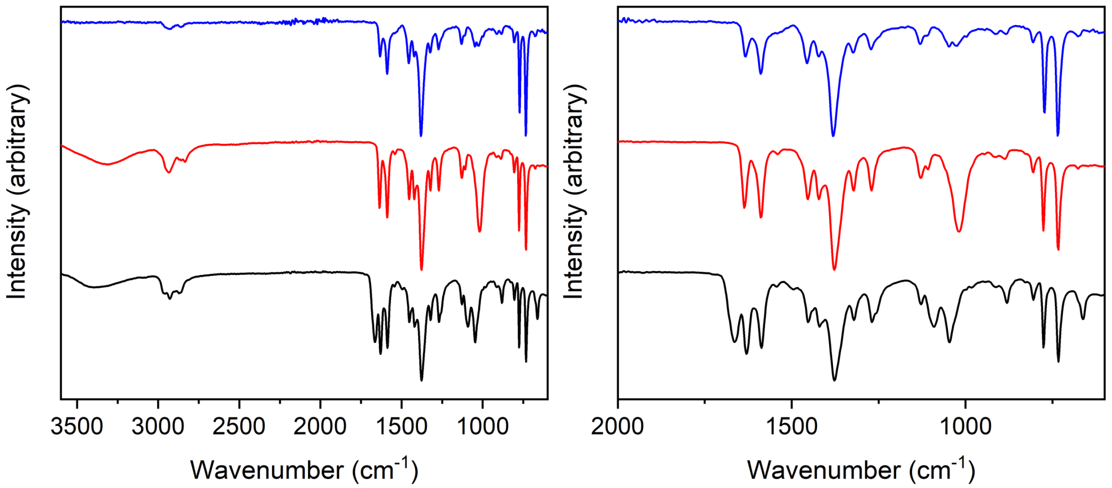

Figure S54. Infrared spectrum of Cu_{24}(5-hexoxy-bdc)$_{24}$ cage. Black: As synthesized material, Red: Methanol washed material, Blue: activated material.

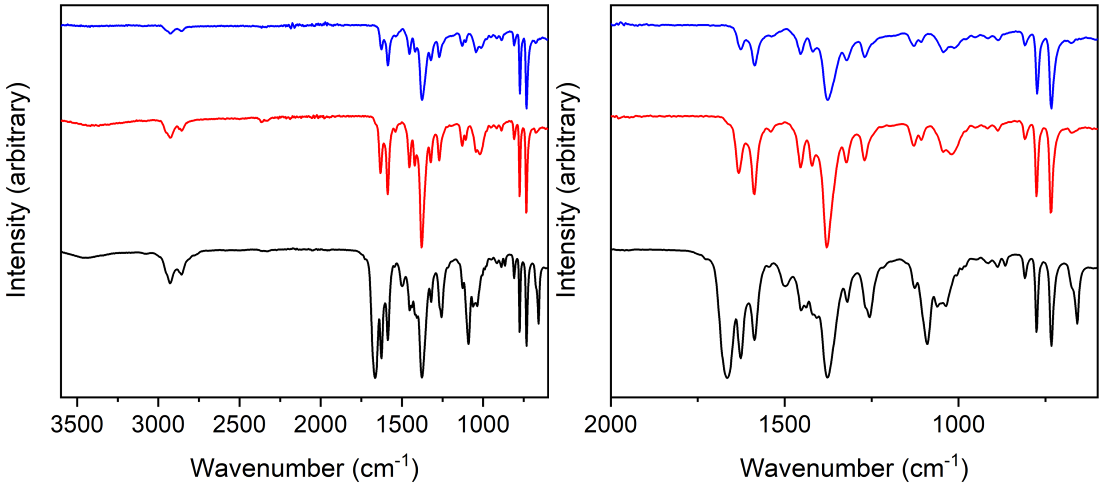

Figure S55. Infrared spectrum of Cu_{24}(5-heptoxy-bdc)$_{24}$ cage. Black: As synthesized material, Red: Methanol washed material, Blue: activated material.

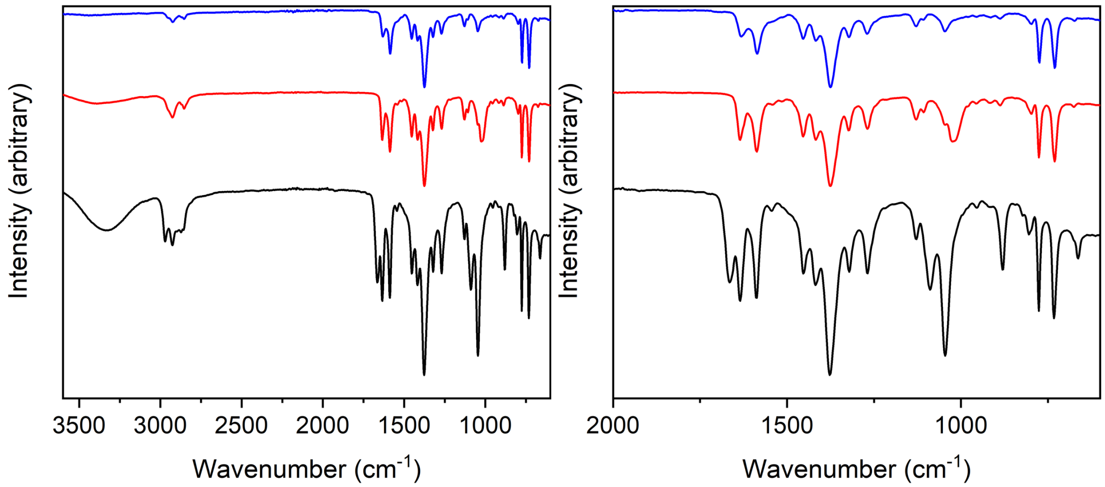

Figure S56. Infrared spectrum of Cu_{24}(5-octoxy-bdc)$_{24}$ cage. Black: As synthesized material, Red: Methanol washed material, Blue: activated material.

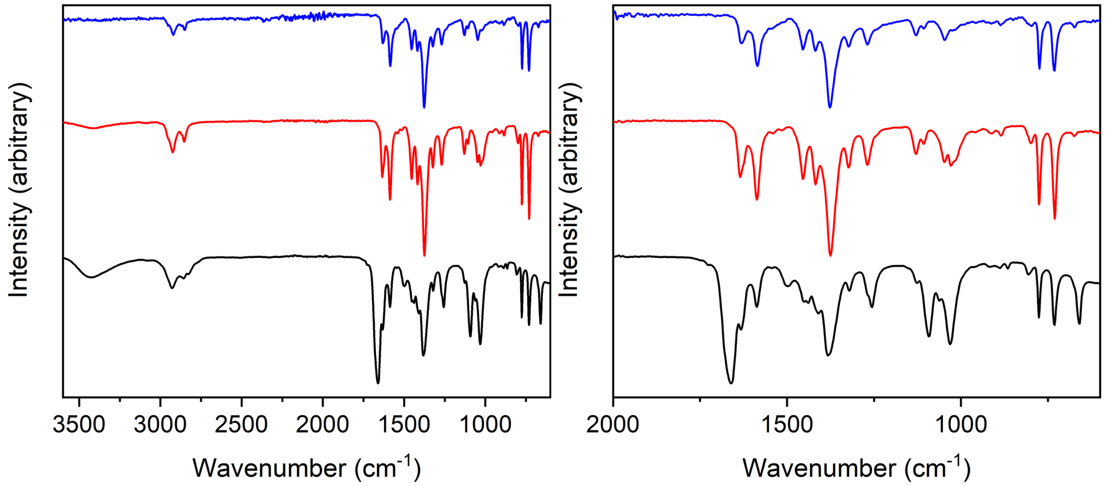

Figure S57. Infrared spectrum of Cu_{24}(5-nonoxy-bdc)$_{24}$ cage. Black: As synthesized material, Red: Methanol washed material, Blue: activated material.

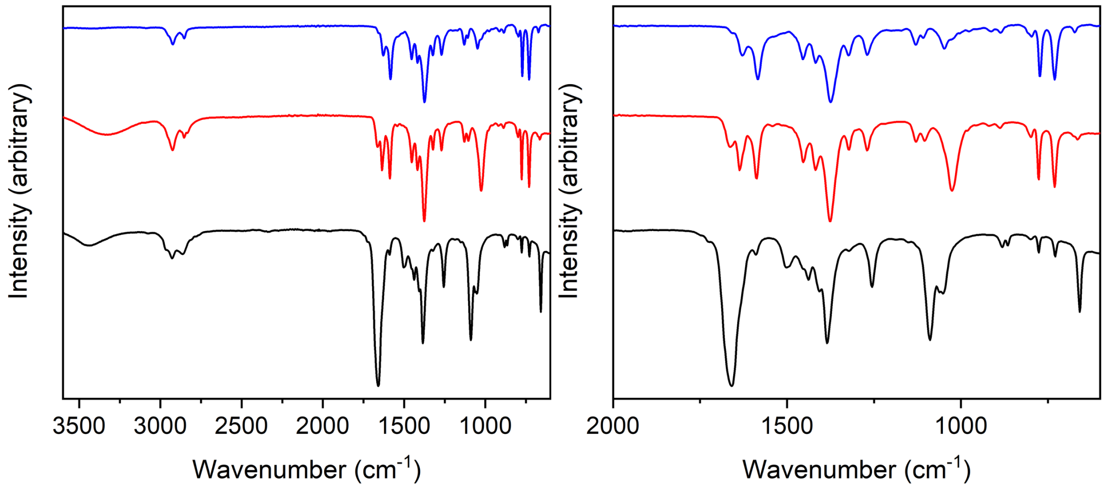

Figure S58. Infrared spectrum of Cu_{24}(5-decoxy-bdc)$_{24}$ cage. Black: As synthesized material, Red: Methanol washed material, Blue: activated material.

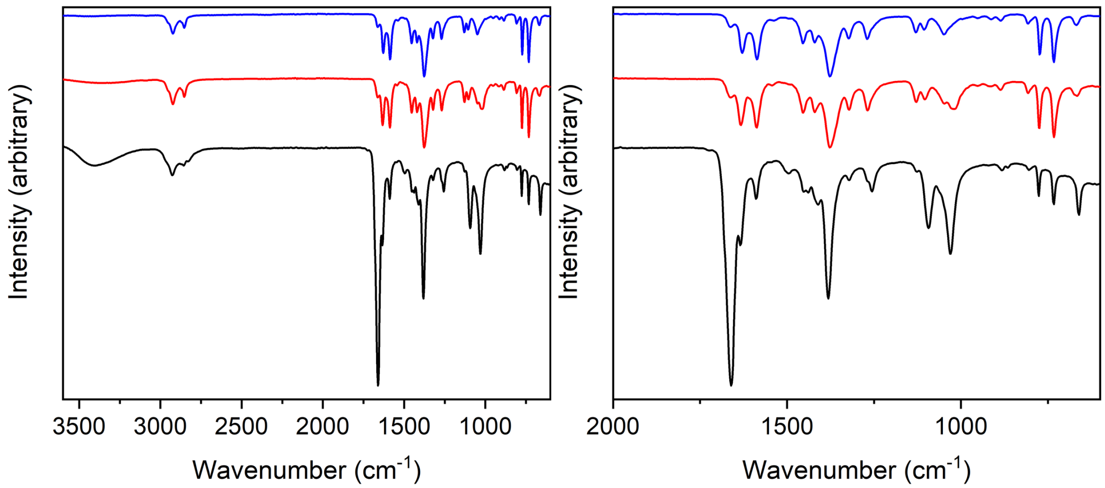

Figure S59. Infrared spectrum of Cu_{24}(5-undecoxy-bdc)$_{24}$ cage. Black: As synthesized material, Red: Methanol washed material, Blue: activated material.

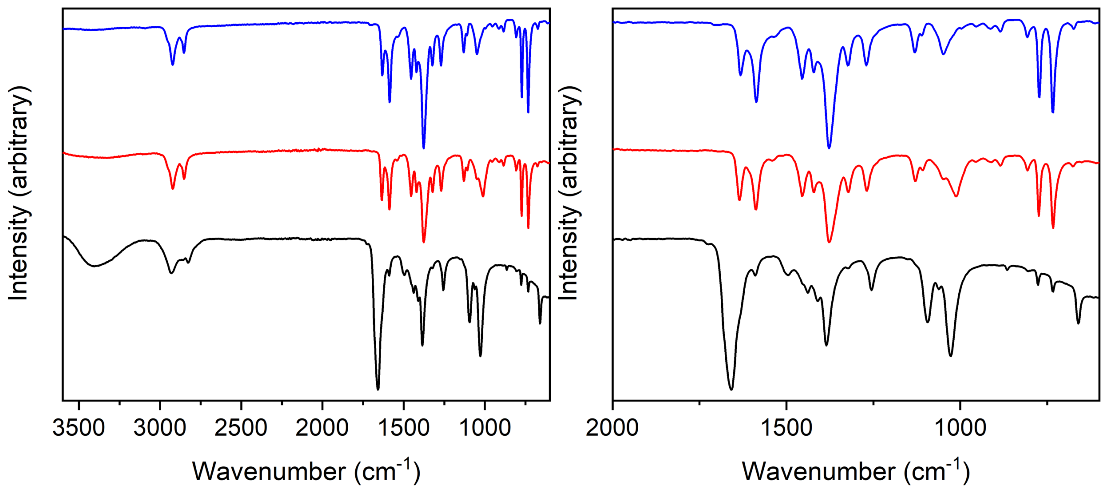

Figure S60. Infrared spectrum of Cu_{24}(5-dodecoxy-bdc)$_{24}$ cage. Black: As synthesized material, Red: Methanol washed material, Blue: activated material.

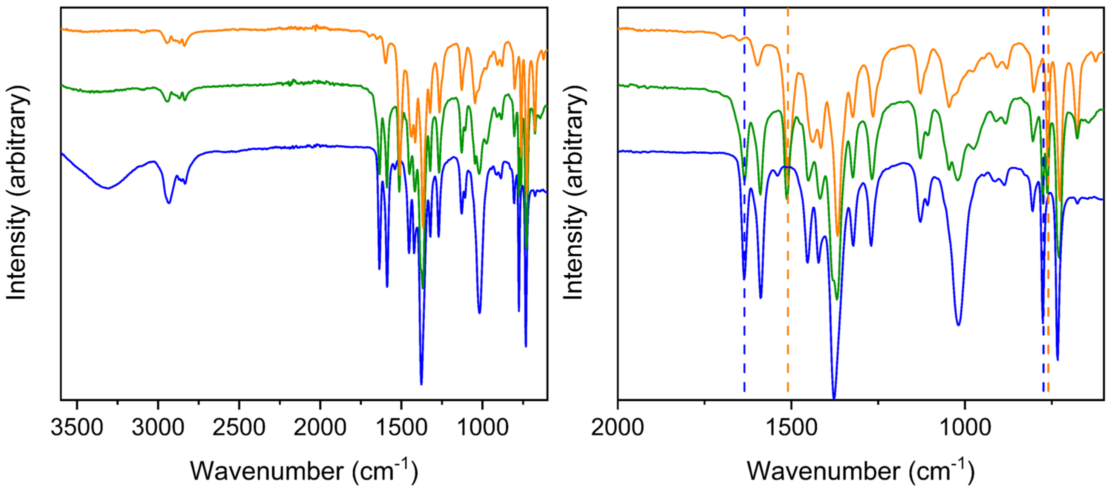

Figure S61. Infrared spectrum of Mo_{24}(5-hexoxy-bdc)$_{24}$ (orange), CuMo(5-hexoxy-bdc) alloy (green) and Cu_{24}(5-hexoxy-bdc)$_{24}$ (blue) after washing with MeOH.

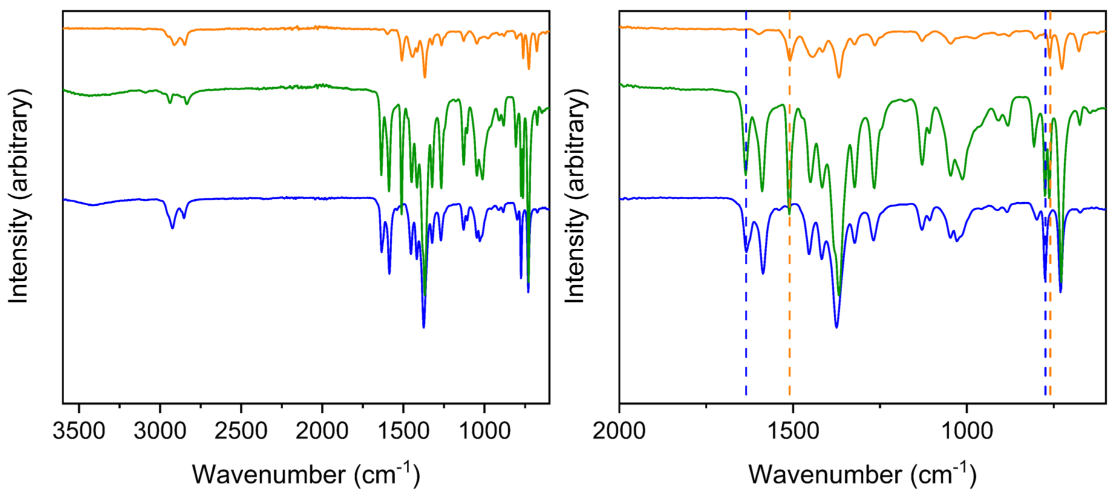

Figure S62. Infrared spectrum of Mo_{24}(5-nonoxy-bdc)$_{24}$ (orange), CuMo(5-nonoxy-bdc) alloy (green) and Cu_{24}(5-nonoxy-bdc)$_{24}$ (blue) after washing with MeOH.

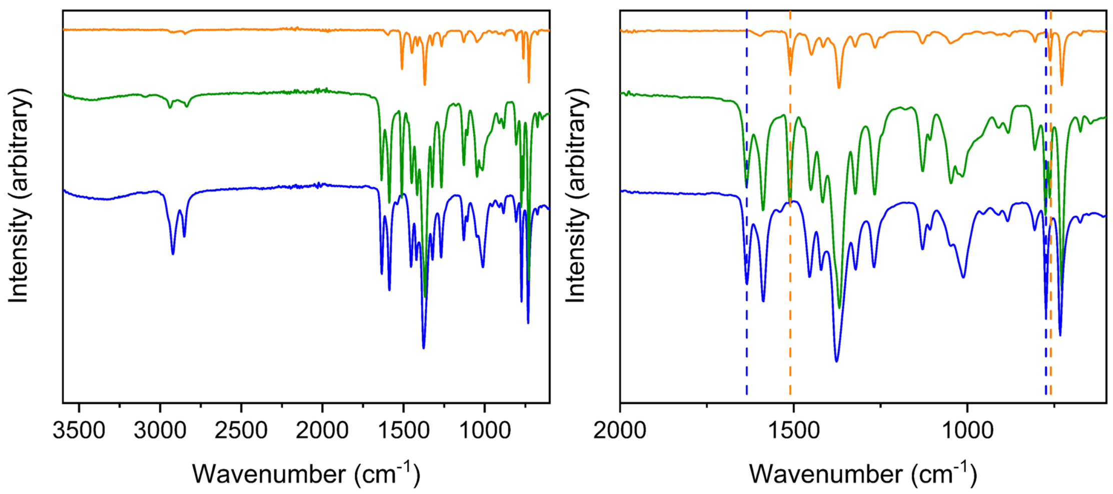

Figure S63. Infrared spectrum of Mo_{24}(5-dodecoxy-bdc)$_{24}$ (orange), CuMo(5-dodecoxy-bdc) alloy (green) and Cu_{24}(5-dodecoxy-bdc)$_{24}$ (blue) after washing with MeOH.

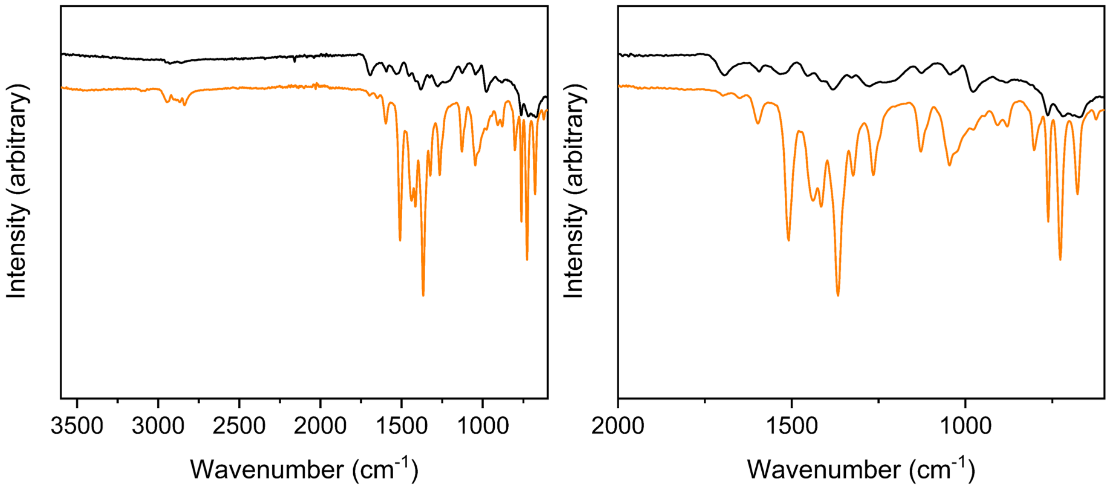

Figure S64. Infrared spectrum of MeOH washed Mo_{24}(5-hexoxy-bdc)$_{24}$ (orange) and Mo_{24}(5-hexoxy-bdc) (black) post activation at 250 °C and dosing with O_2 (black).

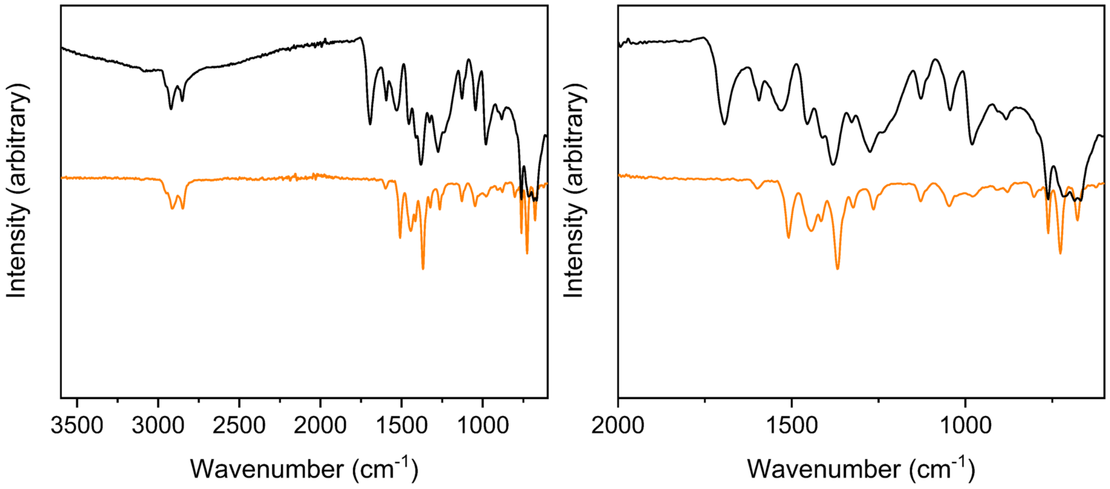

Figure S65. Infrared spectrum of MeOH washed Mo_{24}(5-nonoxy-bdc)$_{24}$ (orange) and Mo_{24}(5-nonoxy-bdc)$_{24}$ (black) post activation at 250 °C and dosing with O_2 (black).

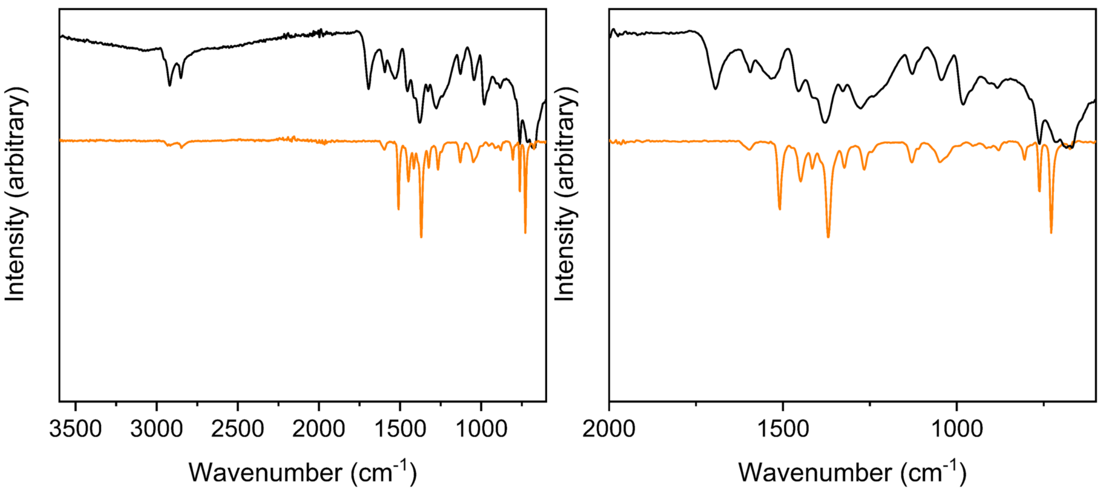

Figure S66. Infrared spectrum of MeOH washed Mo_{24}(5-dodecoxy-bdc)$_{24}$ (orange) and Mo_{24}(5-dodecoxy-bdc)$_{24}$ (black) post activation at 250 °C and dosing with O_2 (black).

Thermogravimetric Analysis:

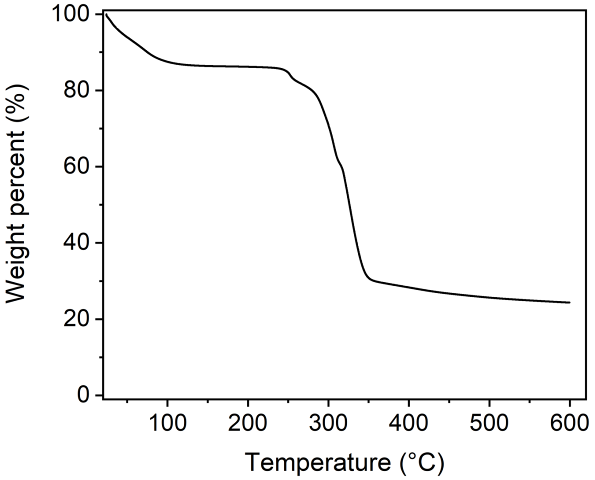

Figure S67. Thermogravimetric analysis of methanol washed Cu_{24}(5-hexoxy-bdc)$_{24}$ cage from 25 °C to 600 °C at a ramp rate of 2 degrees per minute.

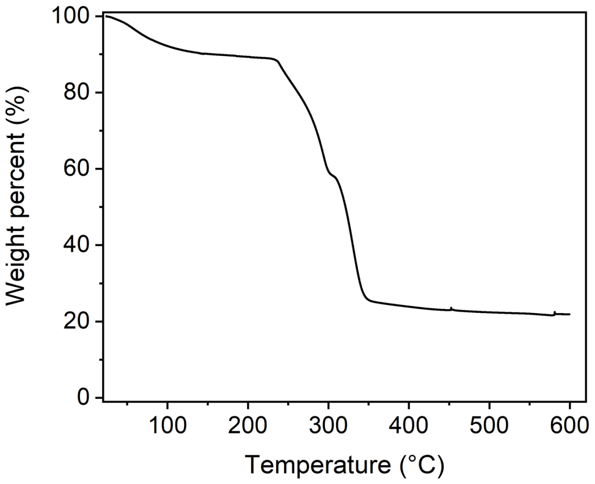

Figure S68. Thermogravimetric analysis of methanol washed Cu_{24}(5-heptoxy-bdc)$_{24}$ cage from 25 °C to 600 °C at a ramp rate of 2 degrees per minute.

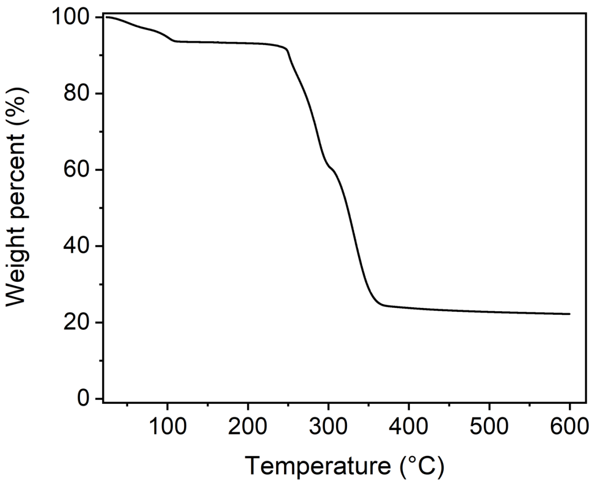

Figure S69. Thermogravimetric analysis of methanol washed Cu_{24}(5-octoxy-bdc)$_{24}$ cage from 25 °C to 600 °C at a ramp rate of 2 degrees per minute.

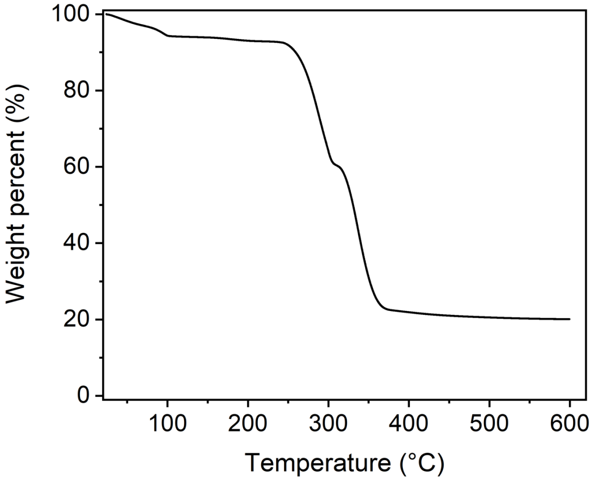

Figure S70. Thermogravimetric analysis of methanol washed Cu_{24}(5-nonoxy-bdc)$_{24}$ cage from 25 °C to 600 °C at a ramp rate of 2 degrees per minute.

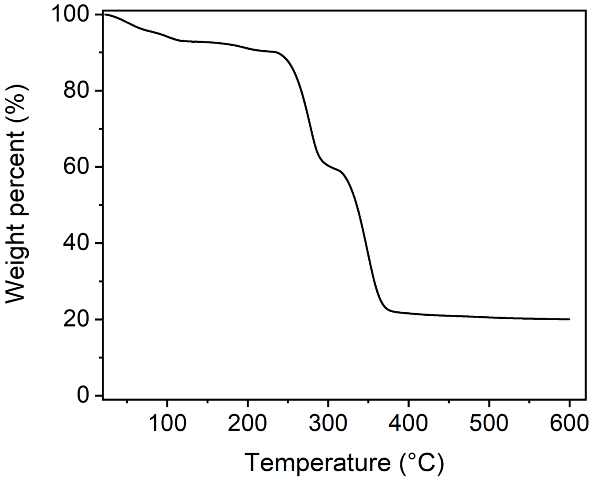

Figure S71. Thermogravimetric analysis of methanol washed Cu_{24}(5-decoxy-bdc)$_{24}$ cage from 25 °C to 600 °C at a ramp rate of 2 degrees per minute.

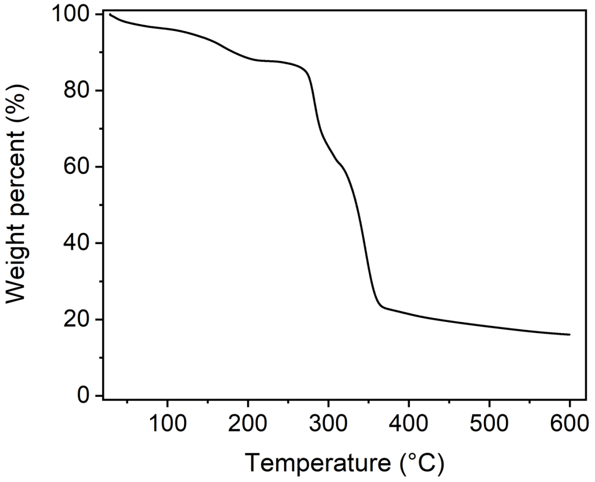

Figure S72. Thermogravimetric analysis of methanol washed Cu_{24}(5-undecoxy-bdc)$_{24}$ cage from 25 °C to 600 °C at a ramp rate of 2 degrees per minute.

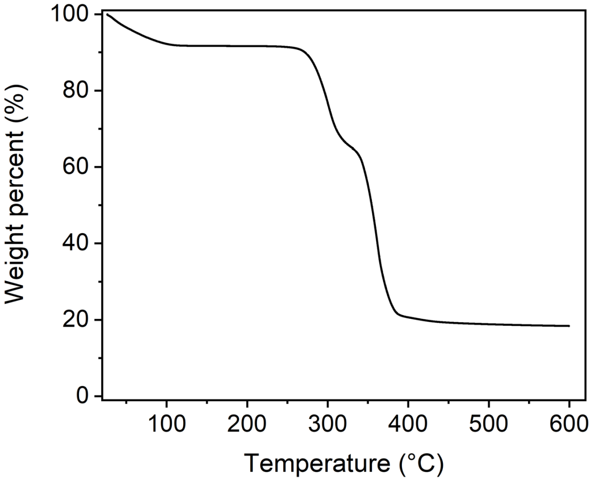

Figure S73. Thermogravimetric analysis of methanol washed Cu_{24}(5-dodecoxy-bdc)$_{24}$ from 25 °C to 600 °C at a ramp rate of 2 degrees per minute.

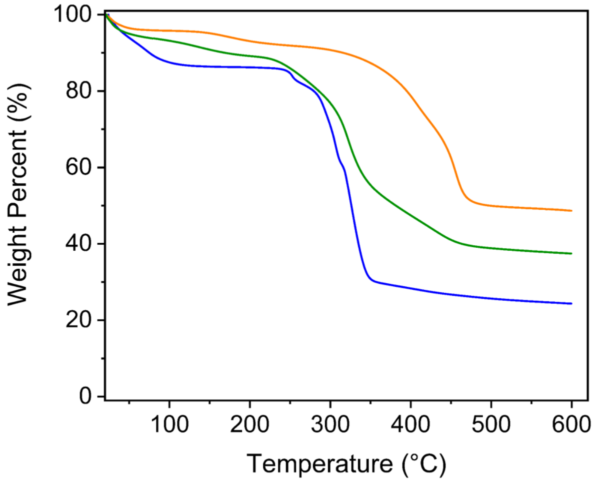

Figure S74. Thermogravimetric analysis of methanol washed Mo_{24}(5-hexoxy-bdc)$_{24}$ (orange), CuMo(5- hexoxy-bdc) alloy (green) and Cu_{24}(5- hexoxy-bdc)$_{24}$ (blue) from 25 °C to 600 °C at a ramp rate of 2 degrees per minute.

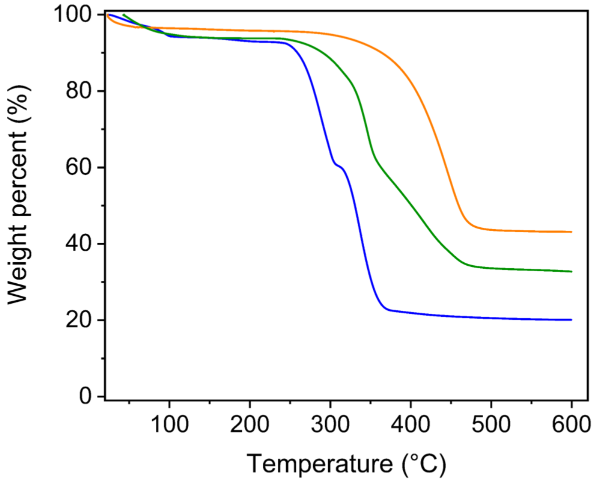

Figure S75. Thermogravimetric analysis of methanol washed Mo_{24}(5-nonoxy-bdc)$_{24}$ (orange), CuMo(5-nonoxy-bdc) alloy (green) and Cu_{24}(5-nonoxy-bdc)$_{24}$ (blue) from 25 °C to 600 °C at a ramp rate of 2 degrees per minute.

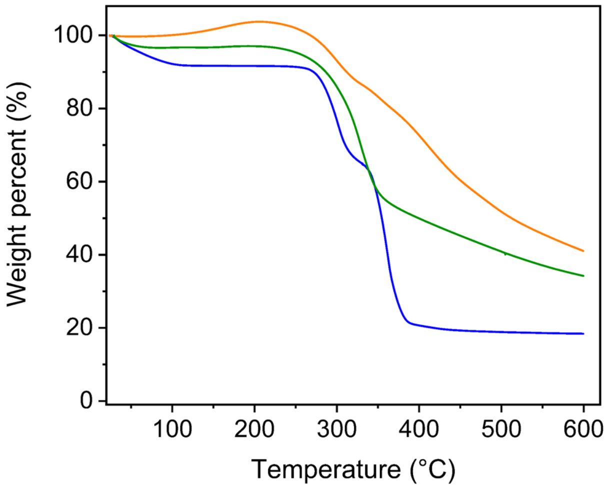

Figure S76. Thermogravimetric analysis of methanol washed Mo_{24}(5-dodecoxy-bdc)$_{24}$ (orange), CuMo(5-dodecoxy-bdc) alloy (green) and Cu_{24}(5-dodecoxy-bdc)$_{24}$ (blue) from 25 °C to 600 °C at a ramp rate of 2 degrees per minute.

Differential Scanning Calorimetry:

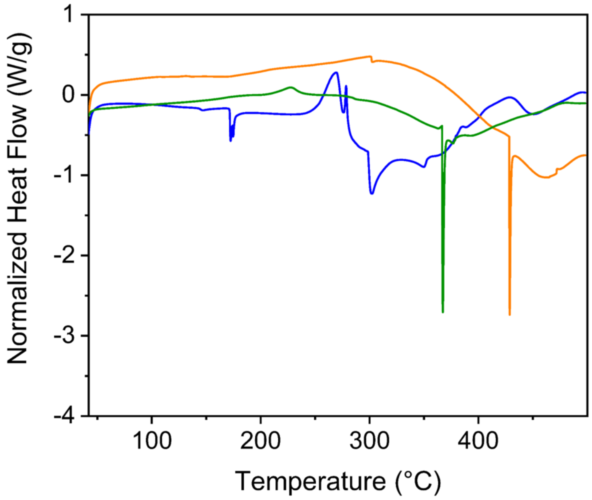

2

Figure S77. DSC analysis of activated Mo_{24}(5-hexoxy-bdc)$_{24}$ (orange), CuMo(5- hexoxy-bdc) alloy (green) and Cu_{24}(5- hexoxy-bdc)$_{24}$ (blue) from 40 °C to 500 °C at a ramp rate of 10 degrees per minute.

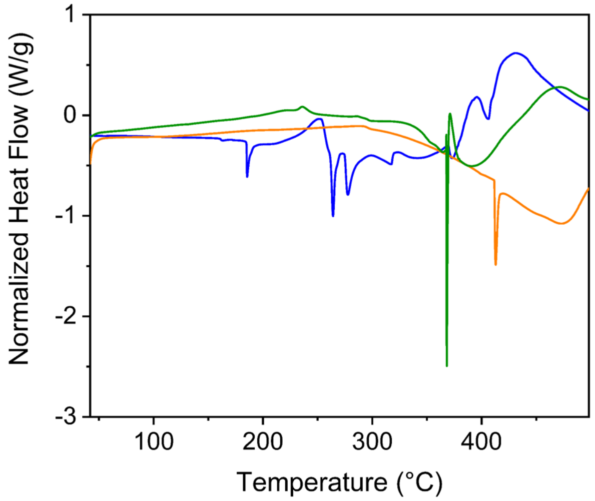

Figure S78. DSC analysis of activated Mo_{24}(5-nonoxy-bdc)$_{24}$ (orange), CuMo(5-nonoxy-bdc) alloy (green) and Cu_{24}(5-nonoxy-bdc)$_{24}$ (blue) from 40 °C to 500 °C at a ramp rate of 10 degrees per minute.

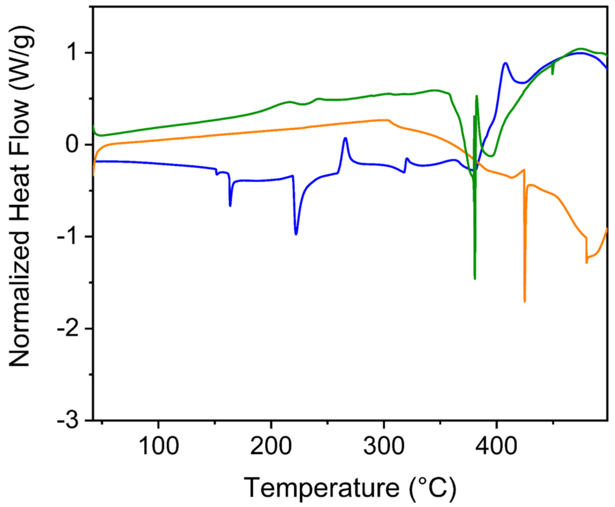

Figure S79. DSC analysis of activated Mo_{24}(5-dodecoxy-bdc)$_{24}$ (orange), CuMo(5-dodecoxy-bdc) alloy (green) and Cu_{24}(5-dodecoxy-bdc)$_{24}$ (blue) from 40 °C to 500 °C at a ramp rate of 10 degrees per minute.

X-Ray Photoelectron Spectroscopy Data:

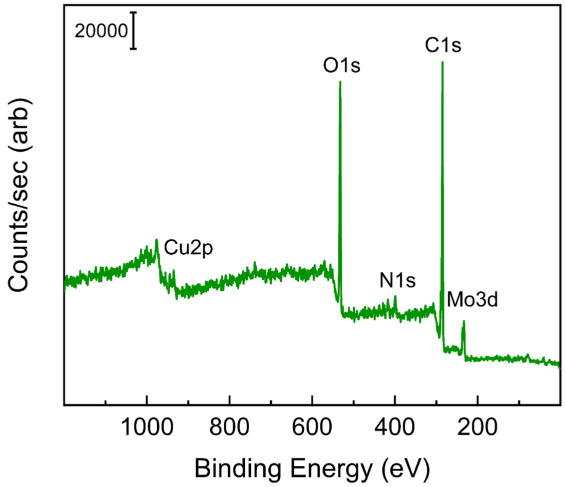

Figure S80. XPS survey spectra of methanol washed CuMo(5-hexoxy-bdc) alloy.

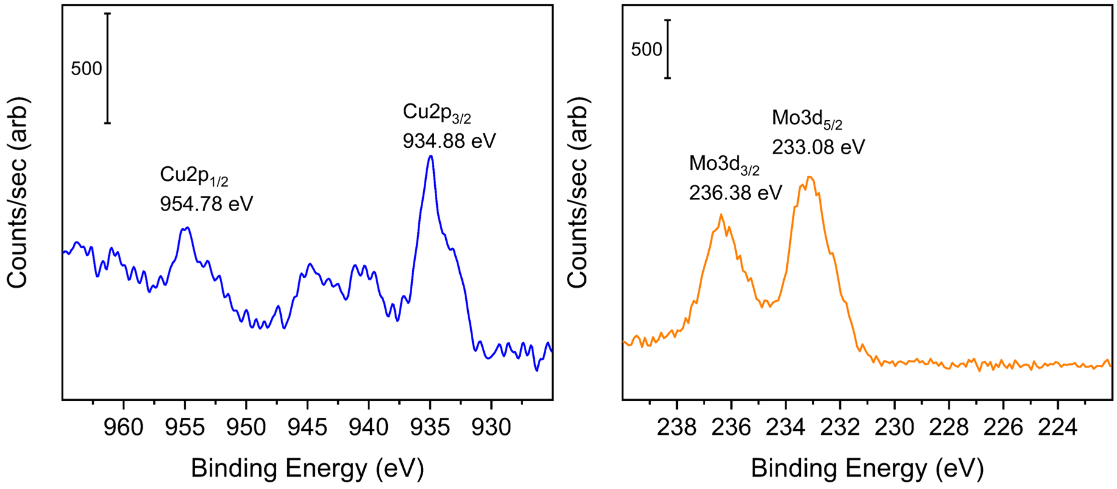

Figure S81. XPS spectra recorded for high-resolution copper region (left) and high-resolution molybdenum region (right) of CuMo(5-hexoxy-bdc) alloy. The atomic % of copper and molybdenum in the methanol washed alloy is 43.19% and 56.81% respectively.

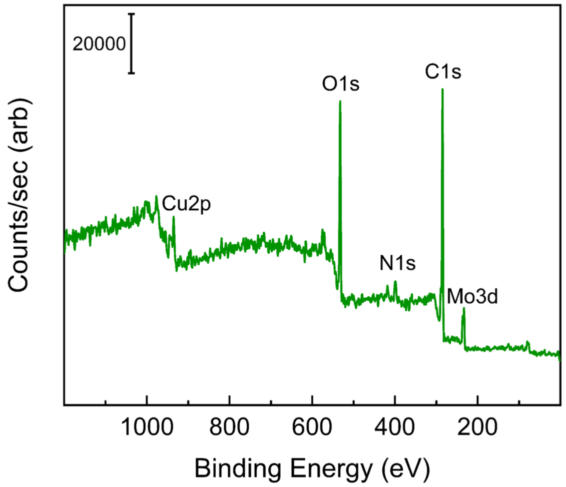

Figure S82. XPS survey spectra of methanol washed CuMo(5-nonoxy-bdc) alloy.

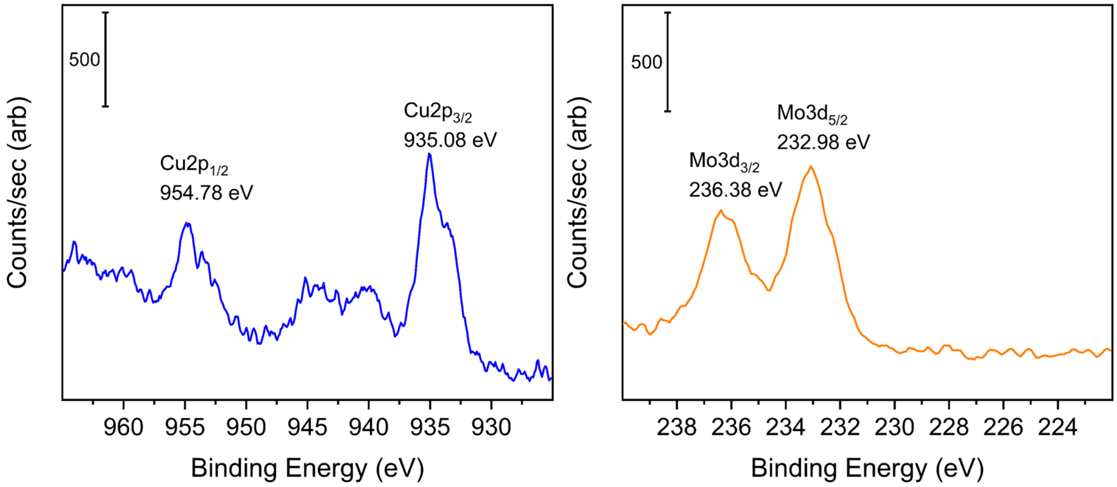

Figure S83. XPS spectra recorded for high-resolution copper region (left) and high-resolution molybdenum region (right) of CuMo(5-nonoxy-bdc) alloy. The atomic % of copper and molybdenum in the methanol washed alloy is 62.18% and 37.82% respectively.

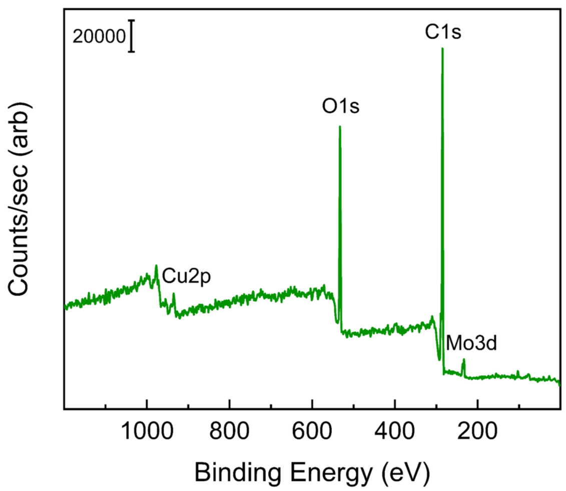

Figure S84. XPS survey spectra of methanol washed CuMo(5-dodecoxy-bdc) alloy.

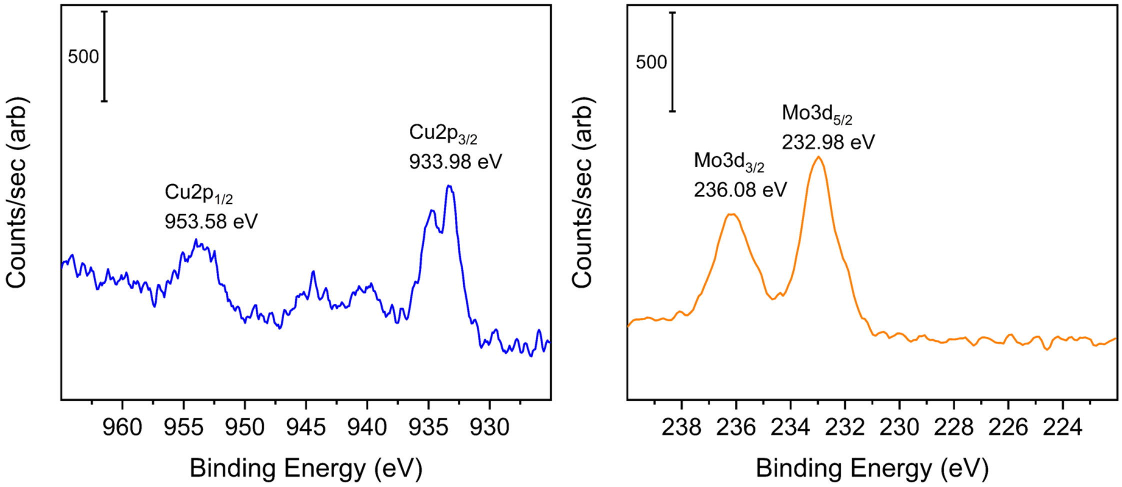

Figure S85. XPS spectra recorded for high-resolution copper region (left) and high-resolution molybdenum region (right) of CuMo(5-dodecoxy-bdc) alloy. The atomic % of copper and molybdenum in the methanol washed alloy is 58.87% and 41.13% respectively.

Scanning Electron Microscopy Images/Energy Dispersive X-ray Analysis:

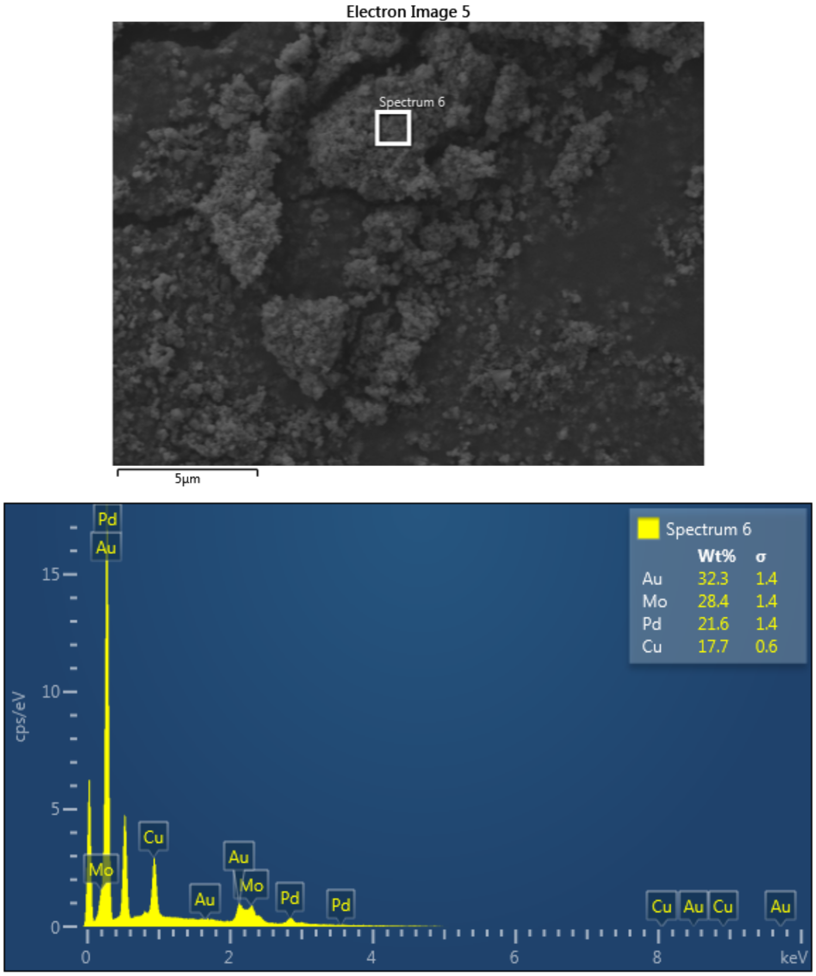

Figure S86. SEM image of MeOH washed CuMo(5-hexoxy-bdc) alloy at 5,000x magnification (top) and energy dispersive x-ray analysis of elements after Pd/Au coating of sample.

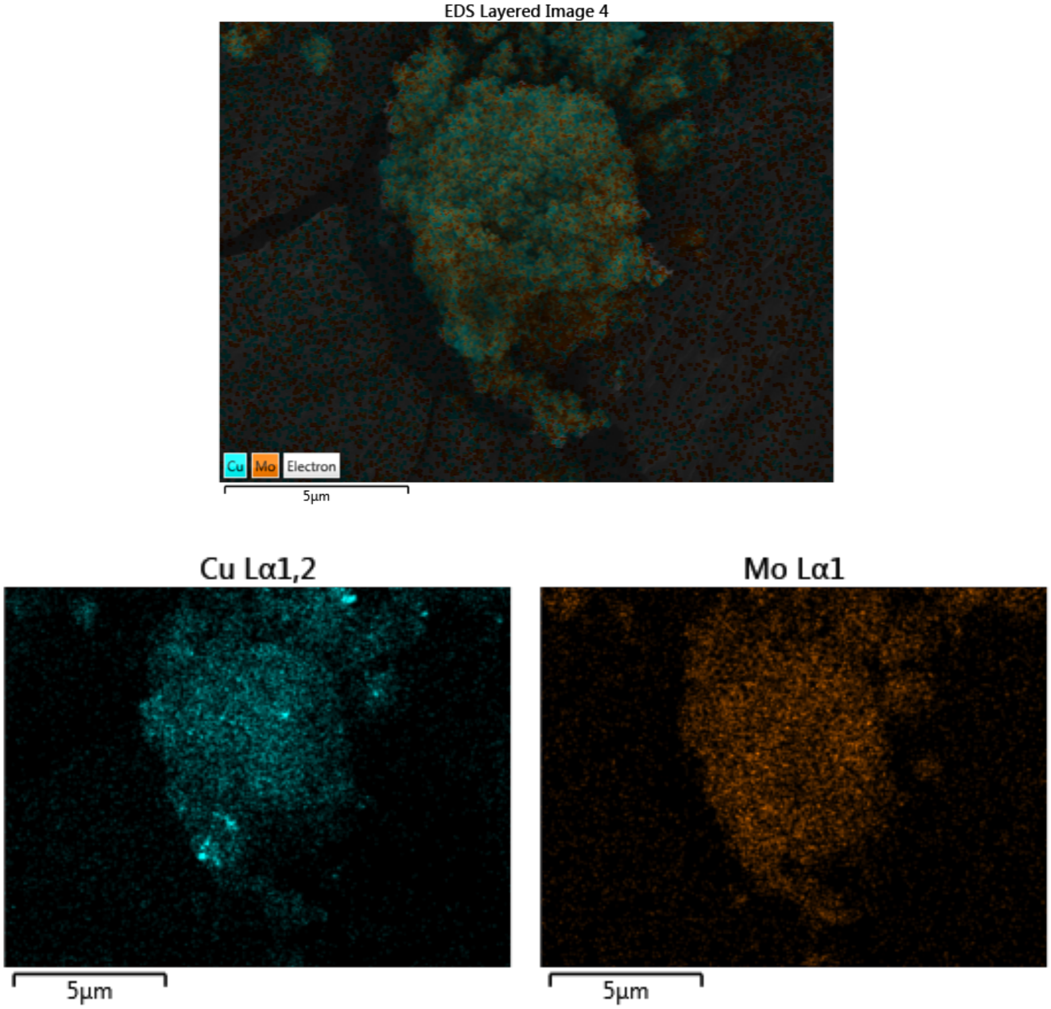

Figure S87. Elemental mapping of MeOH washed CuMo(5-hexoxy-bdc) alloy using 8 kV accelerating voltage after Pd/Au coating of sample.

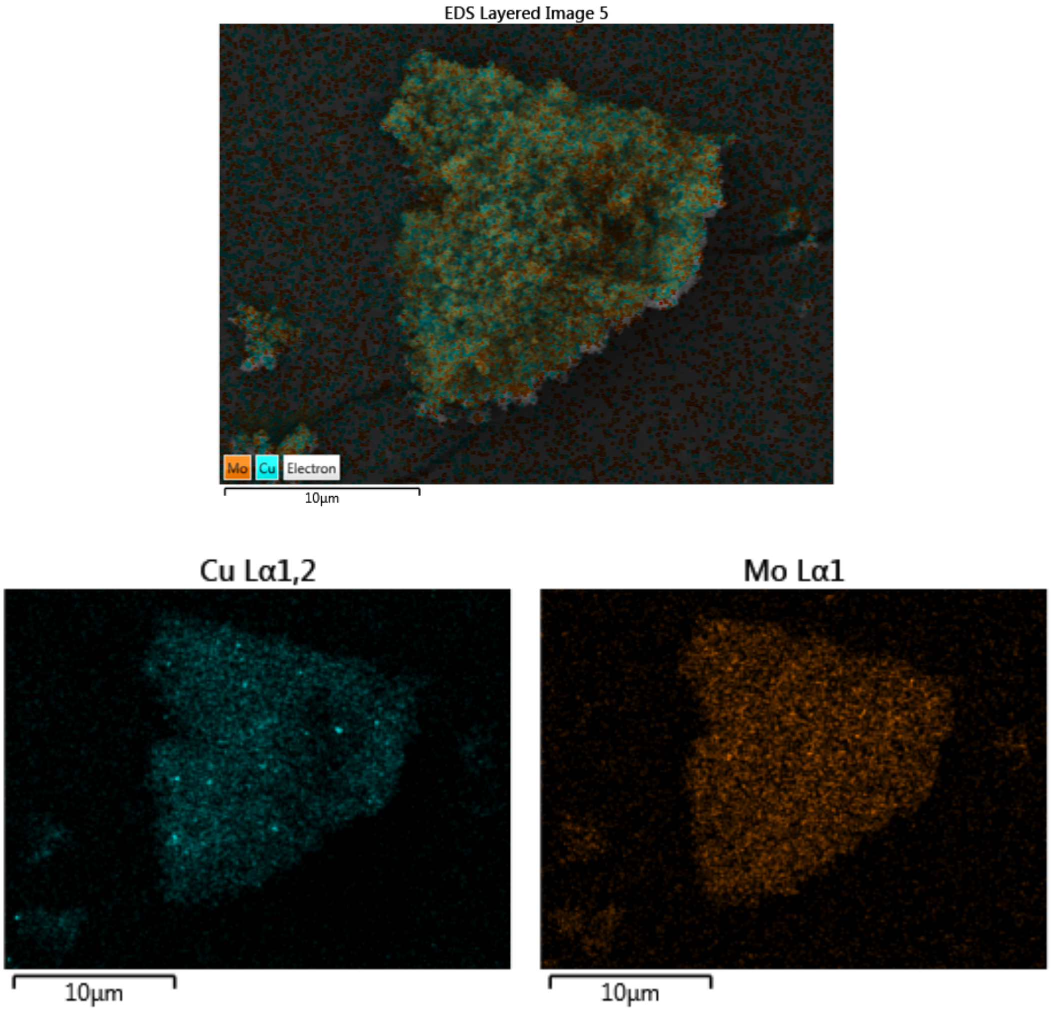

Figure S88. Elemental mapping of MeOH washed CuMo(5-hexoxy-bdc) alloy using 8 kV accelerating voltage after Pd/Au coating of sample.

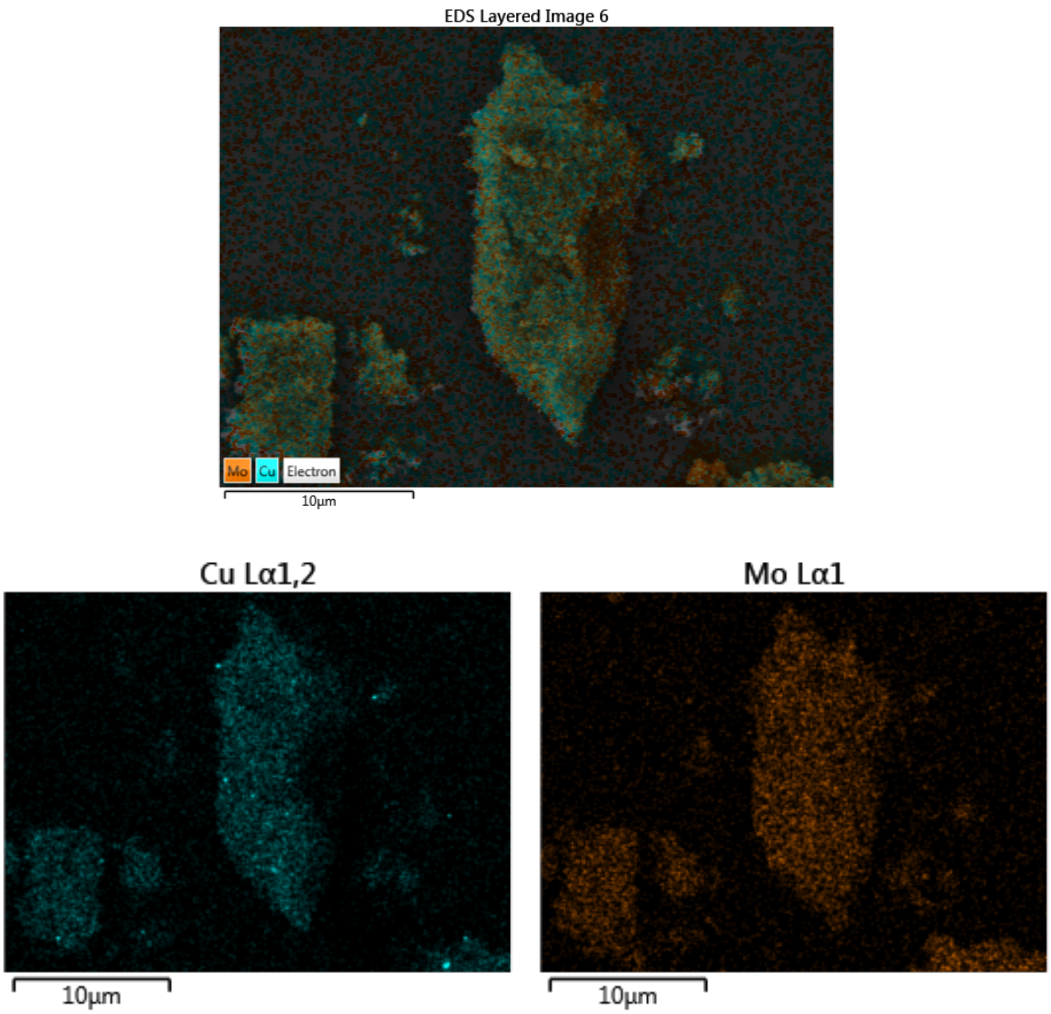

Figure S89. Elemental mapping of MeOH washed CuMo(5-hexoxy-bdc) alloy using 8 kV accelerating voltage after Pd/Au coating of sample.

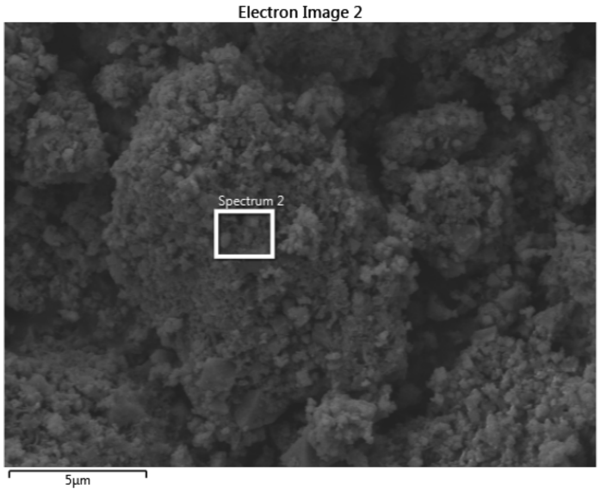

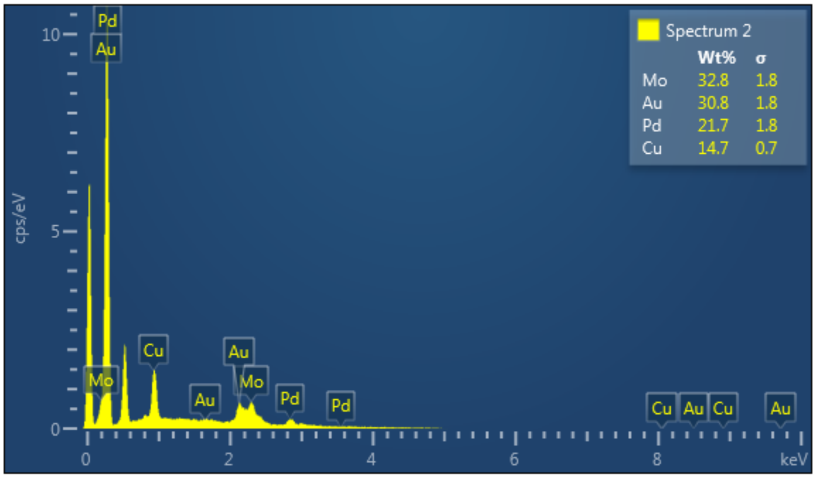

Figure S90. SEM image of MeOH washed CuMo(5-nonoxy-bdc) alloy at 5,000x magnification (top) and energy dispersive x-ray analysis of elements after Pd/Au coating of sample.

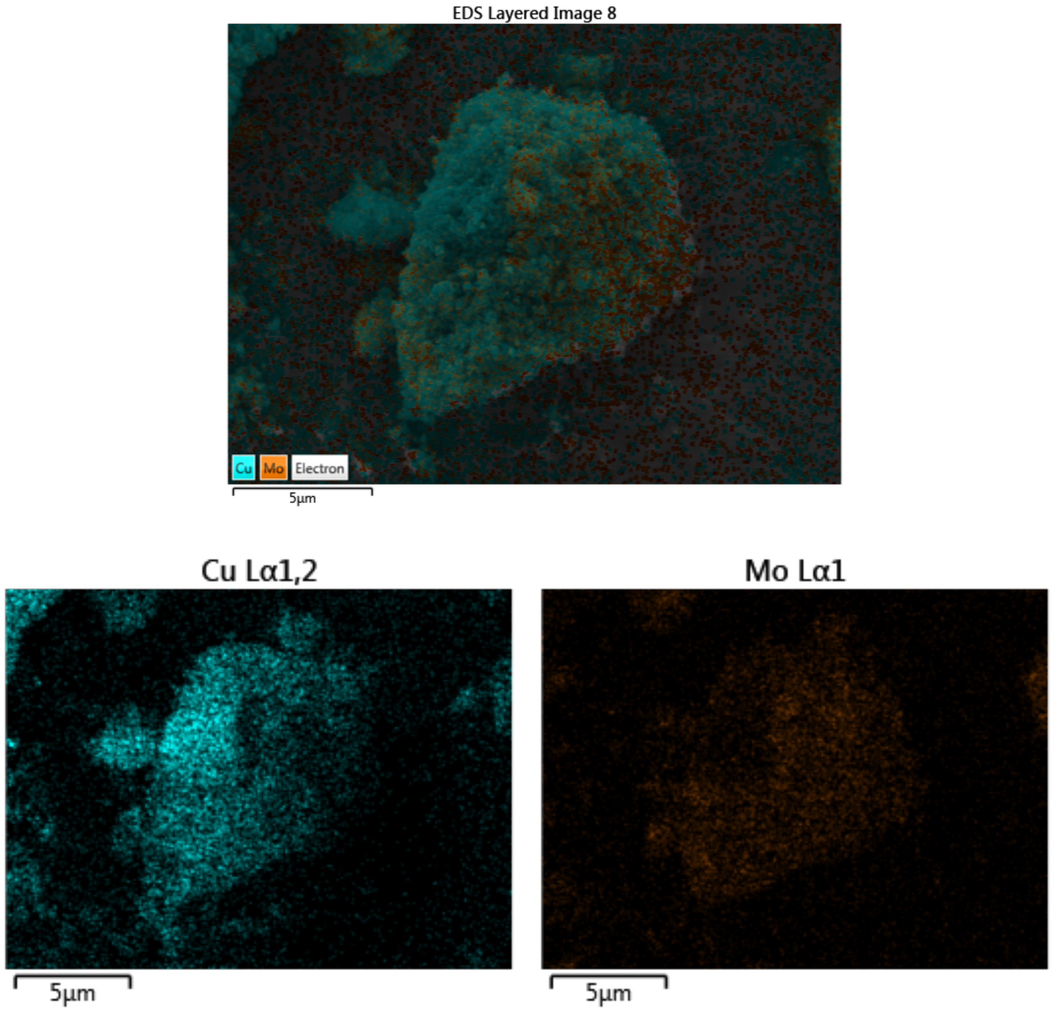

Figure S91. Elemental mapping of MeOH washed CuMo(5-nonoxy-bdc) alloy using 8 kV accelerating voltage after Pd/Au coating of the sample.

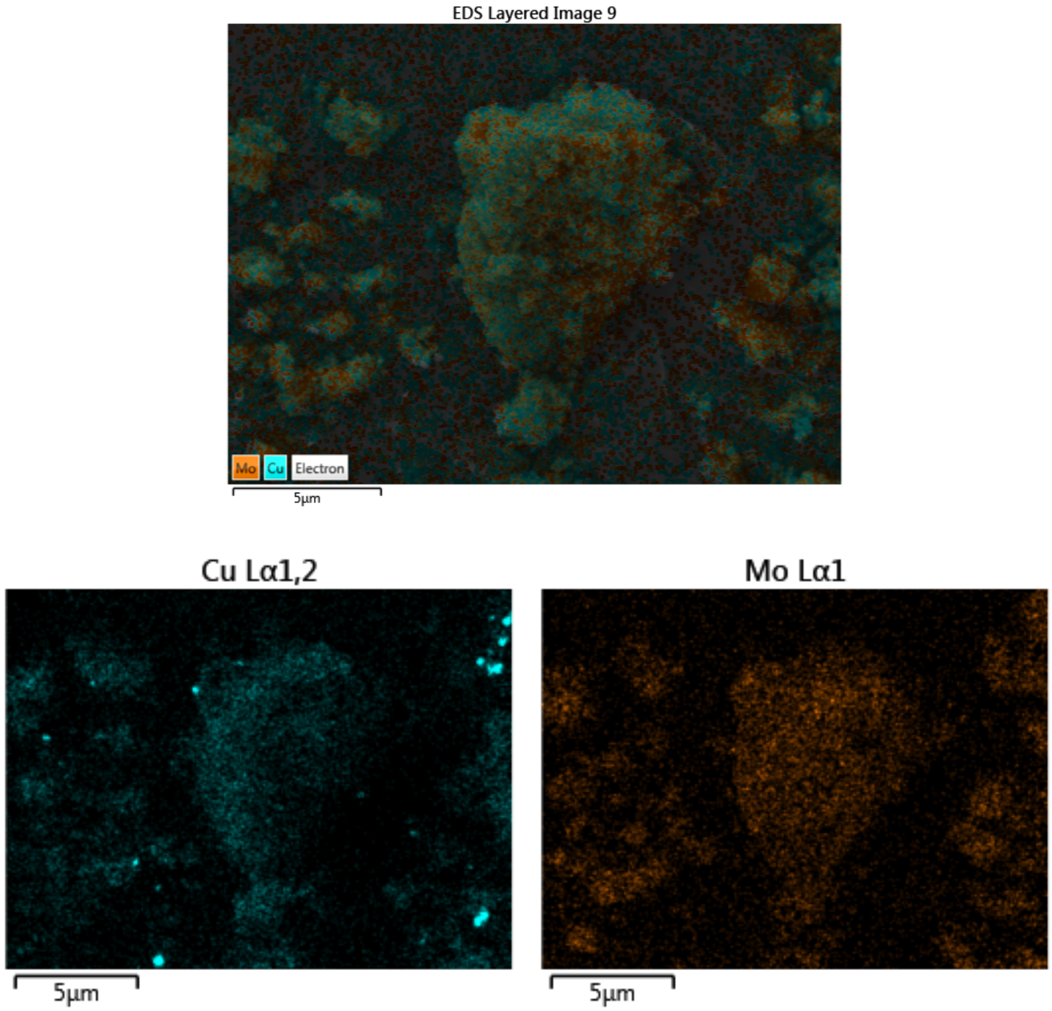

Figure S92. Elemental mapping of MeOH washed CuMo(5-nonoxy-bdc) alloy using 8 kV accelerating voltage after Pd/Au coating of the sample.

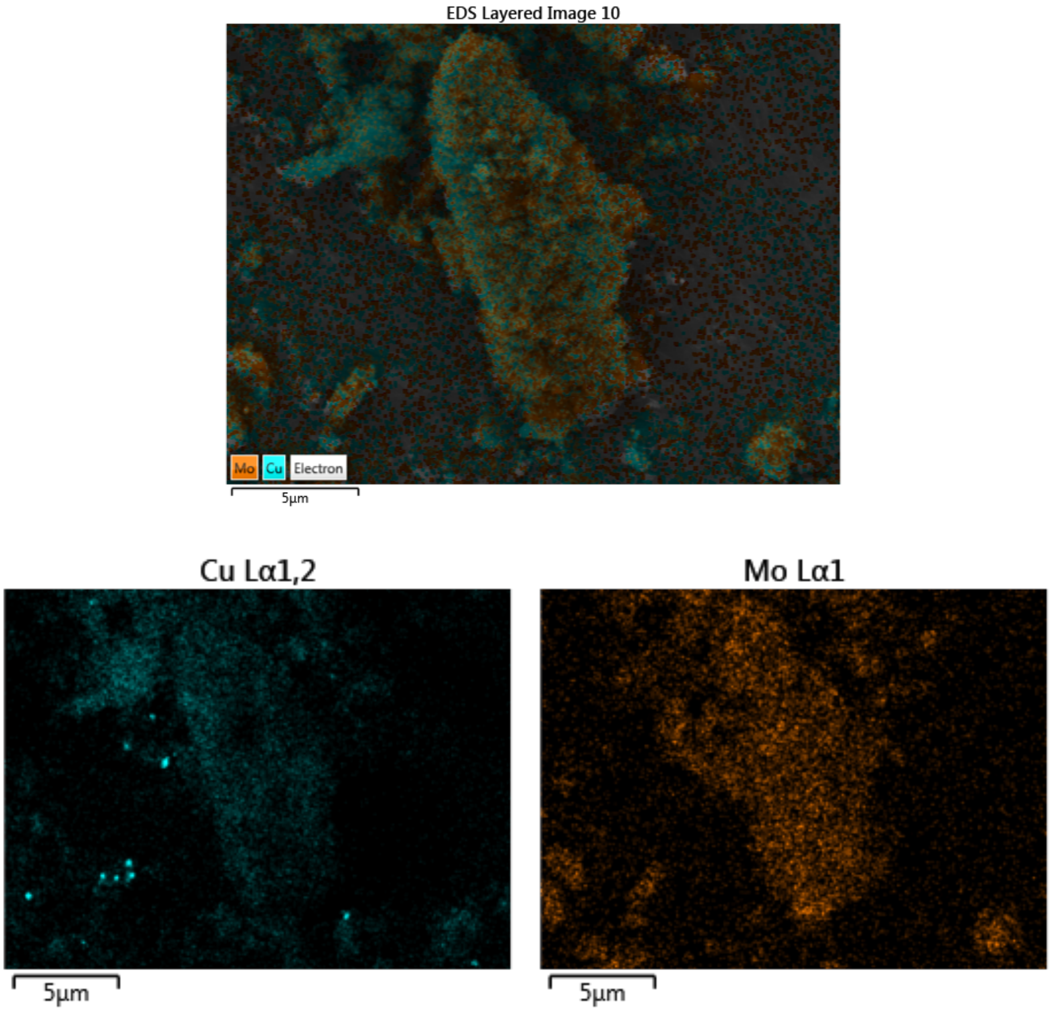

Figure S93. Elemental mapping of MeOH washed CuMo(5-nonoxy-bdc) alloy using 8 kV accelerating voltage after Pd/Au coating of the sample.

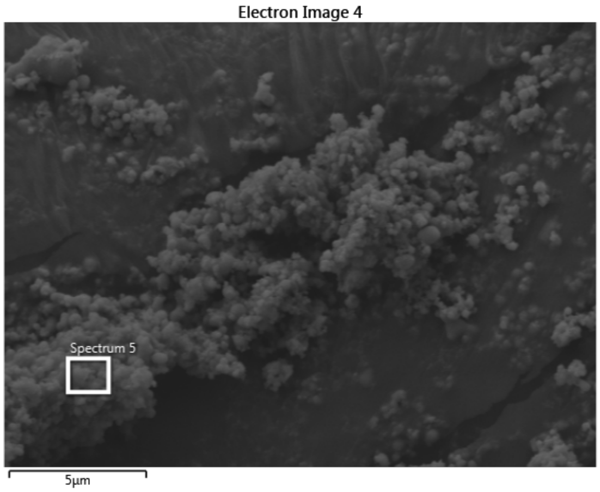

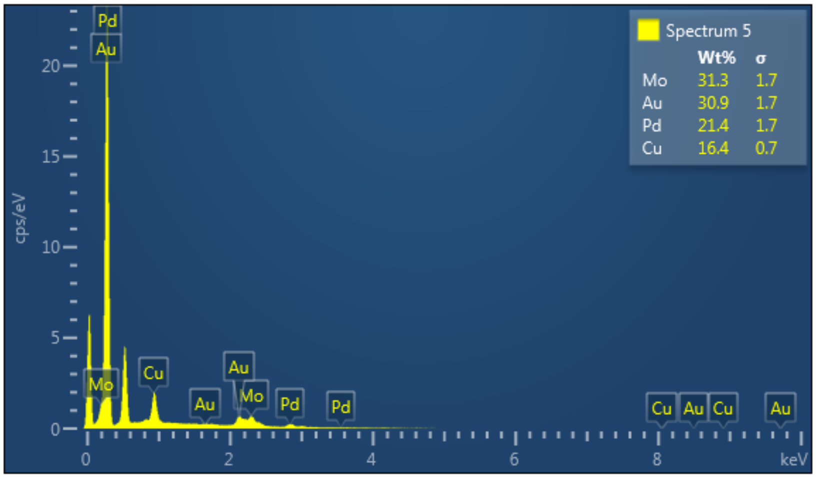

Figure S94. SEM image of MeOH washed CuMo(5-dodecoxy-bdc) alloy at 5,000x magnification (top) and energy dispersive x-ray analysis of elements after Pd/Au coating of sample.

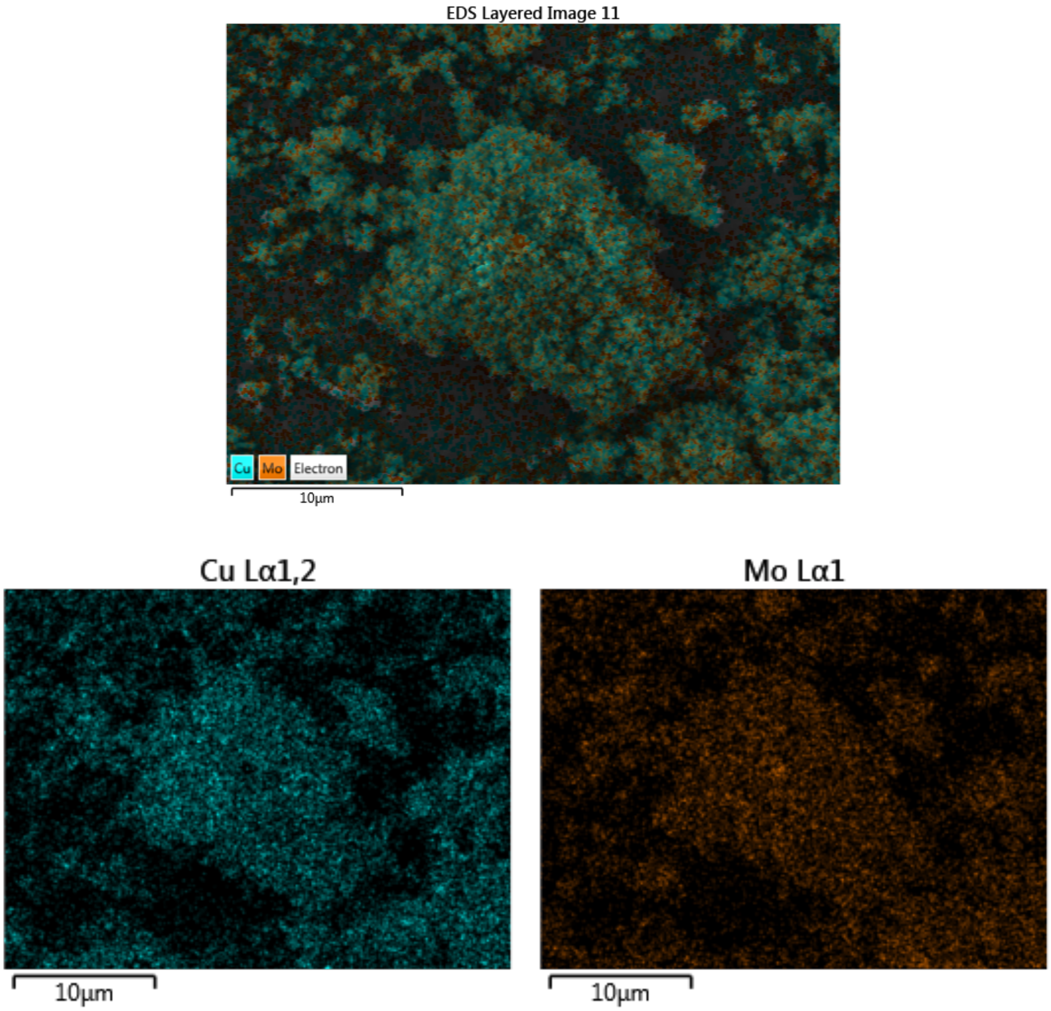

Figure S95. Elemental mapping of MeOH washed CuMo(5-dodecoxy-bdc) alloy using 8 kV accelerating voltage after Pd/Au coating of the sample.

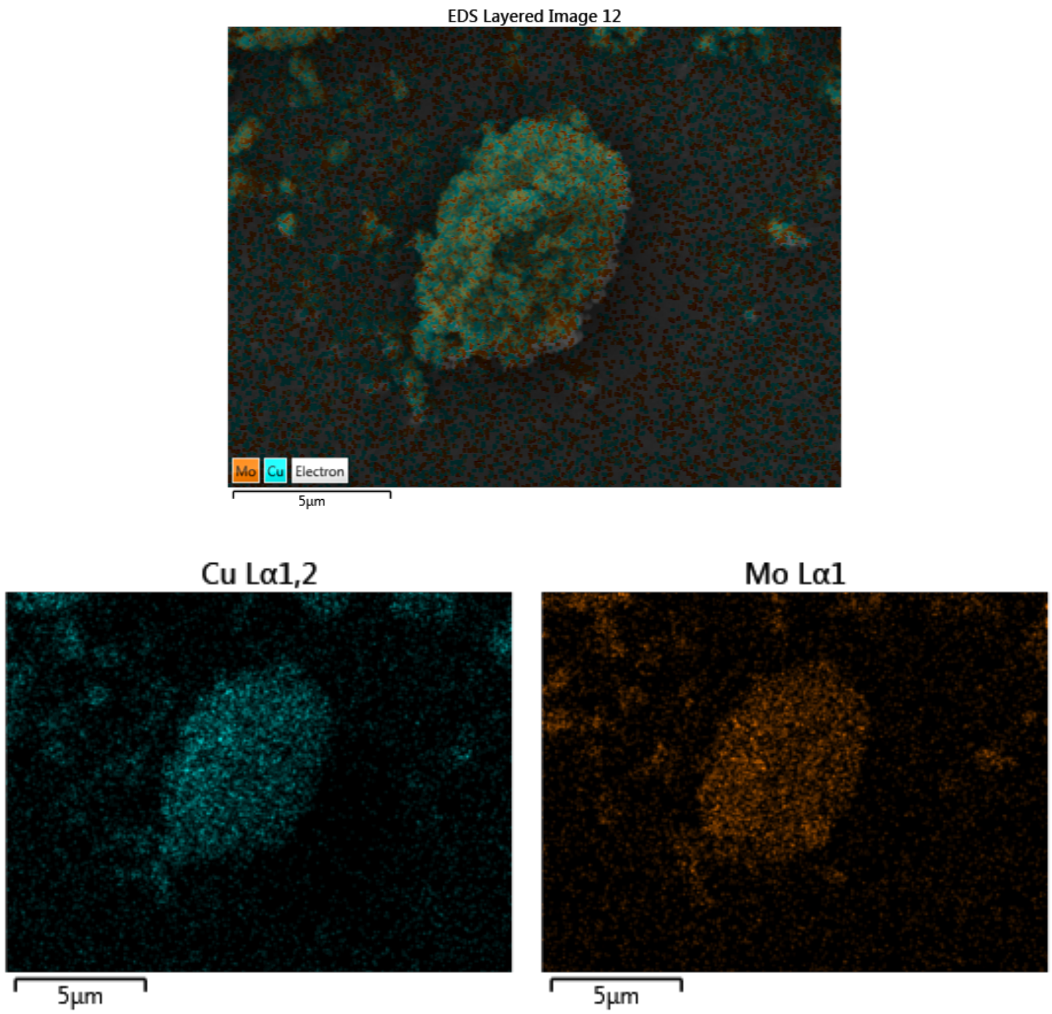

Figure S96. Elemental mapping of MeOH washed CuMo(5-dodecoxy-bdc) alloy using 8 kV accelerating voltage after Pd/Au coating of the sample.

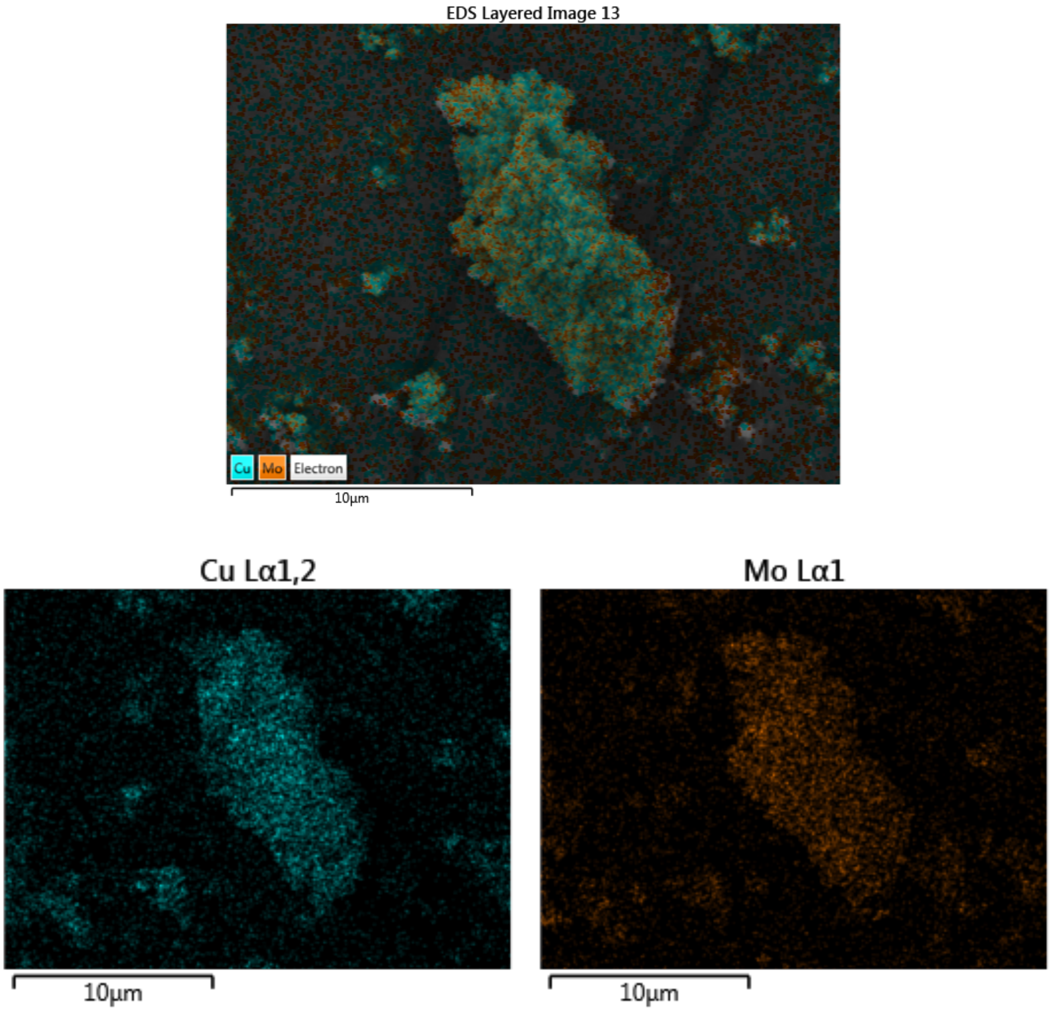

Figure S97. Elemental mapping of MeOH washed CuMo(5-dodecoxy-bdc) alloy using 8 kV accelerating voltage after Pd/Au coating of the sample.

Gas Adsorption Measurements:

Dinitrogen and Carbon dioxide were used for adsorption measurements. To measure the isotherms, a Micromeritics 3Flex gas adsorption analyzer was used. Samples were loaded into weighed analysis tubes under N_2 atmosphere to prevent oxidation or water absorption into samples. The tubes were capped with Transeals and removed from the glovebox. They were activated under vacuum at various temperatures on a Smartvac degas system until the static outgas rate was less than 2 μbar/min. After degassing, the tube was removed from the Smartvac under vacuum and weighed to determine the mass of the sample in the tube. For cryogenic N_2 measurements, an isothermal jacket was fitted on the tube. Degas surveys using CO_2 at 195 K or N_2 at 77 K were performed on the samples after they were heated in increments of 25 °C to determine the optimal activation temperature. Langmuir Surface Areas were calculated via the Micromeritics software. BET calculations were calculated via the first and second consistency check.[6] Both N_2 and CO_2 sorption measurements were performed on some materials due to the lack of porosity to N_2.

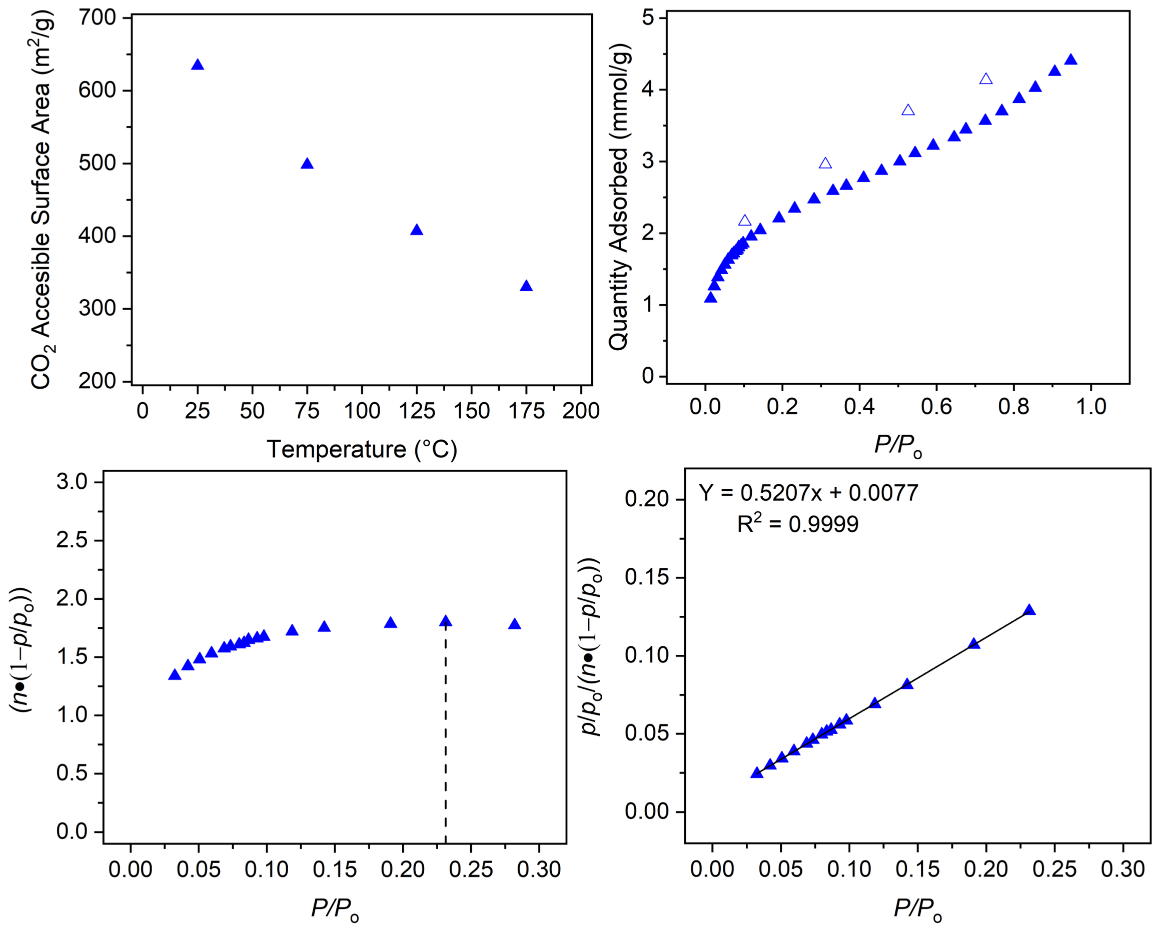

Figure S98. Degas survey (top left) and full BET isotherm (top right) of Cu_{24}(5-hexoxy-bdc)$_{24}$ cage (CO_2) activated at 75 °C under flowing N_2. Plot of n•(1-P/P_o) vs. P/P_o of Cu_{24}(5-hexoxy-bdc)$_{24}$ (bottom left). According to the first BET consistency criterion, the BET linear fit can be determined via the maximum P/P_o, which is indicated by the dashed line. The slope of the line (bottom right) where P/P_o< 0.24 is 0.5207 with a y-intercept of 0.0077. These values satisfy the second BET consistency criterion and yield a CO_2 BET surface area of 196.608 m^2/g.

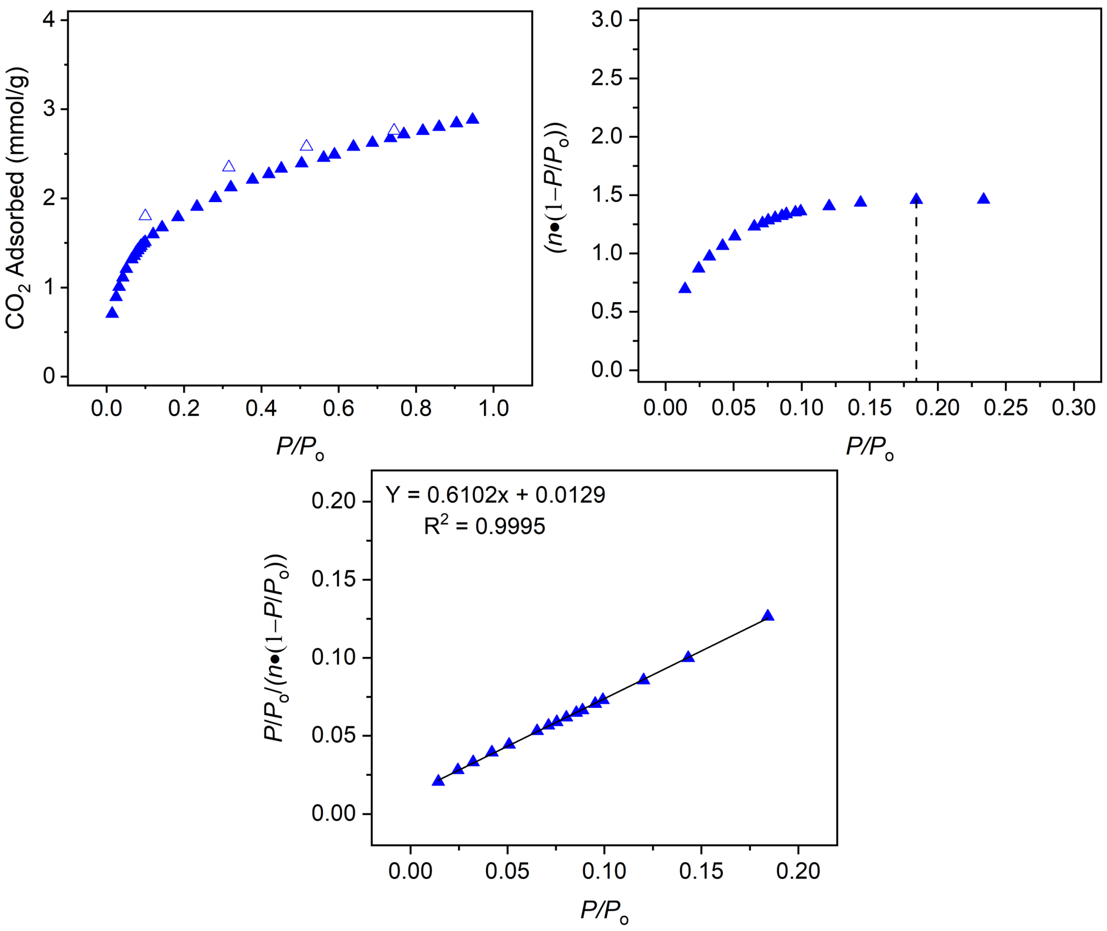

Figure S99. Full BET isotherm (top left) of Cu_{24}(5-heptoxy-bdc)$_{24}$ cage (CO_2) activated at 75 °C under flowing N_2. Plot of n•(1-P/P_o) vs. P/P_o of Cu_{24}(5-heptoxy-bdc)$_{24}$ (top right). According to the first BET consistency criterion, the BET linear fit can be determined via the maximum P/P_o, which is indicated by the dashed line. The slope of the line (bottom) where $P/P_o < 0.19$ is 0.6102 with a y-intercept of 0.0129. These values satisfy the second BET consistency criterion and yield a CO_2 BET surface area of 167.771 m^2/g.

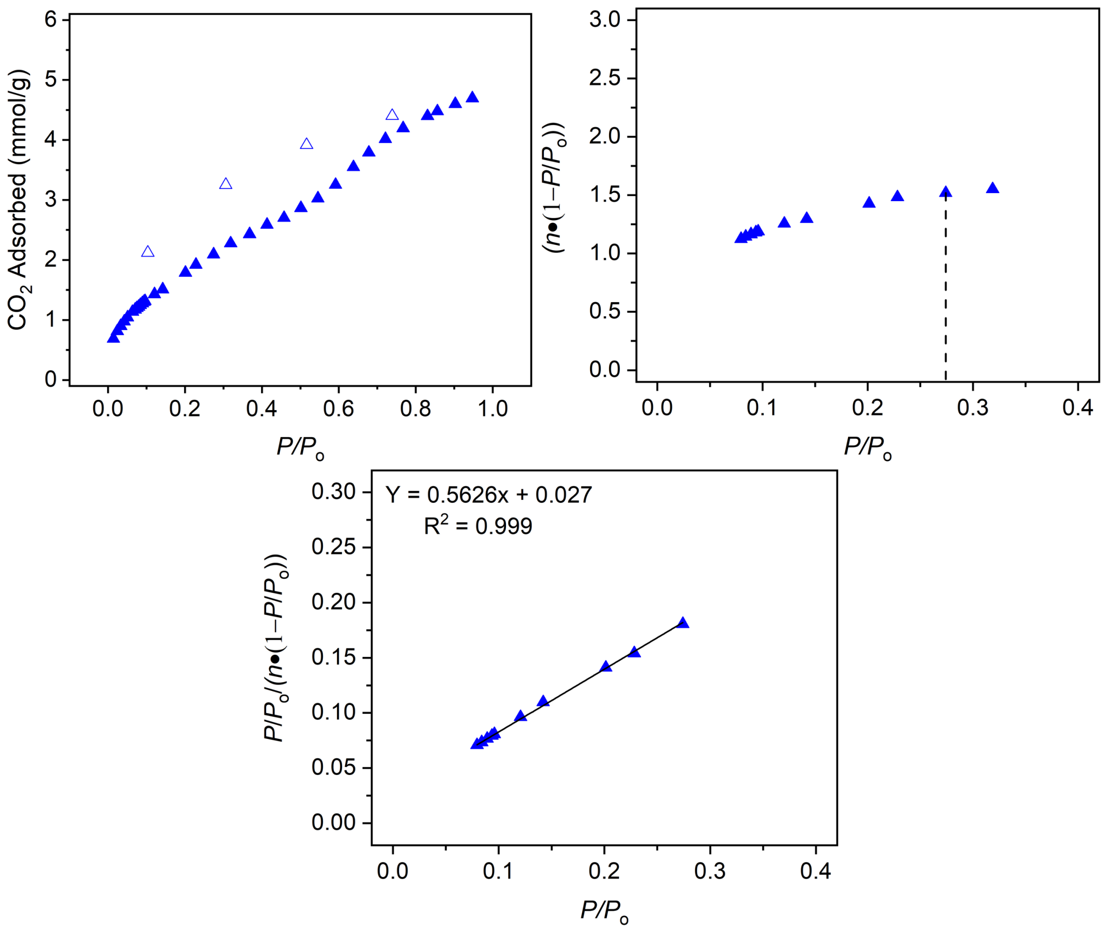

Figure S100. Full BET isotherm (top left) of Cu_{24}(5-octoxy-bdc)$_{24}$ cage (CO_2) activated at 75 °C under flowing N_2. Plot of n•(1-P/P_o) vs. P/P_o of Cu_{24}(5-octoxy-bdc)$_{24}$ (top right). According to the first BET consistency criterion, the BET linear fit can be determined via the maximum P/P_o, which is indicated by the dashed line. The slope of the line (bottom) where $P/P_o < 0.28$ is 0.5626 with a y-intercept of 0.027. These values satisfy the second BET consistency criterion and yield a CO_2 BET surface area of 181.966 m^2/g.

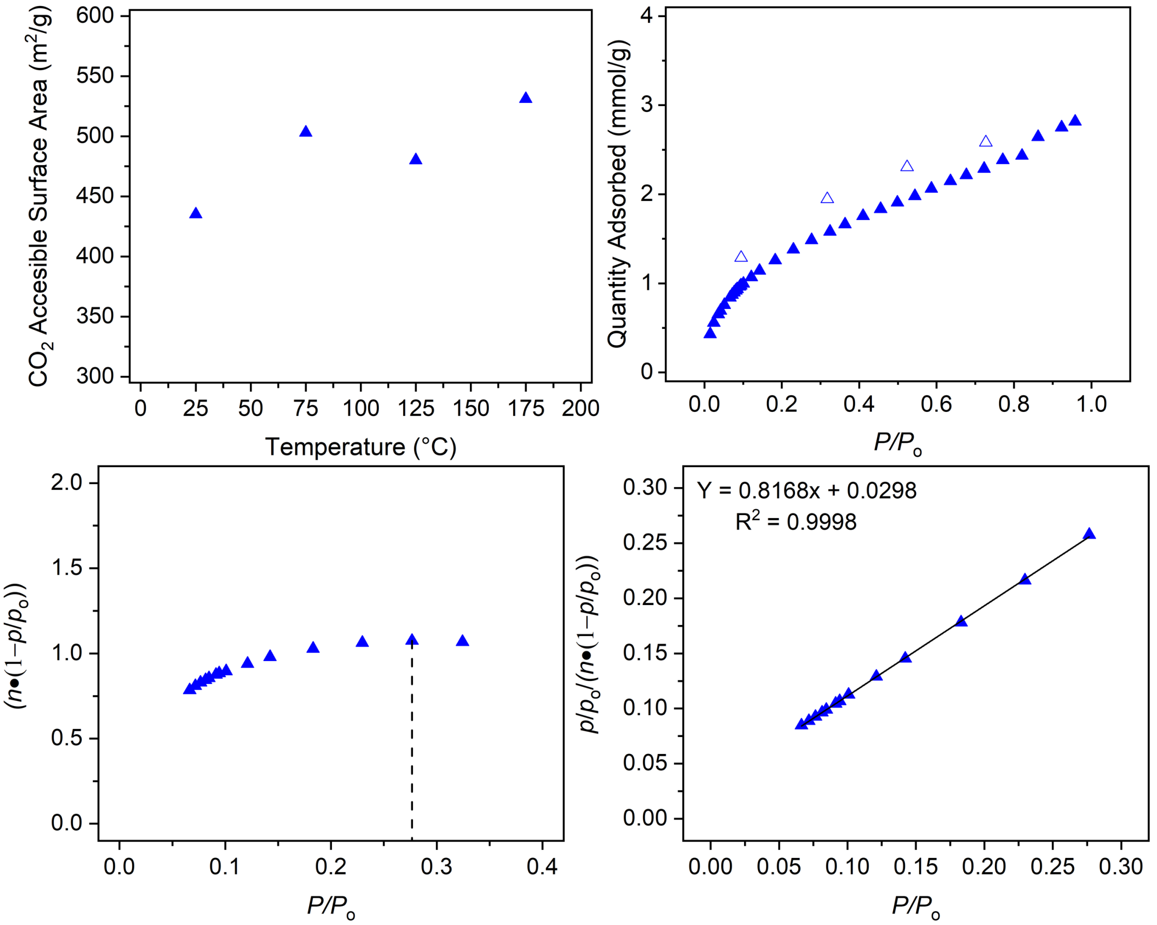

Figure S101. Degas survey (top left) and full BET isotherm (top right) of Cu_{24}(5-nonoxy-bdc)$_{24}$ cage (CO_2) activated at 75 °C under flowing N_2. Plot of n•(1-P/P_o) vs. P/P_o of Cu_{24}(5-nonoxy-bdc)$_{24}$ (bottom left). According to the first BET consistency criterion, the BET linear fit can be determined via the maximum P/P_o, which is indicated by the dashed line. The slope of the line (bottom right) where P/P_o< 0.28 is 0.8168 with a y-intercept of 0.0298. These values satisfy the second BET consistency criterion and yield a CO_2 BET surface area of 125.335 m^2/g.

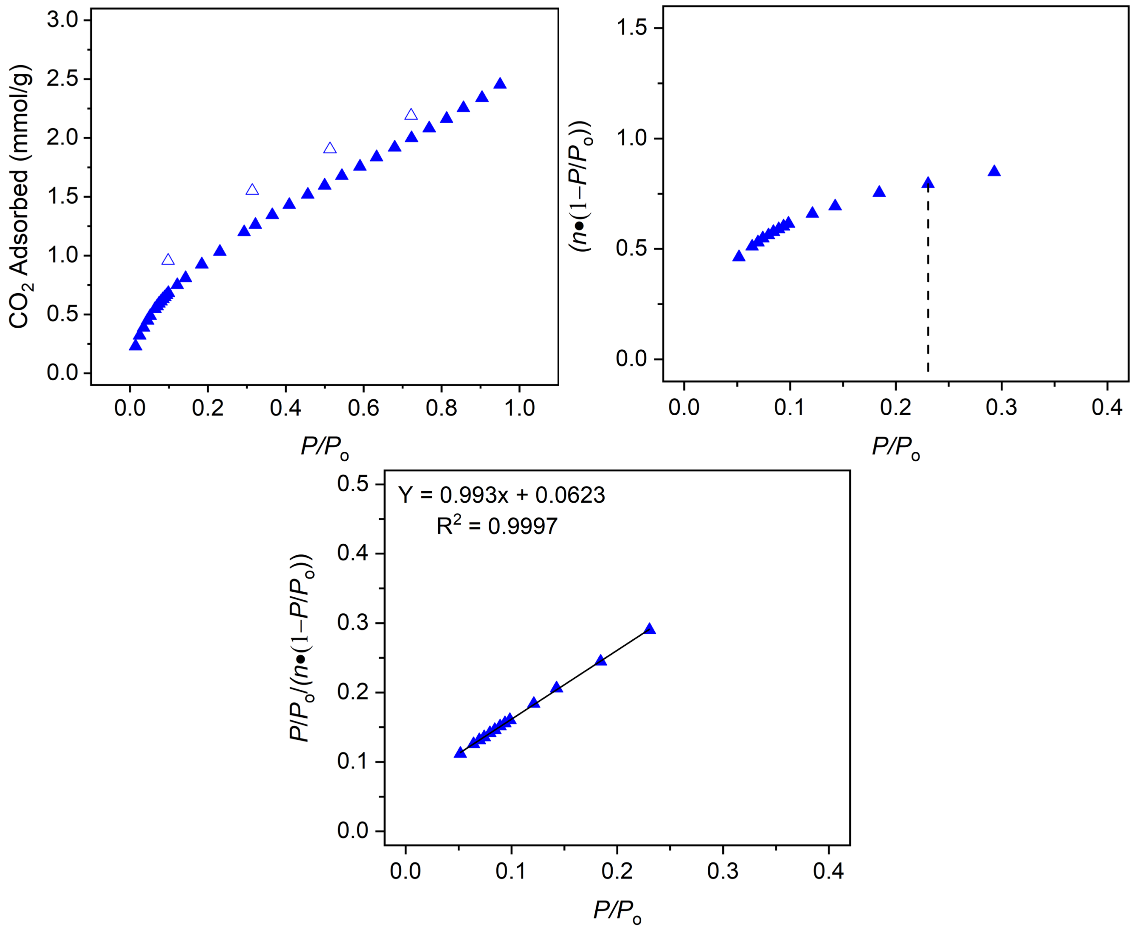

Figure S102. Full BET isotherm (top left) of Cu_{24}(5-decoxy-bdc)$_{24}$ cage (CO_2) activated at 75 °C under flowing N_2. Plot of n•(1-*P/P*$_o$) vs. *P/P*$_o$ of Cu_{24}(5-decoxy-bdc)$_{24}$ (top right). According to the first BET consistency criterion, the BET linear fit can be determined via the maximum *P/P*$_o$, which is indicated by the dashed line. The slope of the line (bottom) where $P/P_o < 0.24$ is 0.993 with a y-intercept of 0.0623. These values satisfy the second BET consistency criterion and yield a CO_2 BET surface area of 103.096 m^2/g.

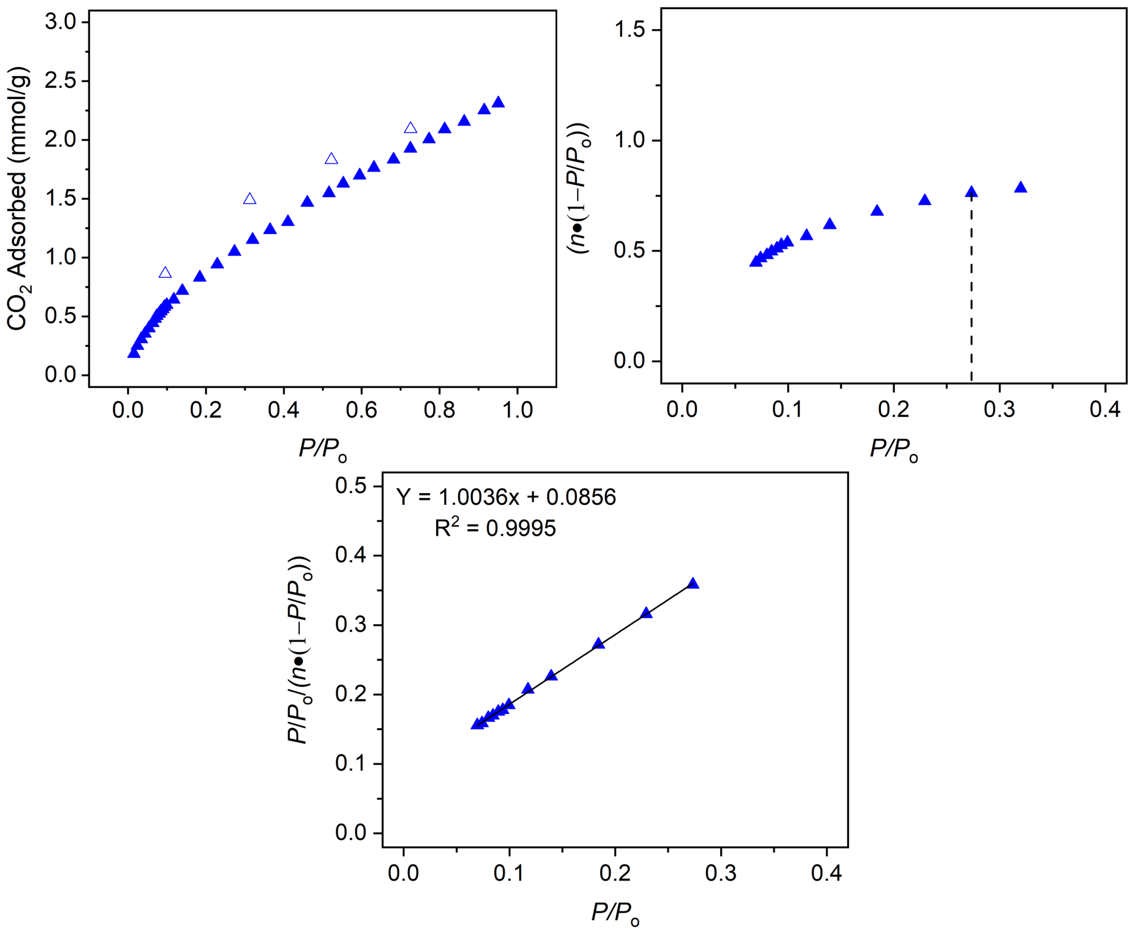

Figure S103. Full BET isotherm (top left) of Cu_{24}(5-undecoxy-bdc)$_{24}$ cage (CO_2) activated at 75 °C under flowing N_2. Plot of n•(1-P/P_o) vs. P/P_o of Cu_{24}(5-undecoxy-bdc)$_{24}$ (top right). According to the first BET consistency criterion, the BET linear fit can be determined via the maximum P/P_o, which is indicated by the dashed line. The slope of the line (bottom) where $P/P_o < 0.28$ is 1.0036 with a y-intercept of 0.0856. These values satisfy the second BET consistency criterion and yield a CO_2 BET surface area of 102.007 m^2/g.

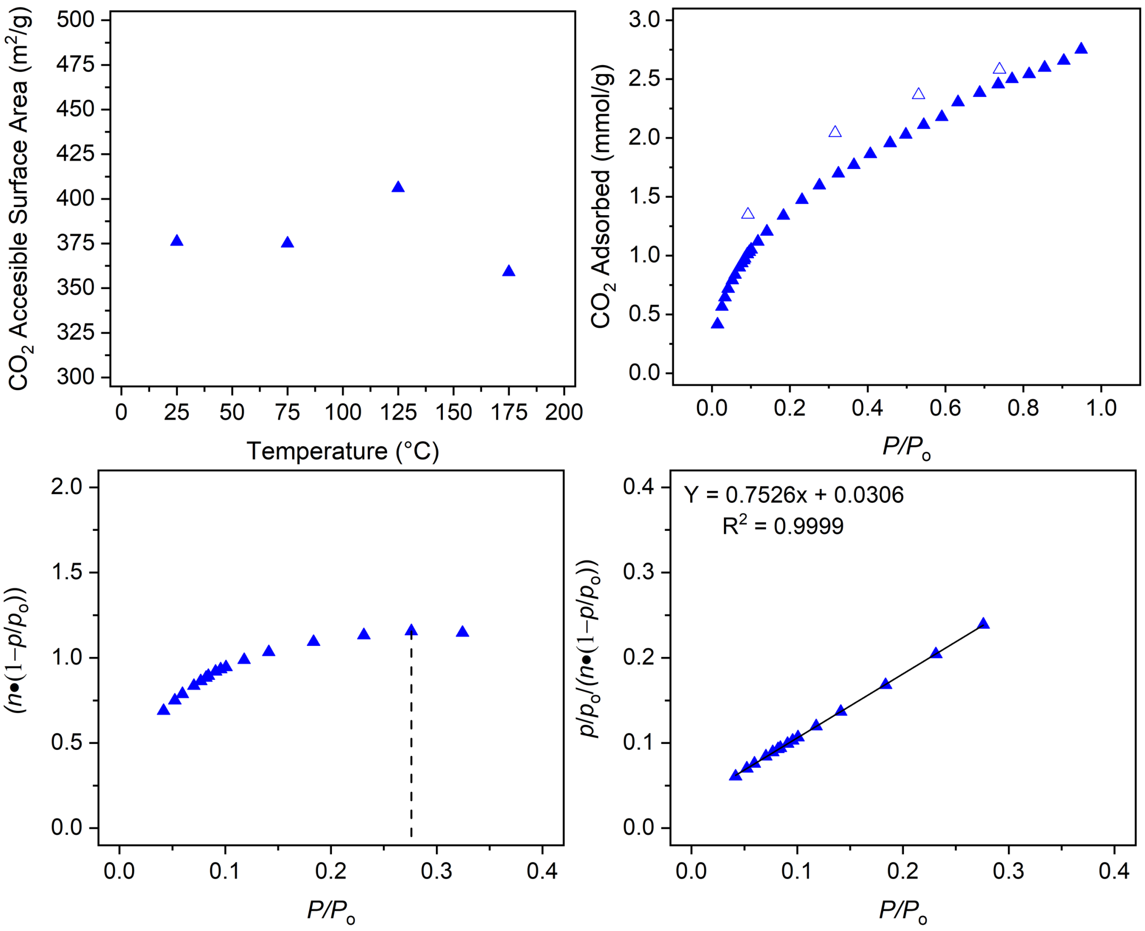

Figure S104. Degas survey (top left) and full BET isotherm (top right) of Cu_{24}(5-dodecoxy-bdc)$_{24}$ cage (CO_2) activated at 125 °C under flowing N_2. Plot of n•(1-P/P_o) vs. P/P_o of Cu_{24}(5-dodecoxy-bdc)$_{24}$ (bottom left). According to the first BET consistency criterion, the BET linear fit can be determined via the maximum P/P_o, which is indicated by the dashed line. The slope of the line (bottom right) where P/P_o< 0.28 is 0.7526 with a y-intercept of 0.0306. These values satisfy the second BET consistency criterion and yield a CO_2 BET surface area of 136.027 m^2/g.

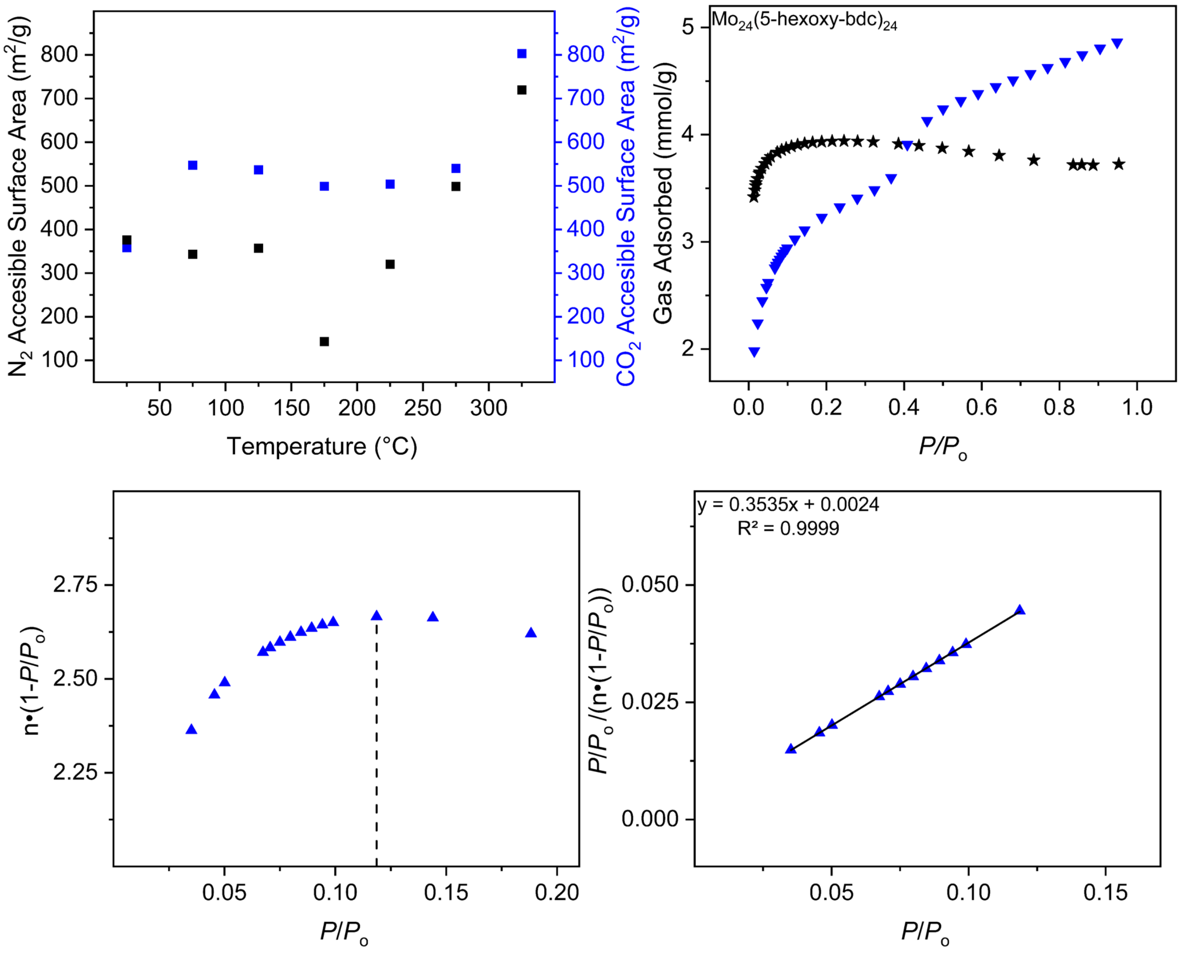

Figure S105. Degas survey (top left) and full BET isotherm (top right) of Mo_{24}(5-hexoxy-bdc)$_{24}$ cage (CO_2) activated at 25 °C under vacuum. Plot of n•(1-P/P_o) vs. P/P_o of Mo_{24}(5-nonoxy-bdc)$_{24}$ (bottom left). According to the first BET consistency criterion, the BET linear fit can be determined via the maximum P/P_o, which is indicated by the dashed line. The slope of the line (bottom right) where $P/P_o < 0.11$ is 0.3535 with a y-intercept of $2.4 \cdot 10^{-3}$. These values satisfy the second BET consistency criterion and yield a CO_2 BET surface area of 289.601 m^2/g.

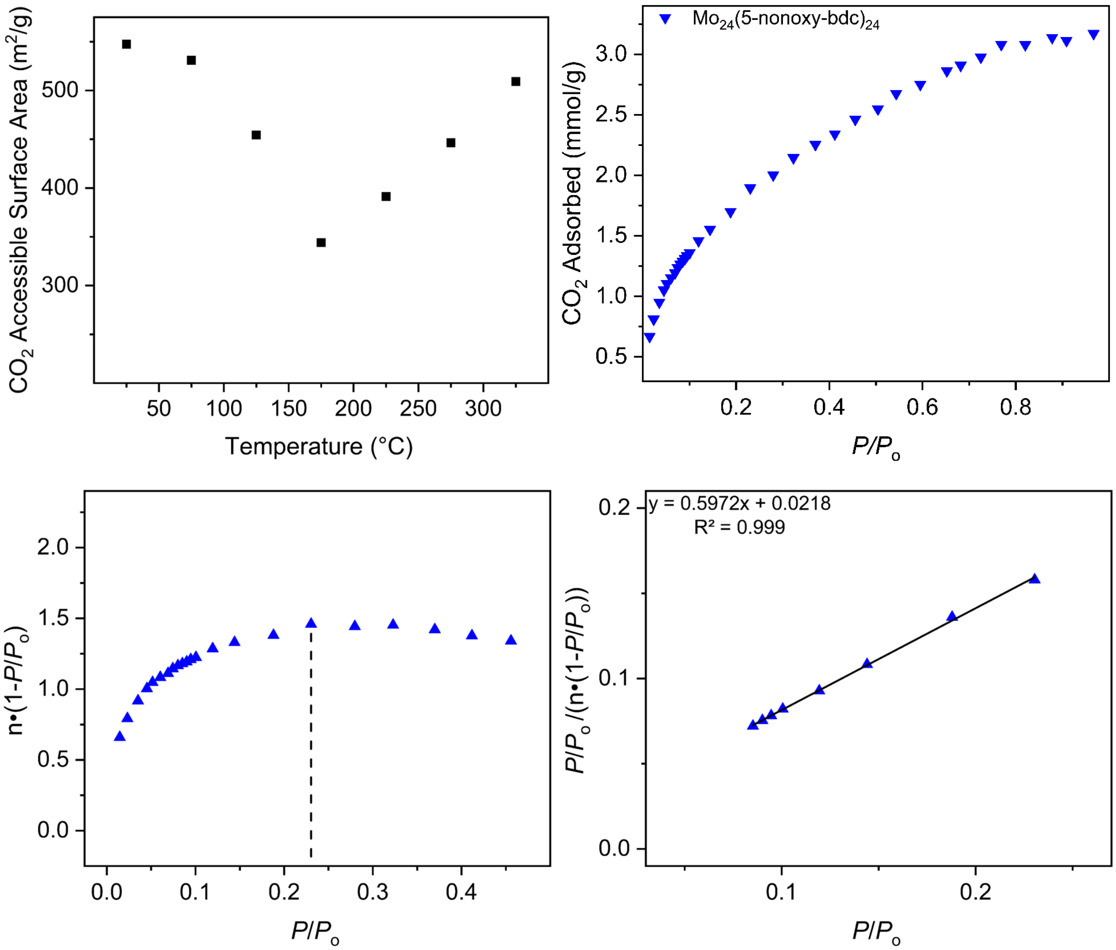

Figure S106. Degas survey (top left) and full BET isotherm (top right) of Mo_{24}(5-nonoxy-bdc)$_{24}$ cage (CO_2) activated at 25 °C under vacuum. Plot of n•(1-P/P_o) vs. P/P_o of Mo_{24}(5-nonoxy-bdc)$_{24}$ (bottom left). According to the first BET consistency criterion, the BET linear fit can be determined via the maximum P/P_o, which is indicated by the dashed line. The slope of the line (bottom right) where P/P_o< 0.23 is 0.5972 with a y-intercept of 2.1 • 10^{-2}. These values satisfy the second BET consistency criterion and yield a CO_2 BET surface area of 171.423 m^2/g.

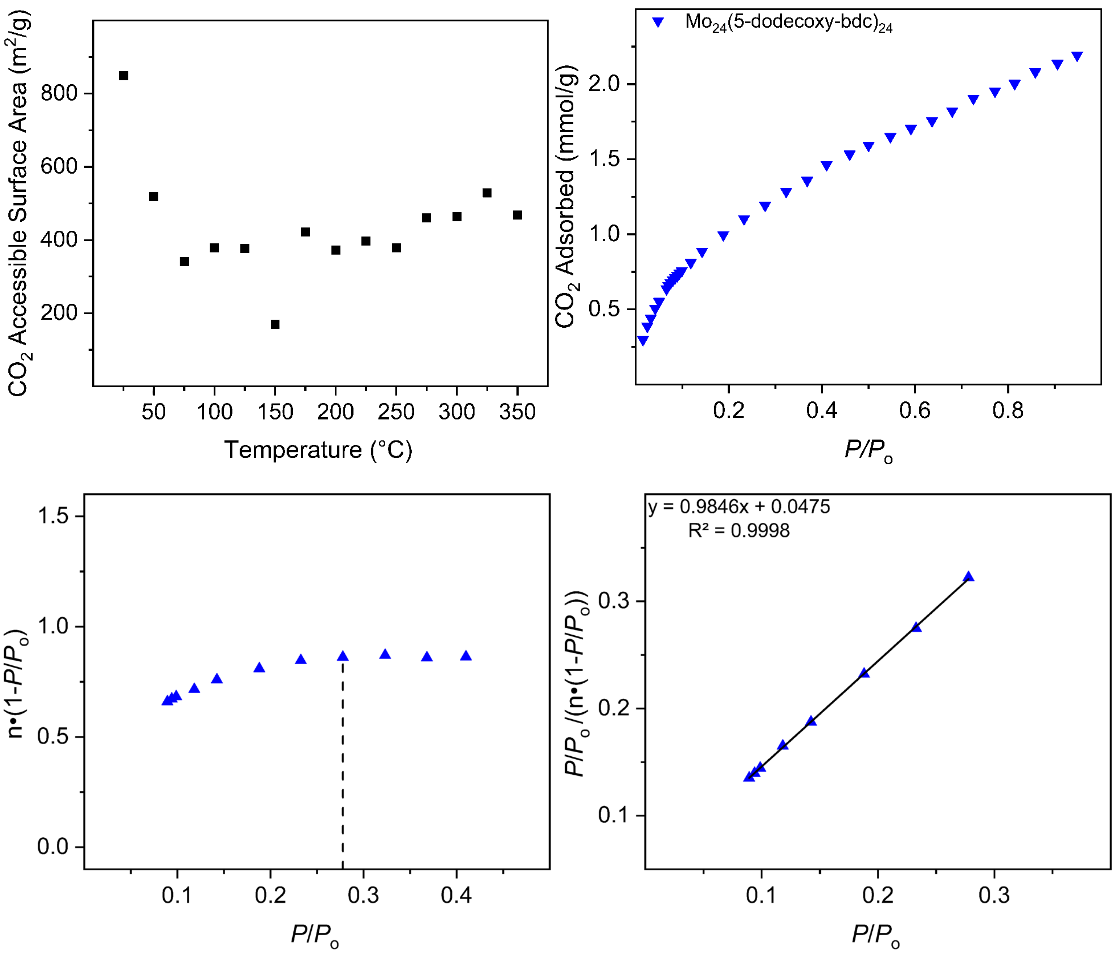

Figure S107. Degas survey (top left) and full BET isotherm (top right) of Mo_{24}(5-dodecoxy-bdc)$_{24}$ cage (CO_2) activated at 25 °C under vacuum. Plot of n•(1-P/P_o) vs. P/P_o of Mo_{24}(5-dodecoxy-bdc)$_{24}$ (bottom left). According to the first BET consistency criterion, the BET linear fit can be determined via the maximum P/P_o, which is indicated by the dashed line. The slope of the line (bottom right) where $P/P_o < 0.28$ is 0.9846 with a y-intercept of $4.8 \cdot 10^{-2}$. These values satisfy the second BET consistency criterion and yield a CO_2 BET surface area of 103.975 m^2/g.

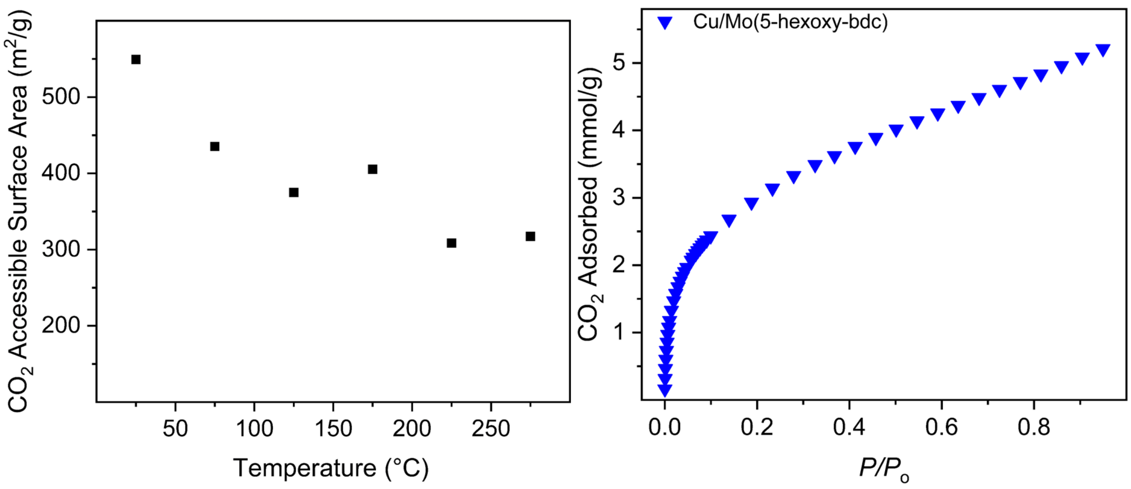

Figure S108. Degas survey (top left) and full BET isotherm (top right) of Cu/Mo(5-hexoxy-bdc) alloy (CO_2) activated at 25 °C under vacuum.

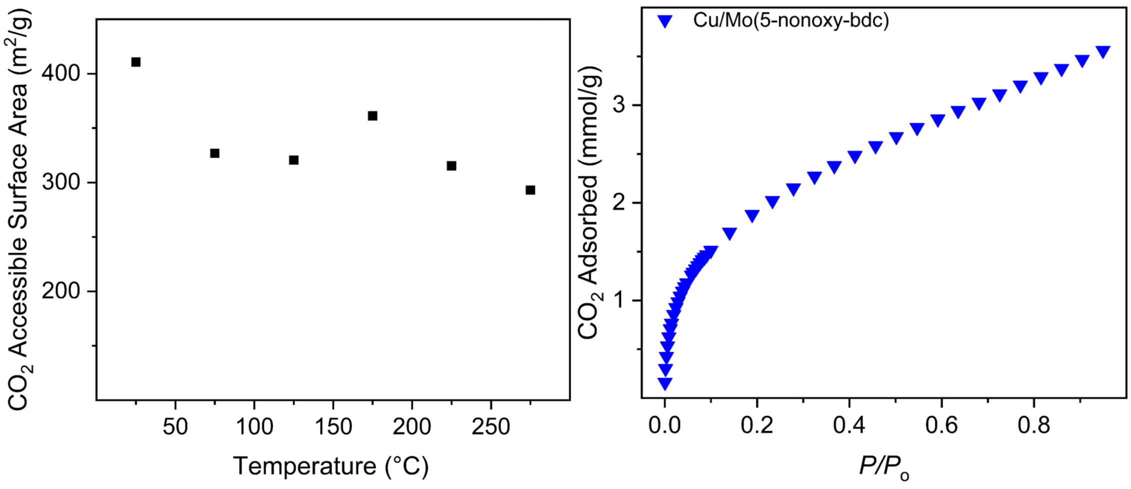

Figure S109. Degas survey (top left) and full BET isotherm (top right) of Cu/Mo(5-nonoxy-bdc) alloy (CO_2) activated at 25 °C under vacuum.

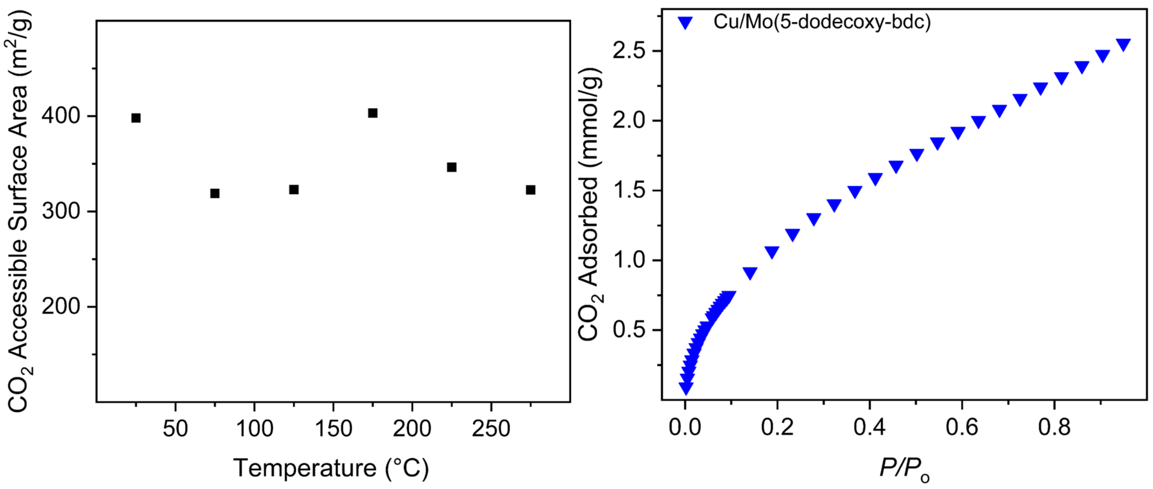

Figure S110. Degas survey (top left) and full BET isotherm (top right) of Cu/Mo(5-dodecoxy-bdc) alloy (CO_2) activated at 25 °C under vacuum.

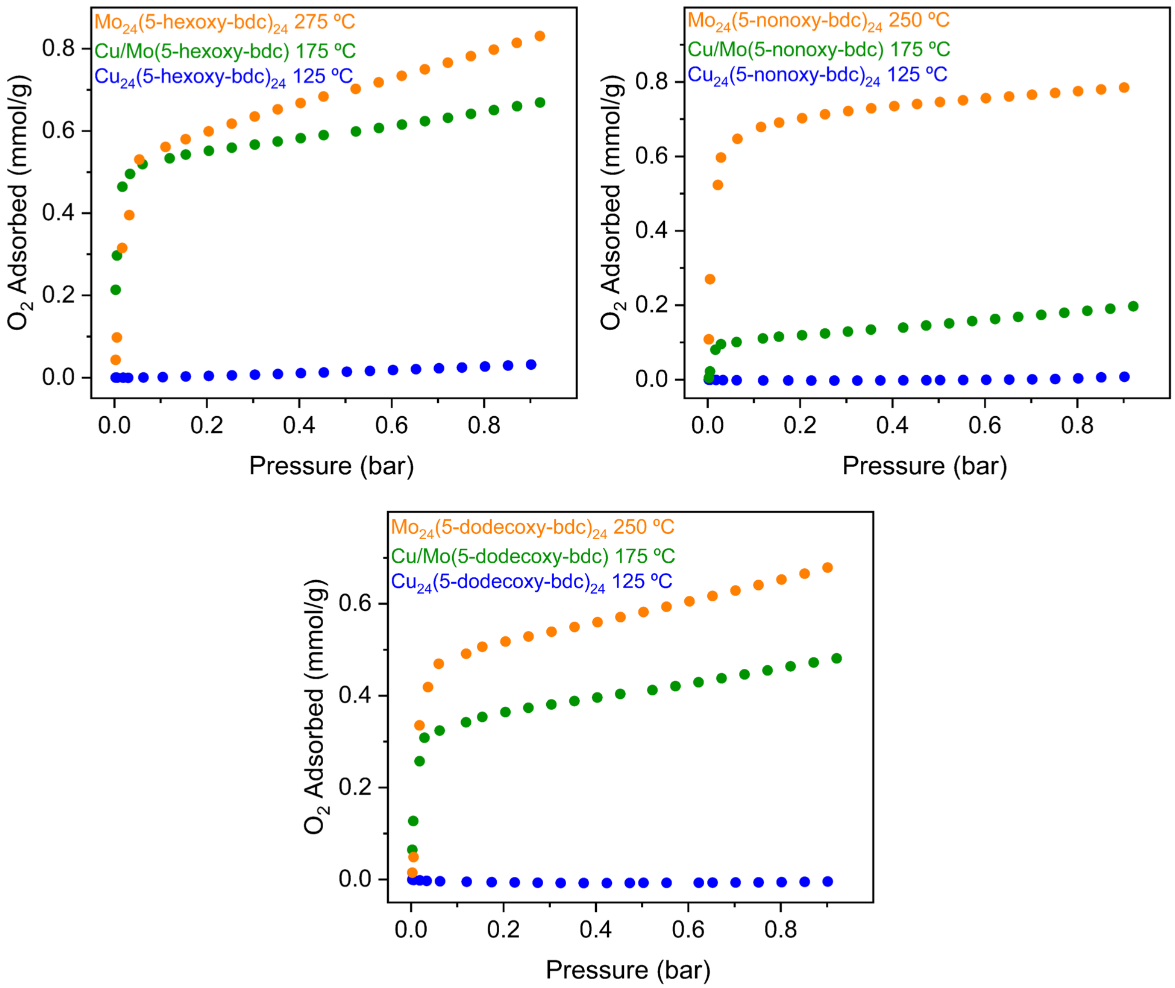

Figure S111. O_2 uptake comparing the respective Mo(II) alkoxy cage, Cu/Mo cage alloy, and Cu(II) alkoxy cage after activation at the appropriate temperatures.

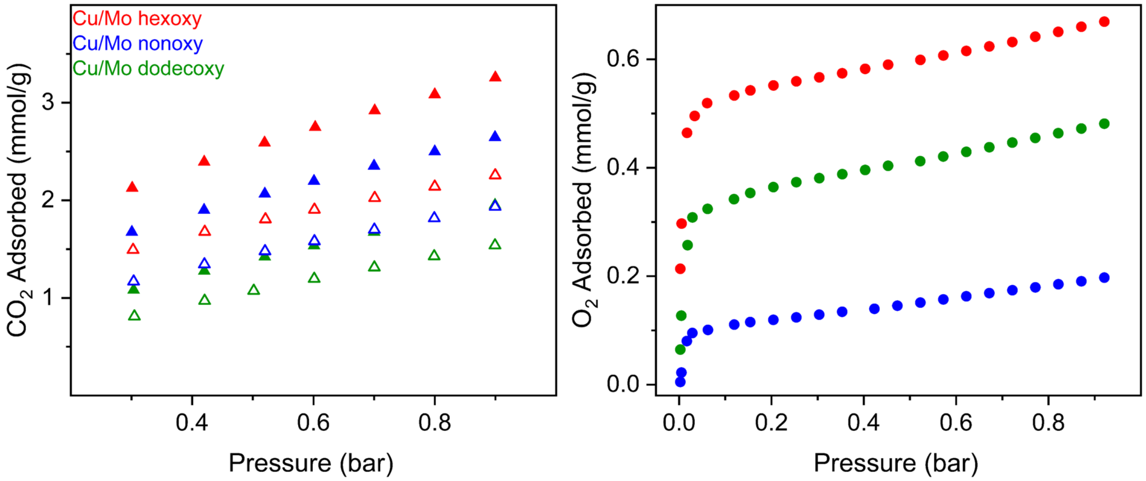

Figure S112. Langmuir CO_2 surface area and O_2 uptake of Cu/Mo(5-hexoxy-bdc) (red), Cu/Mo(5-nonoxy-bdc) (blue) and Cu/Mo(5-dodecoxy-bdc) (green) activated at 175 ºC (closed symbols) vs reactivated at 175 ºC post O_2 dosing (open symbols).

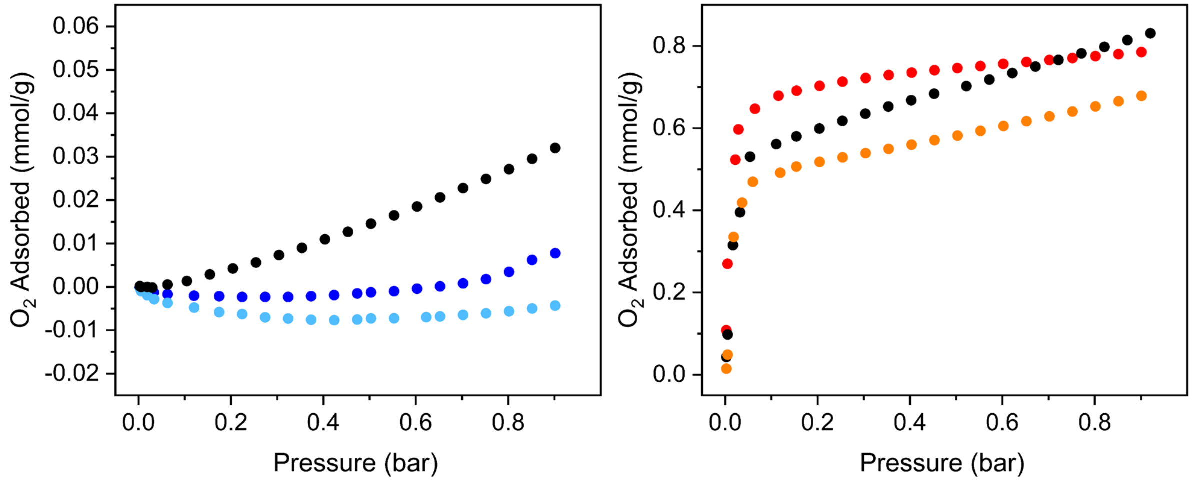

Figure S113. O_2 uptake of Cu_{24}(5-hexoxy-bdc)$_{24}$ (black), Cu_{24}(5-nonoxy-bdc)$_{24}$ (dark blue) and Cu_{24}(5-dodecoxy-bdc)$_{24}$ (light blue) activated at 125 ºC under flowing N_2 (left). O_2 uptake of Mo_{24}(5-hexoxy-bdc)$_{24}$ (black), Mo_{24}(5-nonoxy-bdc)$_{24}$ (red) and Mo_{24}(5-dodecoxy-bdc)$_{24}$ (orange) activated at either 275 ºC or 250 ºC under vacuum.

Surface Areas:

Table S2. Langmuir and BET Surface Areas of Cu(II), Mo(II) and Cu/Mo Alloys

	**Copper (II)**		**Molybdenum (II)**		**Cu/Mo**	
**Ligand**	Langmuir (m^2/g)	BET (m^2/g)	Langmuir (m^2/g)	BET (m^2/g)	Langmuir (m^2/g)	BET (m^2/g)
5-hexoxy-bdc	90 (N_2)<br>523 (CO_2)	197 (CO_2)	366 (N_2)<br>358 (CO_2)	355 (N_2)<br>290 (CO_2)	549 (CO_2)	267 (CO_2)
5-heptoxy-bdc	334 (CO_2)	168 (CO_2)	-	-	-	-
5-octoxy-bdc	771 (CO_2)	182 (CO_2)	-	-	-	-
5-nonoxy-bdc	363 (CO_2)	125 (CO_2)	404 (CO_2)	172 (CO_2)	293 (CO_2)	175 (CO_2)
5-decoxy-bdc	365 (CO_2)	103 (CO_2)	-	-	-	-
5-undecoxy-bdc	374 (CO_2)	102 (CO_2)	-	-	-	-
5-dodecoxy-bdc	354 (CO_2)	136 (CO_2)	294 (CO_2)	104 (CO_2)	398 (CO_2)	121 (CO_2)

Table S3. Langmuir CO_2 Surface Areas of Mo(II) cages post 250 ºC activation before and after O_2 dosing

Mo(II) cage	Langmuir SA (m^2/g)	Langmuir SA post O_2 (m^2/g)
Mo_{24}(5-hexoxy-bdc)$_{24}$	592	476
Mo_{24}(5-nonoxy-bdc)$_{24}$	366	349
Mo_{24}(5-dodecoxy-bdc)$_{24}$	458	365

Table S4. Langmuir CO_2 Surface Areas of Cu/Mo alloys post 175 ºC activation before and after O_2 dosing

Cu/Mo alloys	Langmuir SA (m^2/g)	Langmuir SA post O_2 (m^2/g)
Cu/Mo(5-hexoxy-bdc)	382	261
Cu/Mo(5-nonoxy-bdc)	322	250
Cu/Mo(5-dodecoxy-bdc)	286	248

Table S5. Solubility in different solvents of Cu(II) Cages

Cu Cage Solubility	DMF	DMA	THF	$CHCl_3$	DCM	Benzene
Hexoxy	S	S	I	I	I	I
Heptoxy	S	S	S*	I	I	I
Octoxy	S	S	P	P	P	I
Nonoxy	S	S	S*	P	P	I
Decoxy	S	S	P	P	P	I
Undecoxy	S	S	S	S	S	S
Dodecoxy	S	S	S	S	S	S

S: Soluble; P: Partially Soluble; I: Insoluble; *Soluble at 60 ºC

Table S6. Langmuir CO_2 Surface Areas of Cu(II) Cages Post-Solvent Evaporation

Cu(II) Cage	Langmuir SA (m^2/g)	Solvent Used
Hexoxy	443	DMF
Nonoxy	213	THF

References

1. Furukawa, H.; Kim, J.; Plass, K. E.; Yaghi, O. M. "Crystal Structure, Dissolution, and Deposition of a 5 nm Functionalized Metal-Organic Great Rhombicuboctahedron" *J. Am. Chem. Soc.* **2006**, *128*, 8398-8399.
2. Apex3; Bruker AXS Inc.: Madison, WI, 2015.
3. G. M. Sheldrick, *Acta. Cryst.,* 2015, **A71**, 3-8.
4. G. M. Sheldrick, *Acta. Cryst.*, 2015, **C71**, 3-8.
5. A. L. Spek, *Acta. Cryst*., 2015, **C71**, 9-18.
6. Walton, K. S.; Snurr, R. Q. "Applicability of the BET Method for Determining Surface Areas of Microporous Metal-Organic Frameworks" *J. Am. Chem. Soc.* **2007**, *129*, 8552-8556.
